# The Synthesis of α-Hydroxy-Alkylphosphonate Derivatives and Evaluation of Their Cytotoxic Activity

**DOI:** 10.3390/ph19030396

**Published:** 2026-02-28

**Authors:** Zsuzsanna Szalai, Regina Facskó, Ágnes Gömöry, László Drahos, Szilárd Tekula, Angéla Takács, László Kőhidai, György Keglevich

**Affiliations:** 1 Department of Organic Chemistry and Technology, Faculty of Chemical Technology and Biotechnology, Budapest University of Technology and Economics, Műegyetem rkp. 3., 1111 Budapest, Hungary; szalai.zsuzsanna@edu.bme.hu (Z.S.); facskor@edu.bme.hu (R.F.); 2 MS Proteomics Research Group, Research Centre for Natural Sciences, 1117 Budapest, Hungary; gomory.agnes@ttk.hu (Á.G.);; 3 Department of Genetics, Cell- and Immunobiology, Semmelweis University, Nagyvárad tér 4., 1089 Budapest, Hungarytakacs.angela@semmelweis.hu (A.T.); kohidai.laszlo@semmelweis.hu (L.K.)

**Keywords:** α-hydroxy-alkylphosphonates, Pudovik reaction, acylation, phosphinoylation, phosphorylation, thiophosphinoylation, sulfonylation, cytotoxicity, myeloma (U266) cells, melanoma (A2058) cells

## Abstract

**Background**: It is known that the α-hydroxyphosphonates and their derivatives may have potential biological activity. **Methods**: Within the prominent class of α-hydroxyphosphonates, α-hydroxy-alkylphosphonates and their derivatives were prepared as new representatives in the hope of obtaining biologically active species. During our work the Pudovik reaction, acylation and phosphinoylation/phosphorylation methods were used. The new compounds were characterized by NMR and MS spectroscopy. The antiproliferative effects were tested on U266 (myeloma multiplex) and A2058 (melanoma) cells. **Results**: Ethyl methyl ketone–dialkyl phosphite and secondary phosphine oxide adducts were synthesized by the Pudovik reaction on the earlier analogy of acetaldehyde– and acetone adducts. The hydroxyphosphonates and hydroxyphosphine oxides were acylated and phosphinoylated/phosphorylated. Due to the steric hindrance in the case of the preparation of the acetone–and ethyl methyl ketone–diethyl phosphite adducts, a two-step procedure was elaborated that was also suitable for the thiophosphinoylation of the adducts. A part of the α-hydroxyphosphonates could be successfully methanesulfonylated. The new derivatives prepared were tested on myeloma and melanoma cells, and it was found that the antiproliferative activity is primarily driven by phosphinoylation, particularly by diphenylthiophosphinoylation. The most promising compound, the diphenylthiophosphinoylated hydroxyphosphine oxide, reduced the viability of the U266 cells to less than 20% after a treatment with 100 µM concentration in a long-term experiment. **Conclusions**: A subset of the synthesized α-hydroxyphosphonate derivatives exhibited cytotoxic activity, supporting further structural optimization to identify compounds with enhanced biological efficacy.

## 1. Introduction

Within the group of phosphonates, α-substituted derivatives form an important class. This work focuses on the group of α-hydroxyphosphonates for two reasons. On the one hand, their synthesis by the Pudovik reaction of oxo compounds and >P(O)H reagents, mainly dialkyl phosphites, is an ideal model for organic chemists [1,2,3,4,5,6], especially from the point of view of “green” chemistry [7,8,9,10,11,12]. The elaboration of catalyst-free [13] and solvent-free methods [9,10,11,12,13,14,15,16,17,18,19] along with microwave-assisted protocols [20,21] marked a milestone in the history of the classical Pudovik reaction. On the other hand, α-hydroxyphosphonates and their derivatives exhibit potential or real biological activity due to their enzyme-inhibitory properties [22,23,24,25]. They may typically display pesticide [26,27,28,29], and within this, insecticide activity [30,31,32]. Furthermore, they may have antibacterial [33,34], antimicrobial [35,36], antiviral [37], antifungal [38], antioxidant [39,40,41], and antitumor [42,43,44,45] effects. Cancer remains one of the most significant public health challenges in Europe. According to the European Cancer Information System, approximately 2.7 million new cancer cases were recorded across the European Union in 2024, and about 1.27 million deaths were attributed to cancer in the same year. Overall, these data indicate a slight decrease in both incidence and mortality compared with the data in 2022; however, the absolute numbers remain substantial, making cancer the second leading cause of death after cardiovascular diseases. This situation highlights the need for intensified research to find novel antiproliferative molecules [46].

**Figure 1:**
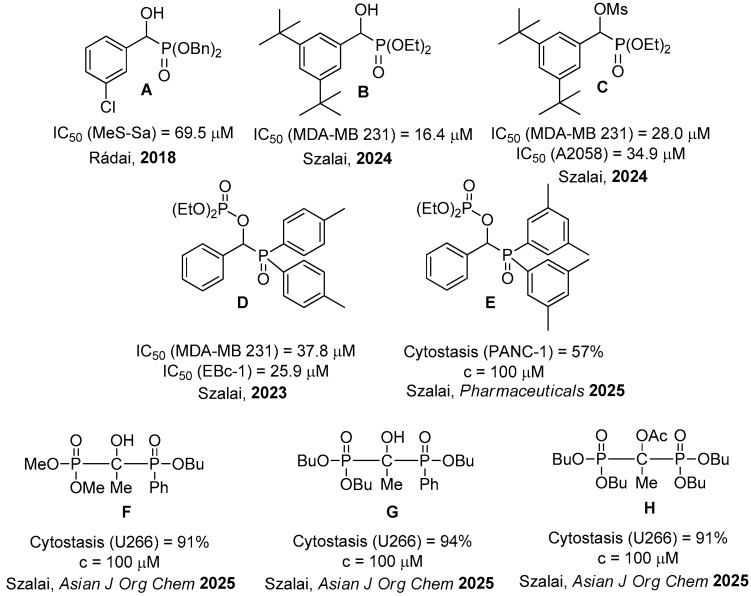
The cytostatic effect of hydroxyphosphonates and related derivatives (**A**–**H**) was studied on human tumor cell cultures. Rádai, **2018** [47], Szalai, **2024** [48], Szalai, **2023** [49], Szalai, *Pharmaceuticals* **2025** [50], Szalai, *Asian J. Org. Chem.* **2025** [51].

The cytotoxic effects of substituted α-hydroxy-benzylphosphonates, their P-acid derivatives and their O-phosphinoylated variations were evaluated by us on the Mes-Sa uterine sarcoma cell line, and a few derivatives were found toxic against the multidrug-resistant cell line (see e.g., Figure 1/species **A**) [47]. The α-hydroxy- and α-mesyloxy-di-tert-butylbenzylphosphonates revealed significant activity on breast carcinoma and melanoma cell lines (**B** and **C**) [48]. Phosphorylated α-hydroxy-benzyl-diarylphosphine oxides **D** and **E** displayed a cytotoxic effect on breast carcinoma/small cell lung cancer cells, and on pancreatic cancer cells, respectively [49,50].

To date, there is not much data on the cytotoxic activity of the members of a new family of compounds, namely α-hydroxy-alkylphosphonates. Just a few α-hydroxyphosphine oxides and α-hydroxyphosphonates were tested on myeloma and pancreatic adenocarcinoma cell cultures [52]. As the results obtained, especially on the myeloma cell line, were encouraging, we decided to expand the members of the family of α-hydroxy-alkylphosphonates and their derivatives, including acylated, phosphorylated and sulfonylated species, in the hope of making available more active species.

## 2. Results and Discussion

### 2.1. Synthesis

#### 2.1.1. Preparation of the α-Hydroxy-Alkylphosphonates

**Scheme 1:**
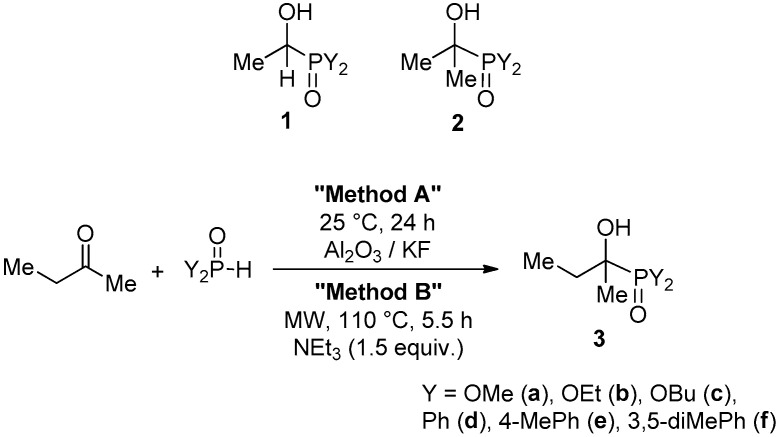
Pudovik reaction of ethyl methyl ketone with >P(O)H reagents.

In the course of our earlier work, acetaldehyde–dialkyl phosphite and acetaldehyde–diarylphosphine oxide adducts (**1**) were prepared at 0 °C, promoted by triethylamine in ethyl acetate [52]. The Pudovik reaction of acetone and dialkyl phosphites was carried out on the surface of Al_2_O_3_/KF at room temperature to afford the corresponding adducts (**2a** and **2b**) [52]. At the same time, the similar reactions with diarylphosphine oxides to prepare the corresponding hydroxyphosphine oxides (**2d**) required more forcing conditions: MW irradiation at 110 °C and Na_2_CO_3_ as the catalyst in a solvent-free manner [52]. Starting from ethyl methyl ketone, the new hydroxyphosphonates (**3a–c**) and hydroxyphosphine oxides (**3d–f**) were synthesized on the analogy of the acetone–Y_2_P(O)H reagent adducts (Y = alkoxy or aryl) applying the solid phase synthesis (Method A/Scheme 1) or the MW-assisted approach (Method B/Scheme 1). The corresponding α-hydroxy-α-methyl-propylphosphonates (**3a–c**) and α-hydroxy-α-methyl-propylphosphine oxides (**3d–f**) were obtained in 40–79% yields after purification by column chromatography. The moderate yields are the consequences of the small-scale experiments. Esters **3a** and **3b** have been described earlier [53,54]. While the dimethyl ester (**3a**) was prepared by a direct copper-catalyzed hydroxylation [53], the ethyl ester (**3b**) was obtained via the Pudovik reaction. However, the latter route performed at 80 °C in the presence of NaOEt in EtOH furnished product **3b** in only 15% [54]. It can be concluded that our methods represented in Scheme 1 are more advantageous with respect to simplicity and efficiency. Dibutyl ester **3c** is a new compound. From among the hydroxyphosphine oxides (**3d–f**), the diphenyl derivative **3d** was described earlier [55], but the other two species (**3e** and **3f**) were new. It is noteworthy that the bis(3,5-dimethylphenyl) derivative (**3f**) was unstable and started decomposing immediately after the synthesis. According to LC-MS, the main components of the decomposition were ethyl methyl ketone ([M + H]^+^ = 73) and bis(3,5-dimethylphenyl)phosphinic acid ([M + H]^+^ = 259), the latter species being formed from bis(3,5-dimethylphenyl)phosphine oxide by oxidation. The primary decomposition was in accord with the reversible formation of the adduct. It is known that the ketone–secondary phosphine oxide adducts are not stable [56]. An electron-donating substituent in the aromatic ring may increase the instability. In all, the new derivatives **3c** and **3e** were fully characterized by ^31^P, ^13^C and ^1^H NMR, as well as HRMS, while the known species **3a**, **3b** and **3d** were identified by comparison of their ^31^P NMR shifts with literature values, and by HRMS. The unstable compound **3f** was identified by ^31^P NMR and HRMS. The phosphonates **3a–c** appear at around δ_P_ 28–30, while the phosphine oxides **3d–f** appear at δ_P_ 35–37.

#### 2.1.2. Acylation of the α-Hydroxy-Alkylphosphonates

**Scheme 2:**
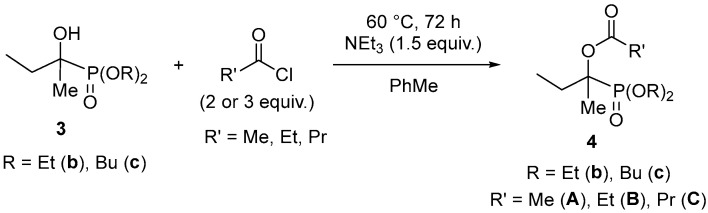
The acylation of α-hydroxy-α-methyl-propylphosphonates (**3b** and **3c**) with carboxylic acid chlorides.

Then, we modified two of the α-hydroxy-α-methyl-propylphosphonates (**3b** and **3c**) by acylation using acetyl chloride, propionyl chloride and butyryl chloride at 60 °C, applying triethylamine as the base in toluene. Acetyl chloride was applied in a somewhat larger excess (Scheme 2). The work-up, including chromatography, provided the acylated hydroxyphosphonates (**4bA**, **4bB**, **4bC** and **4cA**) in 48–66% yields. The moderate yields are the consequences of the small-scale experiments. The products (**4bA–C** and **4cA**) were characterized by ^31^P, ^13^C and ^1^H NMR, as well as HRMS. The acylated hydroxyphosphonates **4bA–C** and **4cA** appear at around δ_P_ 23.

**Figure 2:**
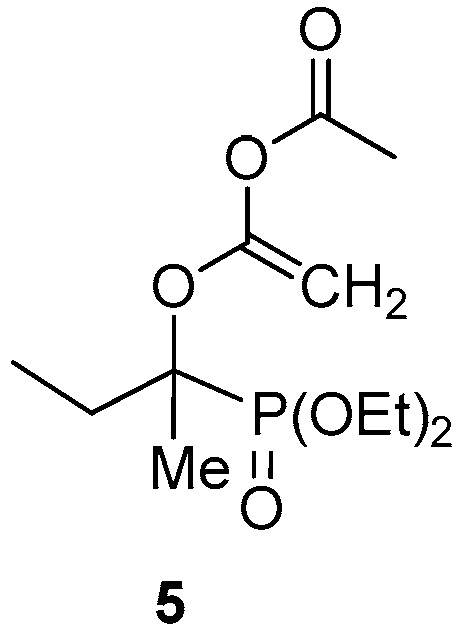
An interesting by-product from the reaction of hydroxyphosphonate **3b** and acetyl chloride.

It is worth mentioning that in the crude mixture of the reaction of hydroxyphosphonate **3b** with acetyl chloride, the presence of bisacylated derivative, probably **5**, could also be pointed out by ^31^P NMR (δ_P_ = 22.4 (CDCl_3_)), LC-MS ([M + H]^+^ = 295) and HRMS ([M + Na]^+^_found_ = 317.1125, C_12_H_23_O_6_PNa requires 317.1130). It is assumed that the enol form of the primary product is acylated on the OH group. (Figure 2).

#### 2.1.3. Phosphinoylation and Phosphorylation of the α-Hydroxy-Alkylphosphonates

**Scheme 3:**
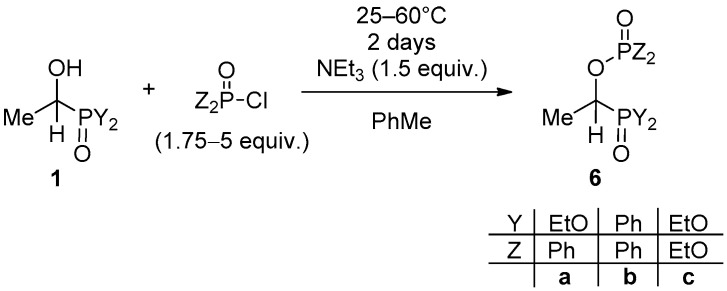
Phosphorylation of α-hydroxy-ethylphosphonate **1b** and α-hydroxy-ethylphosphine oxide **1d**.

After the acylations, the phosphorylation of the hydroxy-alkylphosphonate **1b** and hydroxy-alkylphosphine oxide **1d** with diphenylphosphinic chloride and diethyl chlorophosphate was aimed at in the presence of triethylamine as the base and in toluene as the solvent at 25 °C and 60 °C, respectively (Scheme 3). The phosphinoylated hydroxyphos-phonates **6a** and **6b**, as well as the phosphorylated analogue **6c**, were obtained in yields of 52–88% after purification by column chromatography. The new products **6a** and **6b** were fully characterized as needed, but the earlier described **6c** was identified only by ^31^P NMR and HRMS. The ^31^P NMR spectra of species **6a–c** exhibit two doublets with a *J*(P,P) of 26–32 Hz.

**Scheme 4:**
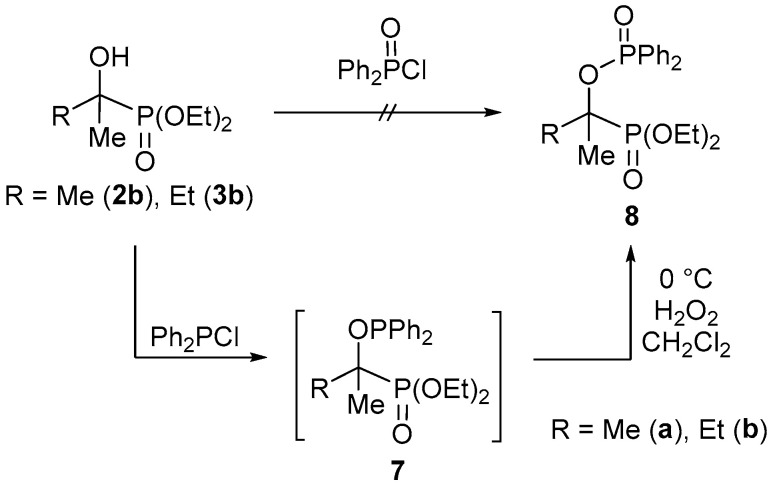
Phosphinoylation of α-hydroxy-alkylphosphonates **2b** and **3b**.

Going further, the acetone and ethyl methyl ketone adducts **2b** and **3b** were also subjected to phosphinoylation by diphenylphosphinic chloride. However, the reaction failed even at 60 °C. Therefore, the less sterically hindered diphenylphosphinous chloride was applied in the second round at 60 °C using triethylamine as the base in toluene. In a one-pot procedure, the sensitive phosphinous ester intermediate (**7**) was stabilized by oxidation, affording the expected phosphinoylated α-hydroxyphosphonates **8a** and **8b** (Scheme 4). The products (**8a** and **8b**) obtained after purification by chromatography were characterized by NMR and MS. In the ^31^P NMR spectra of compounds **8a** and **8b**, one pair of doublets (in each) was observed, with a coupling of 35 Hz and 32 Hz, respectively.

#### 2.1.4. Thiophosphinoylation of the α-Hydroxy-Alkylphosphonates

**Scheme 5:**
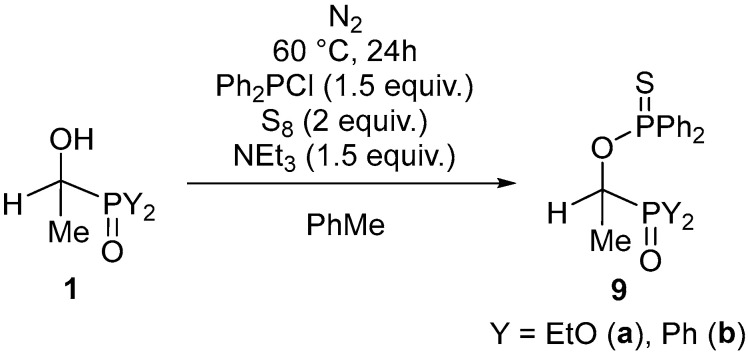
Thiophosphinoylation of α-hydroxy-ethylphosphonate **1b** and α-hydroxy-ethylphosphine oxide **1d**.

Then, imitating the successful protocol shown above, the one-pot transformation of the acetaldehyde–>P(O)H reagent adducts was repeated, applying elemental sulfur as the blocking agent (Scheme 5). The products (**9a** and **9b**) isolated in 94/76% yields on purification were fully characterized.

**Scheme 6:**
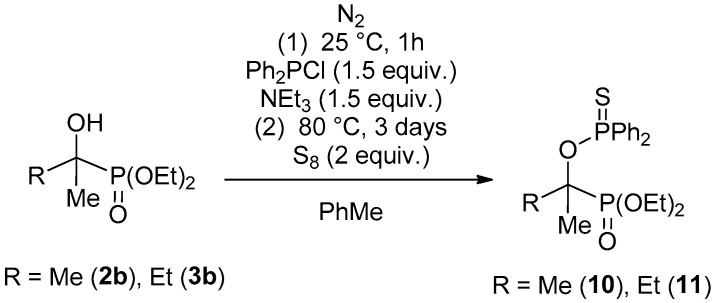
Inefficient thiophosphinoylation of α-hydroxy-alkylphosphonates **2b** and **3b**.

However, probably as a consequence of steric hindrance, this protocol could not be adopted for the acetone and ethyl methyl ketone adducts. The expected products (**10** and **11**) were formed in a very low conversion; hence, they could be identified just by ^31^P NMR and MS (Scheme 6). The ^31^P NMR spectra of the “P=O–P=S” compounds **8a**, **8b**, **9a** and **9b** displayed the typical two doublets of 30–35 Hz.

#### 2.1.5. Methanesulfonylation of the α-Hydroxy-Alkylphosphonates

**Scheme 7:**
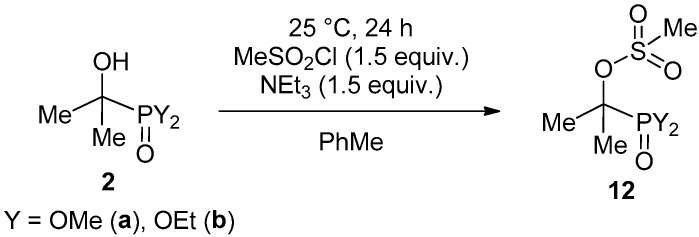
Methanesulfonylation of acetone–dialkyl phosphite adducts **2a** and **2b**.

Last but not least, the acetone–dialkyl phosphite adducts **2a** and **2b** were subjected to reaction with methanesulfonyl chloride at 25 °C in the presence of triethylamine in toluene as the solvent (Scheme 7). After flash column chromatography, the methanesulfonyloxy-alkylphosphonates (**12a** and **12b**) were obtained in 81/79% yields, and were fully characterized by NMR and MS.

### 2.2. Study on the Cytotoxic Effect of the Phosphonates and Phosphine Oxides Prepared

**Figure 3:**
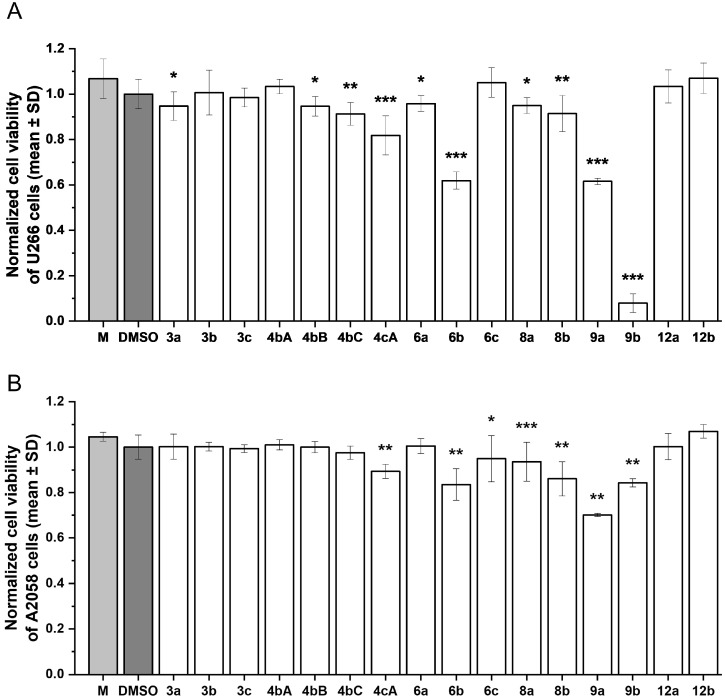
In vitro antiproliferative effects of compounds tested at 100 µM on U266 (**A**) and on A2058 (**B**) cell lines after 72 h. The data are normalized to the vehicle DMSO control. Data represented as the mean ± SD; *n* = 3. The levels of significance are shown as follows: *: *p* < 0.05; **: *p* < 0.01; ***: *p* < 0.001, determined by the one-way ANOVA test, followed by Fisher’s LSD post hoc test.

In the cell viability assays, the α-hydroxy-α-methyl-propylphosphonates **3a–c** showed minimal effects on the viability of both myeloma (U266) and melanoma (A2058) cells (Figure 3A,B). The acylated hydroxyphosphonates (**4bA**, **4bB**, **4bC**, and **4cA**) caused no reduction in the viability of melanoma (A2058) cells; however, they exhibited modest, compound-dependent activity on myeloma (U266) cells. The phosphinoylated and phosphorylated hydroxyphosphonates (**6a** and **6c**) also demonstrated modest cytotoxic activity. However, the phosphinoylated hydroxy-diphenyl-ethylphosphine oxide **6b** significantly reduced the U266 cell viability (0.61 ± 0.03). The additional phosphinoylated α-hydroxyphosphonates **8a** and **8b**, lacking an α-CH unit on the carbon atom, appeared to be ineffective in terms of antiproliferative activity, as no reduction in cell viability was observed. Notably, the thiophosphinoylated hydroxy-species **9a** and **9b** showed the strongest antiproliferative effects overall. Phosphonic ester **9a** exerted comparable effects on both cell lines (U266: 0.61 ± 0.014; A2058: 0.70 ± 0.01), while phosphine oxide **9b** induced an additional pronounced decrease in the U266 cell viability, making it the most potent derivative in the series investigated. In contrast, the methanesulfonyloxy-alkylphosphonates **12a** and **12b** showed no effect on cell viability. In our previous work, the IC_50_ values of clinically relevant anticancer agents, including bortezomib [57] and daunorubicin [58], were determined for comparative purposes. Both compounds exhibited IC_50_ values in the low nanomolar range on A2058 melanoma and U266 cells after 72 h of treatment. In contrast, the compounds investigated in the present study are expected to demonstrate IC_50_ values in the micromolar range; thus, further structural modifications are needed to enhance the antiproliferative effects of the new compounds.

Based on our results, the parent α-hydroxy-α-methyl-propylphosphonates (**3a–c**) were inactive in both cell lines, indicating that the basic hydroxyphosphonate scaffold alone is insufficient to elicit cytotoxic effects. Neither acylation nor phosphinoylation/phosphorylation led to enhanced antiproliferative activity. The first significant increase in cytotoxicity was observed following thiophosphinoylation of hydroxyphosphonate **1b** and hydroxyphosphine oxide **1d**, leading to P=S derivatives **9a** and **9b**, respectively, exhibiting the strongest effects. Compound **9b**, which is a diphenylthiophosphinoylated hydroxyphosphine oxide, was the most effective on U266 cells. These results highlight that the introduction of phenyl groups, as can be seen in **6b**, led to improved potency. The phenyl substitution may be associated with the increased lipophilicity, thus with the improved cellular uptake [59,60]. The improved potency of **6b** was further augmented by the replacement of the oxygen of the P=O function with sulfur, as can be seen in **9b**. This oxygen-sulfur replacement likely alters the electronic properties and polarizability of the phosphorus center, potentially strengthening interactions with possible biological targets, and thus improving cellular activity [60,61]. To clearly identify the target of the potent compounds, further assays are required, which have not yet been performed.

Together, these modifications appear to optimize the hydrophobic character and electronic features simultaneously, leading to superior antiproliferative potency. In contrast, further modification, namely methanesulfonylation, altered the electronic properties of the molecules in a manner that was unfavorable to induce biological activity, resulting in a lack of antiproliferative effects.

## 3. Materials and Methods

### 3.1. General Information

The ^31^P, ^13^C, and ^1^H NMR spectra were taken on a Bruker DRX-500 or Bruker Avance-300 spectrometer (Bruker, Billerica, MA, USA) operating at 202, 126, and 500 MHz or 122, 75, and 300 MHz, respectively. The copies of the ^31^P, ^13^C and ^1^H NMR spectra for compounds **3a–e**, **4bA–C**, **4cA**, **6a**, **6b**, **8a**, **8b**, **9a**, **9b**, **12a** and **12b** preparedcan be seen in the Supplementary Materials. The couplings are given in Hz. HPLC-MS measurements were performed using a Shimadzu LCMS-2020 device (Shimadzu Corporation, Kyoto, Japan) equipped with a Reprospher 100 C18 (5 μm; 100 × 3 mm) column and positive–negative double ion source (DUIS±) with a quadrupole MS analyzer in a range of 50–1000 *m*/*z*. HRMS measurements were carried out on a Q-TOF Premier mass spectrometer (Waters Corporation, Milford, MA, USA) in positive electrospray ionization mode using MassLynx 4.1 software.

### 3.2. General Procedure for the Synthesis of Dialkyl α-Hydroxy-α-methyl-propylphosphonates (***3a–c***) (Method A)

To 11.0 mmol (1.0 mL) of butanone and 5.5 mmol of dialkyl phosphite (dimethyl phosphite: 0.50 mL, diethyl phosphite: 0.70 mL, dibutyl phosphite: 1.0 mL), a mixture of finely powdered 1.3 g of acidic Al_2_O_3_ (Brockmann I.) and 1.3 g of potassium fluoride was added. The reaction mixture was allowed to stand at 26 °C for 24 h. The product was extracted from the solid phase with 4 × 25 mL of ethyl acetate. The solvent was evaporated, and the crude product so obtained was purified by column chromatography on silica gel, applying ethyl acetate as the eluent. Compounds **3a–c** were colorless oils.

#### 3.2.1. Dimethyl α-Hydroxy-α-methyl-propylphosphonate (**3a**)

Yield: 2.4 g (55%); ^31^P {^1^H} NMR (122 MHz, CDCl_3_) δ 29.9; δ_P,lit._ [53] 30.1; [M + H]^+^ = 183; HRMS (ESI) *m*/*z*: [M + Na]^+^ calculated for C_6_H_15_O_4_PNa 205.0606; found 205.0605.

#### 3.2.2. Diethyl α-Hydroxy-α-methyl-propylphosphonate (**3b**)

Yield: 0.52 g (45%); ^31^P {^1^H} NMR (202 MHz, CDCl_3_) δ 27.8; δ_P,lit._ [54] 27.8; [M + H]^+^ = 211; HRMS (ESI) *m*/*z*: [M + Na]^+^ calculated for C_8_H_19_O_4_PNa 233.0919; found 233.0918.

#### 3.2.3. Dibutyl α-Hydroxy-α-methyl-propylphosphonate (**3c**)

Yield: 0.59 g (40%); ^31^P {^1^H} NMR (202 MHz, CDCl_3_) δ 27.6; ^13^C {^1^H} NMR (126 MHz, CDCl_3_) δ 6.8 (d, *J* = 8.4 Hz, CH_2_*C*H_3_), 13.5 (s, CH_2_*C*H_3_), 18.7 (s, *C*H_2_CH_3_), 21.2 (d, *J* = 3.8 Hz, C*C*H_3_), 29.8 (d, *J* = 5.4 Hz, *C*H_2_CH_3_), 32.6 (d, *J* = 5.6 Hz, OCH_2_*C*H_2_), 66.2 and 66.3 (d, *J* = 7.7 Hz, OCH_2_), 71.8 (d, *J* = 161.4 Hz, CP); ^1^H NMR (500 MHz, CDCl_3_) δ 0.96 (t, *J* = 7.4 Hz, 6H, CH_2_C*H*_3_), 1.02 (t, *J* = 7.5 Hz, 3H, CH_2_C*H*_3_), 1.38–1.46 (m, 4H, C*H*_2_CH_3_) overlapped by 1.40 (d, *J* = 15.5 Hz, 3H, CCH_3_), 1.66–1.71 (m, 4H, OCH_2_C*H*_2_), 1.72–1.89 (m, 2H, C*H*_2_CH_3_), 2.21 (bs, 1H, OH), 4.10–4.15 (m, 4H, OCH_2_); [M + H]^+^ = 267; HRMS (ESI) *m*/*z*: [M + Na]^+^ calculated for C_12_H_27_O_4_PNa 289.1548; found 289.1545.

### 3.3. General Procedure for the Synthesis of Diaryl α-Hydroxy-α-methyl-propylphosphine Oxides (***3d–f***) (Method B)

A mixture of 2.0 mmol (0.18 mL) of butanone, 1.0 mmol of diarylphosphine oxide (diphenylphosphine oxide: 0.21 g, bis(4-methylphenyl)phosphine oxide: 0.23 g, bis(3,5-dimethylphenyl)phosphine oxide: 0.26 g) and 1.5 mmol (0.21 mL) of Et_3_N was heated at 110 °C in a vial in a CEM Discover Microwave reactor for 5.5 h. The crude product so obtained was purified by column chromatography on silica gel, applying ethyl acetate as the eluent. Products **3d–f** were obtained as white crystalline compounds.

#### 3.3.1. α-Hydroxy-α-methyl-propyl-diphenylphosphine Oxide (**3d**)

Yield: 0.18 g (65%); m.p.: 124–125 °C; m.p._lit._ [55] 124–126 °C; ^31^P {^1^H} NMR (202 MHz, CDCl_3_) δ 36.7; δ_P,lit._ [55] 34.6; [M + H]^+^ = 275; HRMS (ESI) *m*/*z*: [M + H]^+^ calculated for C_16_H_20_O_2_P 275.1201; found 275.1197; [M + Na]^+^ calculated for C_16_H_19_O_2_PNa 297.1020; found 297.1019.

#### 3.3.2. Bis(4-methylphenyl)(α-hydroxy-α-methyl-propyl)phosphine Oxide (**3e**)

Yield: 0.24 g (79%); m.p.: 111–112 °C; ^31^P {^1^H} NMR (202 MHz, CDCl_3_) δ 35.0; ^13^C {^1^H} NMR (126 MHz, CDCl_3_) δ 6.6 (d, *J* = 8.7 Hz, CH_2_*C*H_3_), 20.7 (d, *J* = 6.3 Hz, C*C*H_3_), 21.5 (s, ArCH_3_), 29.2 (d, *J* = 7.4 Hz, *C*H_2_CH_3_), 74.2 (d, *J* = 88.3 Hz, CP), 127.1 and 127.8 (d, *J* = 2.8 Hz, C_α_), 128.9 and 129.0 (d, *J* = 11.3 Hz, C_β_), 132.4 and 132.5 (d, *J* = 8.4 Hz, C_γ_), 141.7 and 141.8 (d, *J* = 2.9 Hz, C_δ_); ^1^H NMR (500 MHz, CDCl_3_) δ 0.96 (t, *J* = 7.5 Hz, 3H, CH_2_C*H*_3_), 1.40 (d, *J* = 14.7 Hz, 3H, CCH_3_), 1.71–1.85 (m, 2H, C*H*_2_CH_3_), 2.41 (bs, 6H, ArCH_3_), 2.42 (bs, 1H, OH), 7.28–7.32 and 7.87–7.91 (m, 8H, ArH); [M + H]^+^ = 303; HRMS (ESI) *m*/*z*: [M + H]^+^ calculated for C_18_H_24_O_2_P 303.1514; found 303.1511; [M + Na]^+^ calculated for C_18_H_23_O_2_PNa 325.1333; found 325.1335.

#### 3.3.3. Bis(3,5-dimethylphenyl)(α-hydroxy-α-methyl-propyl)phosphine Oxide (**3f**)

Yield: 0.44 g (66%); ^31^P {^1^H} NMR (122 MHz, CDCl_3_) δ 35.5; [M + H]^+^ = 330; HRMS (ESI) *m*/*z*: [M + H]^+^ calculated for C_20_H_28_O_2_P 331.1826; found 331.1827; [M + Na]^+^ calculated for C_20_H_27_O_2_PNa 353.1646; found 353.1647.

### 3.4. General Procedure for the Synthesis of Acylated Diethyl and Dibutyl α-Hydroxy-methyl-propylphosphonates (***4bA***, ***4bB***, ***4bC*** and ***4cA***)

To 1.2 mmol of α-hydroxyphosphonate (**3b**: 0.25 g, **3c**: 0.32 g) and 1.8 mmol (0.25 mL) of triethylamine in toluene (4.0 mL), 3.6 mmol (0.26 mL) of acetyl chloride or 2.4 mmol of other acyl chlorides (propionyl chloride: 0.21 mL or butyryl chloride: 0.25 mL) was added, and the mixture was kept at 60 °C in a sealed tube for 3 days. The precipitated triethylamine hydrochloride was filtered off, and the filtrate was evaporated in vacuum. The crude product so obtained was purified by column chromatography on silica gel, applying dichloromethane–methanol (97:3) as the eluent to give products **4bA**, **4bB**, **4bC** and **4cA** in yields of 48–66% as oils.

#### 3.4.1. Diethyl α-Acetyloxy-α-methyl-propylphosphonate (**4bA**)

Yield: 0.20 g (66%); ^31^P {^1^H} NMR (122 MHz, CDCl_3_) δ_P_ 23.0; ^13^C {^1^H} NMR (75 MHz, CDCl_3_) δ 8.4 (d, *J* = 4.1 Hz, CH_2_*C*H_3_), 16.4 (d, *J* = 5.7 Hz, OCH_2_*C*H_3_), 20.9 (s, C(O)*C*H_3_), 21.9 (s, C*C*H_3_), 28.4 (s, *C*H_2_CH_3_), 62.70 and 62.73 (d, *J* = 7.1 Hz, OCH_2_), 82.3 (d, *J* = 168.7 Hz, CP), 169.6 (d, *J* = 11.5 Hz, C=O); ^1^H NMR (300 MHz, CDCl_3_) δ 1.02 (t, *J* = 7.5 Hz, 3H, CH_2_C*H*_3_), 1.36 (t, *J* = 7.1 Hz, 6H, OCH_2_C*H*_3_), 1.71 (d, *J* = 15.4 Hz, 3H, CCH_3_), 2.08 (s, 3H C(O)CH_3_), 2.08–2.28 (m, 2H, C*H*_2_CH_3_), 4.13–4.27 (m, 4H, OCH_2_); [M + H]^+^ = 253; HRMS (ESI) *m*/*z*: [M + Na]^+^ calculated for C_10_H_21_O_5_PNa 275.1024; found 275.1021.

#### 3.4.2. Diethyl α-Propionyloxy-α-methyl-propylphosphonate (**4bB**)

Yield: 0.32 g (62%); ^31^P {^1^H} NMR (122 MHz, CDCl_3_) δ_P_ 23.2; ^13^C {^1^H} NMR (75 MHz, CDCl_3_) δ 8.4 (d, *J* = 4.1 Hz, CH_2_*C*H_3_), 9.1 (s, CH_2_*C*H_3_), 16.5 (d, *J* = 5.8 Hz, OCH_2_*C*H_3_), 21.0 (s, C*C*H_3_), 28.3 (s, C(O)*C*H_2_), 28.5 (s, *C*H_2_CH_3_), 62.7 (d, *J* = 7.2 Hz, OCH_2_), 82.1 (d, *J* = 168.7 Hz, CP), 173.1 (d, *J* = 11.2 Hz, C=O); ^1^H NMR (300 MHz, CDCl_3_) δ 1.01 (t, *J* = 7.5 Hz, 3H, CH_2_C*H*_3_), 1.14 (t, *J* = 7.6 Hz, 3H, CH_2_C*H*_3_), 1.35 (t, *J* = 7.0 Hz, 6H, OCH_2_C*H*_3_), 1.71 (d, *J* = 15.3 Hz, 3H, CCH_3_), 2.10–2.25 (m, 2H, C*H*_2_CH_3_), 2.31–2.39 (m, 2H, C(O)CH_2_), 4.14–4.25 (m, 4H, OCH_2_); [M + H]^+^ = 267; HRMS (ESI) *m*/*z*: [M + Na]^+^ calculated for C_11_H_23_O_5_PNa 289.1181; found 281.1176.

#### 3.4.3. Diethyl α-Butyryloxyy-α-methyl-propylphosphonate (**4bC**)

Yield: 0.17 g (51%); ^31^P {^1^H} NMR (202 MHz, CDCl_3_) δ_P_ 23.2; ^13^C {^1^H} NMR (126 MHz, CDCl_3_) δ 8.4 (d, *J* = 4.3 Hz, CH_2_*C*H_3_), 13.6 (s, CH_2_*C*H_3_), 16.5 (d, *J* = 5.5 Hz, OCH_2_*C*H_3_), 18.4 (s, *C*H_2_CH_3_), 21.0 (s, C*C*H_3_), 28.5 (s, C(O)CH_2_*C*H_2_), 36.9 (s, C(O)*C*H_2_CH_2_), 62.81 and 62.83 (d, *J* = 7.3 Hz, OCH_2_), 82.0 (d, *J* = 168.6 Hz, CP), 172.35 (d, *J* = 11.2 Hz, C=O); ^1^H NMR (300 MHz, CDCl_3_) δ 0.89 (t, *J* = 7.4 Hz, 3H, CH_2_C*H*_3_), 0.93 (t, *J* = 7.5 Hz, 3H, CH_2_C*H*_3_), 1.27 (t, *J* = 7.0 Hz, 6H, OCH_2_C*H*_3_), 1.54–1.60 (m, 2H, C*H*_2_CH_3_), 1.63 (d, *J* = 15.4 Hz, 3H, CCH_3_), 2.05–2.14 (m, 2H, C(O)CH_2_C*H*_2_), 2.21–2.28 (m, 2H, C(O)C*H*_2_CH_2_), 4.09–4.15 (m, 4H, OCH_2_); [M + H]^+^ = 281; HRMS (ESI) *m*/*z*: [M + Na]^+^ calculated for C_12_H_25_O_5_PNa 303.1337; found 303.1339.

#### 3.4.4. Dibutyl α-Acetyloxy-α-methyl-propylphosphonate (**4cA**)

Yield: 0.18 g (48%); ^31^P {^1^H} NMR (122 MHz, CDCl_3_) δ_P_ 22.9; ^13^C {^1^H} NMR (75 MHz, CDCl_3_) δ 8.5 (d, *J* = 4.1 Hz, CH_2_*C*H_3_), 13.6 (s, CH_2_*C*H_3_), 18.8 (s, *C*H_2_CH_3_), 21.0 (s, C(O)*C*H_3_), 22.0 (s, C*C*H_3_), 28.5 (s, *C*H_2_CH_3_), 32.7 (d, *J* = 5.8 Hz, OCH_2_*C*H_2_), 66.5 (d, *J* = 7.3 Hz, OCH_2_), 82.6 (d, *J* = 169.0 Hz, CP), 169.7 (d, *J* = 11.8 Hz, C=O); ^1^H NMR (300 MHz, CDCl_3_) δ 0.95 (t, *J* = 7.5 Hz, 6H, CH_2_CH_3_), 1.01 (t, *J* = 7.5 Hz, 3H, CH_2_C*H*_3_), 1.36–1.48 (m, 4H, C*H*_2_CH_3_), 1.70 (d, *J* = 15.6 Hz, 3H, CCH_3_) overlapped with 1.63–1.70 (m, 4H, OCH_2_C*H*_2_), 2.07 (s, 3H, C(O)CH_3_) overlapped with 2.07–2.27 (m, 2H, C*H*_2_CH_3_), 4.09–4.16 (m, 4H, OCH_2_); [M + H]^+^ = 309; HRMS (ESI) *m*/*z*: [M + Na]^+^ calculated for C_14_H_29_O_5_PNa 331.1650; found 331.1644.

### 3.5. General Procedure for the Synthesis of Phosphinoylated Diethyl α-Hydroxy-ethylphosphonate and Diphenyl α-Hydroxy-ethylphosphine Oxide (***6a*** and ***6b***)

To 1.0 mmol of α-hydroxyphosphonate (**1b**: 0.18 g, **1d**: 0.24 g) and 1.5 mmol (0.21 mL) of triethylamine in toluene (1.0 mL), 1.8 mmol (0.33 mL) of dipenylphosphinic chloride was added, and the mixture was kept at 25 °C (in case of **1b**) or 60 °C (in case of **1d**) for 2 days. The precipitated triethylamine hydrochloride was filtered off, and the filtrate was evaporated in vacuum. The crude product so obtained was purified by column chromatography on silica gel, applying dichloromethane–methanol (97:3) as the eluent to give products **6a** and **6b** as oils.

#### 3.5.1. Diethyl α-Diphenylphosphinoyloxy-ethylphosphonate (**6a**)

Yield: 0.31 g (81%); ^31^P {^1^H} NMR (202 MHz, CDCl_3_) δ_P1_ 20.8 and δ_P2_ 33.8 (d, *J* = 28.7 Hz); ^13^C {^1^H} NMR (126 MHz, CDCl_3_) δ 16.3 and 16.4 (d, *J* = 5.9 Hz, OCH_2_*C*H_3_), 17.3 (bs, C*C*H_3_), 62.8 and 62.9 (d, *J* = 6.8 Hz, OCH_2_), 66.9 (dd, *J*_1_ = 174.7 Hz, *J*_2_ = 7.2 Hz, CH), 128.47 and 128.49 (d, *J* = 13.3 Hz, C_β_), 130.9 and 132.0 (d, *J* = 133.8 Hz, C_α_), 131.5 and 131.9 (d, *J* = 10.6 Hz, C_γ_), 132.3 and 132.4 (d, *J* = 2.8 Hz, C_δ_); ^1^H NMR (500 MHz, CDCl_3_) δ 1.30 and 1.31 (t, *J* = 7.1 Hz, 6H, OCH_2_C*H*_3_), 1.58 (dd, *J*_1_ = 16.8 Hz, *J*_2_ = 7.1Hz, 3H, CCH_3_), 4.09–4.22 (m, 4H, OCH_2_), 4.77–4.85 (m, 1H, CH), 7.46–7.50, 7.54–7.57 and 7.84–7.91 (m, 10H, ArH); [M + H]^+^ = 383; HRMS (ESI) *m*/*z*: [M + H]^+^ calculated for C_18_H_25_O_5_P_2_ 383.1177; found 383.1177; [M + Na]^+^ calculated for C_18_H_24_O_5_P_2_Na 405.0997; found 405.0999.

#### 3.5.2. Diphenyl α-Diphenylphosphinoyloxy-ethylphosphine oxide (**6b**)

Yield: 0.23 g (52%); ^31^P {^1^H} NMR (122 MHz, CDCl_3_) δ_P1_ 31.5 and δ_P2_ 34.0 (d, *J* = 25.5 Hz); ^13^C {^1^H} NMR (126 MHz, CDCl_3_) δ 16.0 (bs, C*C*H_3_), 70.0 (dd, *J*_1_ = 89.9 Hz, *J*_2_ = 7.9 Hz, CH), 128.34 and 128.5 (d, *J* = 13.2 Hz, C_β_), 128.6 and 128.8 (d, *J* = 11.8 Hz, C_β’_), 128.29 and 130.40 (d, *J* = 98.9 Hz, C_α’_), 130.7 and 131.8 (d, *J* = 136.3 Hz, C_α_), 131.3 and 131.60 (d, *J* = 10.5 Hz, C_γ_), 131.57 and 132.2 (d, *J* = 8.7 Hz, C_γ’_), 132.1 and 132.4 (d, *J* = 2.8 Hz, C_δ_), 132.34 and 132.36 (d, *J* = 2.5 Hz, C_δ’_); ^1^H NMR (500 MHz, CDCl_3_) δ 1.45 (dd, *J*_1_ = 14.6 Hz, *J*_2_ = 7.0 Hz, 3H, CCH_3_), 5.34–5.41 (m, 1H, CH), 7.15–7.18, 7.19–7.23, 7.32–7.41, 7.43–7.50, 7.59–7.64, 7.68–7.73 and 7.86–7.90 (m, 20H, ArH); [M + H]^+^ = 447; HRMS (ESI) *m*/*z*: [M + H]^+^ calculated for C_26_H_24_O_3_P_2_ 447.1279; found 447.1285; [M + Na]^+^ calculated for C_26_H_24_O_3_P_2_Na 469.1098; found 469.1100.

### 3.6. Synthesis of Diethyl 1-(Diethylphosphonoylethyl)phosphate (***6c***)

To 1.0 mmol (0.18 g) of α-hydroxyphosphonate **1b** and 1.5 mmol (0.21 mL) of triethylamine in toluene (1.0 mL), 5.0 mmol (0.72 mL) of diethyl chlorophosphate was added, and the mixture was kept at 25 °C for 2 days. The precipitated triethylamine hydrochloride was filtered off, and the filtrate was evaporated in vacuum. The crude product so obtained was purified by column chromatography on silica gel, applying dichloromethane–methanol (97:3) as the eluent to give product **6c** in a yield of 88% (0.28 g) as oil.

^31^P {^1^H} NMR (202 MHz, CDCl_3_) δ_P1_−1.2 and δ_P2_ 20.3 (d, *J* = 32.4 Hz); δ_P1_−1.2 and δ_P2_ 20.3 (d, *J* = 32.0 Hz) [62]; [M + H]^+^ = 319; HRMS (ESI) *m*/*z*: [M + H]^+^ calculated for C_10_H_25_O_7_P_2_ 319.1076; found 319.1072; [M + Na]^+^ calculated for C_10_H_24_O_7_P_2_Na 341.0895; found 341.0894.

### 3.7. General Procedure for the Synthesis of Phosphinoylated Diethyl α-Hydroxy-α-methyl-ethylphosphonate and α-Hydroxy-α-methyl-ethylphosphine Oxide (***8a*** and ***8b***)

To 1.0 mmol of α-hydroxyphosphonate (**2b**: 0.20 g, **3b**: 0.21 g) and 1.5 mmol (0.21 mL) of triethylamine in toluene (1.0 mL), 1.5 mmol (0.28 mL) of dipenylphosphinous chloride was added, and the mixture was kept at 60 °C in a sealed tube under N_2_ atmosphere for 1 day. The precipitated triethylamine hydrochloride was filtered off, and the filtrate was evaporated in vacuum. To the product so obtained, 5 mL of dichloromethane was added, then 3.0 mmol (0.31 mL) of 30% H_2_O_2_ solution was added dropwise at 0 °C. The mixture was stirred for 1 h, then extracted with 2×5 mL of water. The phases were separated, and after drying (Na_2_SO_4_), the organic phase was evaporated in vacuum. The crude product so obtained was purified by column chromatography on silica gel, applying dichloromethane–methanol (97:3) as the eluent to give products **8a** and **8b** as oils.

#### 3.7.1. Diethyl α-Diphenylphosphinoyloxy-methyl-ethylphosphonate (**8a**)

Yield: 0.25 g (63%); ^31^P {^1^H} NMR (202 MHz, CDCl_3_) δ_P1_ 22.2 and δ_P2_ 29.9 (d, *J* = 35.3 Hz); ^13^C {^1^H} NMR (75 MHz, CDCl_3_) δ 16.5 (d, *J* = 5.6 Hz, OCH_2_*C*H_3_), 24.6 (d, *J* = 2.2 Hz, C*C*H_3_), 63.1 (d, *J* = 7.1 Hz, OCH_2_), 81.5 (dd, *J*_1_ = 177.8 Hz, *J*_2_ = 9.1 Hz, CP), 128.4 (d, *J* = 13.3 Hz, C_β_), 131.3 (d, *J* = 10.5 Hz, C_γ_), 131.9 (d, *J* = 2.9 Hz, C_δ_), 133.6 (d, *J* = 138.1 Hz, C_α_); ^1^H NMR (500 MHz, CDCl_3_) δ 1.33 (t, *J* = 7.1 Hz, 6H, OCH_2_C*H*_3_), 1.76 (d, *J* = 15.7 Hz, 3H, CCH_3_), 4.14–4.26 (m, 4H, OCH_2_), 7.43–7.47, 7.49–7.54 and 7.84–7.88 (m, 10H, ArH); [M + H]^+^ = 397; HRMS (ESI) *m*/*z*: [M + Na]^+^ calculated for C_19_H_26_O_5_P_2_Na 419.1153; found 419.1156.

#### 3.7.2. Diethyl α-Diphenylphosphinoyloxy-methyl-propylphosphonate (**8b**)

Yield: 0.27 g (65%); ^31^P {^1^H} NMR (122 MHz, CDCl_3_) δ_P1_ 22.1 and δ_P2_ 29.3 (d, *J* = 31.5 Hz); ^13^C {^1^H} NMR (75 MHz, CDCl_3_) δ 8.9 (d, *J* = 4.3 Hz, CH_2_*C*H_3_), 16.5 (d, *J* = 5.8 Hz, OCH_2_*C*H_3_), 22.1 (bs, C*C*H_3_), 31.7 (bs, *C*H_2_CH_3_), 62.8 and 62.9 (d, *J* = 7.3 Hz, OCH_2_), 85.2 (dd, *J*_1_ = 173.6 Hz, *J*_2_ = 10.0 Hz, CP), 128.30 and 128.33 (d, *J* = 13.4 Hz, C_β_), 131.2 and 131.7 (d, *J* = 10.6 Hz, C_γ_), 131.76 and 131.83 (d, *J* = 3.6 Hz, C_δ_), 133.8 and 134.0 (d, *J* = 138.9 Hz, C_α_); ^1^H NMR (300 MHz, CDCl_3_) δ 1.07 (t, *J* = 7.5 Hz, 3H, CH_2_C*H*_3_), 1.30 and 1.31 (t, *J* = 6.9 Hz, 6H, OCH_2_C*H*_3_), 1.70 (d, *J* = 15.9 Hz, 3H, CCH_3_), 2.11–2.24 (m, 2H, C*H*_2_CH_3_), 4.09–4.23 (m, 4H, OCH_2_), 7.39–7.54 and 7.79–7.93 (m, 10H, ArH); [M + H]^+^ = 411; HRMS (ESI) *m*/*z* [M + Na]^+^ calculated for C_20_H_28_O_5_P_2_Na 433.1310; found 433.1305.

### 3.8. General Procedure for the Synthesis of Thiophosphinoylated Diethyl α-Hydroxy-ethylphosphonate and α-Hydroxy-α-ethylphosphine Oxide (***9a*** and ***9b***)

To 1.0 mmol of α-hydroxyphosphonate (**1b**: 0.18 g, **1d**: 0.24 g) and 1.5 mmol (0.21 mL) of triethylamine in toluene (2.0 mL), 1.5 mmol (0.28 mL) of dipenylphosphinous chloride and 2 mmol (64 mg) of S_8_ were added, and the mixture was kept at 60 °C in a sealed tube under N_2_ atmosphere for 1 day. The precipitated triethylamine hydrochloride was filtered off, and the filtrate was evaporated in vacuum. The crude product so obtained was purified by column chromatography on silica gel, applying dichloromethane–methanol (97:3) as the eluent to give products **9a** and **9b** as oils.

#### 3.8.1. Diethyl α-Diphenyl-thiophosphinoyloxy-ethylphosphonate (**9a**)

Yield: 0.37 g (94%); ^31^P {^1^H} NMR (202 MHz, CDCl_3_) δ_P1_ 21.4 and δ_P2_ 84.5 (d, *J* = 31.8 Hz); ^13^C {^1^H} NMR (126 MHz, CDCl_3_) δ 16.3 and 16.4 (d, *J* = 5.8 Hz, OCH_2_*C*H_3_), 16.9 (bs, C*C*H_3_), 62.3 and 62.8 (d, *J* = 6.8 Hz, OCH_2_), 66.5 (dd, *J*_1_ = 173.6 Hz, *J*_2_ = 7.1 Hz, CH), 128.3 and 128.4 (d, *J* = 13.8 Hz, C_β_), 131.09 and 131.13 (d, *J* = 11.7 Hz, C_γ_), 131.8 and 131.9 (d, *J* = 3.1 Hz, C_δ_), 134.8 and 135.4 (d, *J* = 111.2 Hz, C_α_); ^1^H NMR (500 MHz, CDCl_3_) δ 1.27 (t, *J* = 7.0 Hz, 6H, OCH_2_C*H*_3_), 1.47 (dd, *J*_1_ = 16.8 Hz, *J*_2_ = 7.0 Hz, 3H, CCH_3_), 4.02–4.18 (m, 4H, OCH_2_), 5.15–5.24 (m, 1H, CH), 7.42–7.54 and 7.88–7.92 (m, 10H, ArH); [M + H]^+^ = 398; HRMS (ESI) *m*/*z*: [M + H]^+^ calculated for C_18_H_25_O_4_P_2_S 399.0949; found 399.0946; [M + Na]^+^ calculated for C_18_H_24_O_4_P_2_SNa 421.0768; found 421.0769.

#### 3.8.2. Diphenyl α-Diphenyl-thiophosphinoyloxy-ethylphosphine Oxide (**9b**)

Yield: 0.35 g (76%); ^31^P {^1^H} NMR (122 MHz, CDCl_3_) δ_P1_ 32.1 and δ_P2_ 84.8 (d, *J* = 29.5 Hz); ^13^C {^1^H} NMR (126 MHz, CDCl_3_-MeOH 95:5) δ 15.5 (bs, C*C*H_3_), 69.5 (dd, *J*_1_ = 89.7 Hz, *J*_2_ = 8.1 Hz, CH), 128.1 and 128.4 (d, *J* = 13.7 Hz, C_β_), 128.5 and 128.8 (d, *J* = 11.8 Hz, C_β’_), 128.7 and 130.55 (d, *J* = 99.0 Hz, C_α’_), 130.3 and 131.4 (d, *J* = 11.7 Hz, C_γ_), 131.46 and 132.13 (d, *J* = 9.0 Hz, C_γ’_), 131.48 and 132.13 (d, *J* = 3.1 Hz, C_δ_), 132.2 and 132.3 (d, *J* = 2.8 Hz, C_δ’_), 134.6 and 135.0 (d, *J* = 110.8 Hz, C_α_); ^1^H NMR (500 MHz, CDCl_3_) δ 1.37 (dd, *J*_1_ = 14.3 Hz, *J*_2_ = 6.9 Hz, 3H, CCH_3_), 5.87–5.94 (m, 1H, CH), 7.16–7.20, 7.23–7.27, 7.34–7.37, 7.39–7.45, 7.49–7.56, 7.78–7.86 and 7.92–7.98 (m, 20H, ArH); HRMS (ESI) *m*/*z*: [M + Na]^+^ calculated for C_26_H_24_O_2_P_2_SNa 485.0870; found 485.0875; [M + H]^+^ = 463.

### 3.9. General Procedure for the Synthesis of Thiophosphinoylated Diethyl α-Hydroxy-methyl-ethylphosphonate and α-Hydroxy-α-methyl-propylphosphonate (***10*** and ***11***)

To 1.0 mmol of α-hydroxyphosphonate (**2b**: 0.18 g, **3b**: 0.24g) and 1.5 mmol (0.21 mL) of triethylamine in toluene (2.0 mL), 1.5 mmol (0.28 mL) of dipenylphosphinous chloride was added, and the mixture was kept at 25 °C in a sealed tube under N_2_ atmosphere for 1h. Then, 2 mmol (64 mg) of S_8_ was added, and the reaction mixture was kept at 80 °C for 3 days under N_2_ atmosphere. The precipitated triethylamine hydrochloride was filtered off, and the filtrate was evaporated in vacuum. The crude product so obtained was purified by column chromatography on silica gel, applying dichloromethane–methanol (97:3) as the eluent to give products **10** and **11** as oils.

#### 3.9.1. Diethyl α-Diphenyl-thiophosphinoyloxy-methyl-ethylphosphonate (**10**)

^31^P {^1^H} NMR (202 MHz, CDCl_3_) δ_P1_ 28.9 and δ_P2_ 80.0 (d, *J* = 40.1 Hz); [M + H]^+^ = 413.

#### 3.9.2. Diethyl α-Diphenyl-thiophosphinoyloxy-α-methyl-propylphosphonate (**11**)

^31^P {^1^H} NMR (202 MHz, CDCl_3_) δ_P1_ 29.0 and δ_P2_ 80.3 (d, *J* = 40.2 Hz); [M + H]^+^ = 427; HRMS (ESI) *m*/*z*: [M + Na]^+^ calculated for C_20_H_28_O_4_P_2_SNa 449.1081; found 449.1103.

### 3.10. General Procedure for the Synthesis of Mesylated Dimethyl and Diethyl α-Hydroxy-methyl-ethylphosphonates (***12*** and ***12b***)

A total of 1.2 mmol of α-hydroxyphosphonate (**2a**: 0.20 g; **2b**: 0.24 g), 1.8 mmol (0.14 mL) of methanesulfonyl chloride and 1.8 mmol (0.25 mL) of triethylamine in 5 mL of toluene were mixed at room temperature for 24 h. The precipitated triethylamine hydrochloride salt was filtered off, and the filtrate was evaporated under vacuum. The crude product so obtained was purified by column chromatography (using DCM–MeOH 95:5 as the eluent on silica gel).

#### 3.10.1. Dimethyl α-Methanesulfonyloxy-methyl-ethylphosphonate (**12a**)

Yield: 0.24 g (81%); ^31^P {^1^H} NMR (122 MHz, CDCl_3_) δ_P_ 21.9; ^13^C {^1^H} NMR (75 MHz, CDCl_3_) δ 23.0 (s, C*C*H_3_), 41.0 (s, SCH_3_), 54.1 (d, *J* = 7.1 Hz, OCH_3_), 87.4 (d, *J* = 175.9 Hz, CP); ^1^H NMR (300 MHz, CDCl_3_) δ 1.91 (d, *J* = 15.6 Hz, 6H, CCH_3_), 3.11 (s, 3H, SCH_3_), 3.88 (d, *J* = 10.4 Hz, 6H, OCH_3_); [M + H]^+^ = 247; HRMS (ESI) *m*/*z*: [M + Na]^+^ calculated for C_6_H_15_O_6_PSNa 269.0225; found 269.0225.

#### 3.10.2. Diethyl α-Methanesulfonyloxy-methyl-ethylphosphonate (**12b**)

Yield: 0.26 g (79%); ^31^P {^1^H} NMR (202 MHz, CDCl_3_) δ_P_ 19.5; ^13^C {^1^H} NMR (75 MHz, CDCl_3_) δ 16.4 (d, *J* = 5.5 Hz, CH_2_CH_3_), 23.0 (s, C*C*H_3_), 41.1 (s, SCH_3_), 63.6 (d, *J* = 7.1 Hz, OCH_2_), 87.4 (d, *J* = 175.9 Hz, CP); ^1^H NMR (500 MHz, CDCl_3_) δ 1.31 (t, *J* = 7.0 Hz, 6H, CH_2_C*H*_3_)1.91 (d, *J* = 15.5 Hz, 6H, CCH_3_), 3.10 (s, 3H, SCH_3_), 4.21–4.27 (m, 4H, OCH_2_); [M + H]^+^ = 275; HRMS (ESI) *m*/*z*: [M + Na]^+^ calculated for C_8_H_19_O_6_PSNa 297.0538; found 298.0533.

### 3.11. Bioactivity Experimental

#### 3.11.1. Cell Culturing

In the present study, two human cell lines—A2058 melanoma and U266 myeloma—were used to evaluate the effects of α-hydroxy-alkylphosphonates and their derivatives on cell viability. Both cell lines were obtained from the European Collection of Authenticated Cell Cultures (A2058: 91100402 and U266: 85051003, ECACC, Salisbury, UK) and cultured in RPMI 1640 medium (Sigma Ltd., St. Louis, MO, USA). The medium was supplemented with 10% fetal bovine serum (Biosera, Kansas, MO, USA), 1% L-glutamine (Invitrogen Corporation, New York, NY, USA), and 1% penicillin/streptomycin (Invitrogen Corporation, New York, NY, USA).

#### 3.11.2. Cell Viability Assays

Test compounds were dissolved in dimethyl sulfoxide (DMSO; AppliChem GmbH, Darmstadt, Germany) to prepare stock solutions at a concentration of 10^−1^ M. During the experiments, the final DMSO concentration did not exceed 1% (*v*/*v*) to avoid any effect of the solvent on cell viability. Stock solutions were stored at −80 °C, and working solutions were freshly prepared prior to each experiment. Cell viability of A2058 and U266 cells was assessed using the CellTiter-Glo Luminescent Cell Viability Assay (Promega, Madison, WI, USA), following the protocol described in our previous publication [63]. All experiments were performed in triplicate within a single experimental run. Results were normalized to the medium control using OriginPro 8 software (OriginLab Corporation, Northampton, MA, USA) and are presented as mean ± standard deviation (SD).

#### 3.11.3. Statistical Analysis

All experimental data are presented as mean values ± standard deviation (SD). Measurements were normalized relative to the DMSO-treated control group. Statistical significance between groups was evaluated using one-way analysis of variance (ANOVA) followed by Fisher’s least significant difference (LSD) post hoc test. When significance indicators are displayed above the bars, they refer to comparisons with the DMSO control group. Statistical significance was defined as follows: * *p* < 0.05; ** *p* < 0.01; *** *p* < 0.001.

## 4. Conclusions

Acetaldehyde–, acetone– and ethyl methyl ketone–dialkyl phosphite and secondary phosphine oxide adducts were synthesized by the Pudovik reaction. The hydroxyphosphonates and hydroxyphosphine oxides so obtained were acylated and phosphinoylated/phosphorylated. Due to steric hindrance during the preparation of the acetone– and ethyl methyl ketone–diethyl phosphite adducts, instead of phosphinyl chloride, phosphinous chloride was applied, followed by oxidation. The latter protocol was applied to the thiophosphinoylation of the acetaldehyde–>P(O)H reagent adducts, but starting from the corresponding acetone– and ethyl methyl ketone–diethyl phosphite adducts, the thiophosphinoylation was rather reluctant. A part of the α-hydroxyphosphonates could be successfully methanesulfonylated. The new derivatives prepared were tested on myeloma and melanoma cells.

Our results demonstrate that structural modification by phosphinoylation, particularly by diphenylthiophosphinoylation, is critical for achieving significant antiproliferative activity among the α-hydroxyphosphonate and α-hydroxyphosphine oxide derivatives, with the strongest effects observed on U266 myeloma cells. The enhanced activity associated with the Ph_2_P(S) moiety suggests that increased lipophilicity and improved cellular uptake are the key contributors to the observed biological effects. In contrast, acylation or methanesulfonylation failed to improve efficacy, highlighting the importance of thiophosphinoyl moiety in optimizing cytotoxic potency.

## Data Availability

The data presented in this study are available on request from the corresponding author.

## Supplementary Materials

The following Supporting Information can be downloaded at: https://www.mdpi.com/article/10.3390/ph19030396/s1, ^31^P, ^13^C and ^1^H NMR spectra for compounds **3a–e**, **4bA–C**, **4cA**, **6a**, **6b**, **8a**, **8b**, **9a**, **9b**, **12a** and **12b** synthesized.
