# Supplementary material for: The Synthesis of α-Hydroxy-Alkylphosphonate Derivatives and Evaluation of Their Cytotoxic Activity
Source: Pharmaceuticals (Basel). 2026 Feb 28;19(3):396. doi: 10.3390/ph19030396 (PMC13029092; doi:10.3390/ph19030396)

Supplementary Materials

The Synthesis of α-Hydroxy-Alkylphosphonate Derivatives and Evaluation of Their Cytotoxic Activity

Zsuzsanna Szalai [1], Regina Facskó [1], Ágnes Gömöry [2], László Drahos [2], Szilárd Tekula [3], Angéla Takács [3], László Kőhidai [3] and György Keglevich [1,*]

1 Department of Organic Chemistry and Technology, Faculty of Chemical Technology and Biotechnology, Budapest University of Technology and Economics, Műegyetem rkp. 3., 1111 Budapest, Hungary; szalai.zsuzsanna@edu.bme.hu (Z.S.); facskor@edu.bme.hu (R.F.)

2 MS Proteomics Research Group, Research Centre for Natural Sciences, 1117 Budapest, Hungary; gomory.agnes@ttk.hu (Á.G.); drahos.laszlo@ttk.hu (L.D.)

3 Department of Genetics, Cell- and Immunobiology, Semmelweis University, Nagyvárad tér 4., 1089 Budapest, Hungary; tekula.szilard@stud.semmelweis.hu (S.T.); takacs.angela@semmelweis.hu (A.T.); kohidai.laszlo@semmelweis.hu (L.K.)

* Correspondence: keglevich.gyorgy@vbk.bme.hu; Tel.: +36-1-463-1111 (ext. 5883)

Table of Contents

^{31}P, ^{13}C and ^{1}H NMR spectra for compounds **3a–e**, **4bA–C**, **4cA**, **6a**, **6b**, **8a**, **8b**, **9a**, **9b**, **12a** and **12b** synthesized.

Spectra for the compounds 3a-e, 4bA-C, 6a-c, 8a, 8b, 9a, 9b, 12a and 12b synthesized

^{31}P {^{1}H} NMR (122 MHz, CDCl$_3$) spectra for 3a

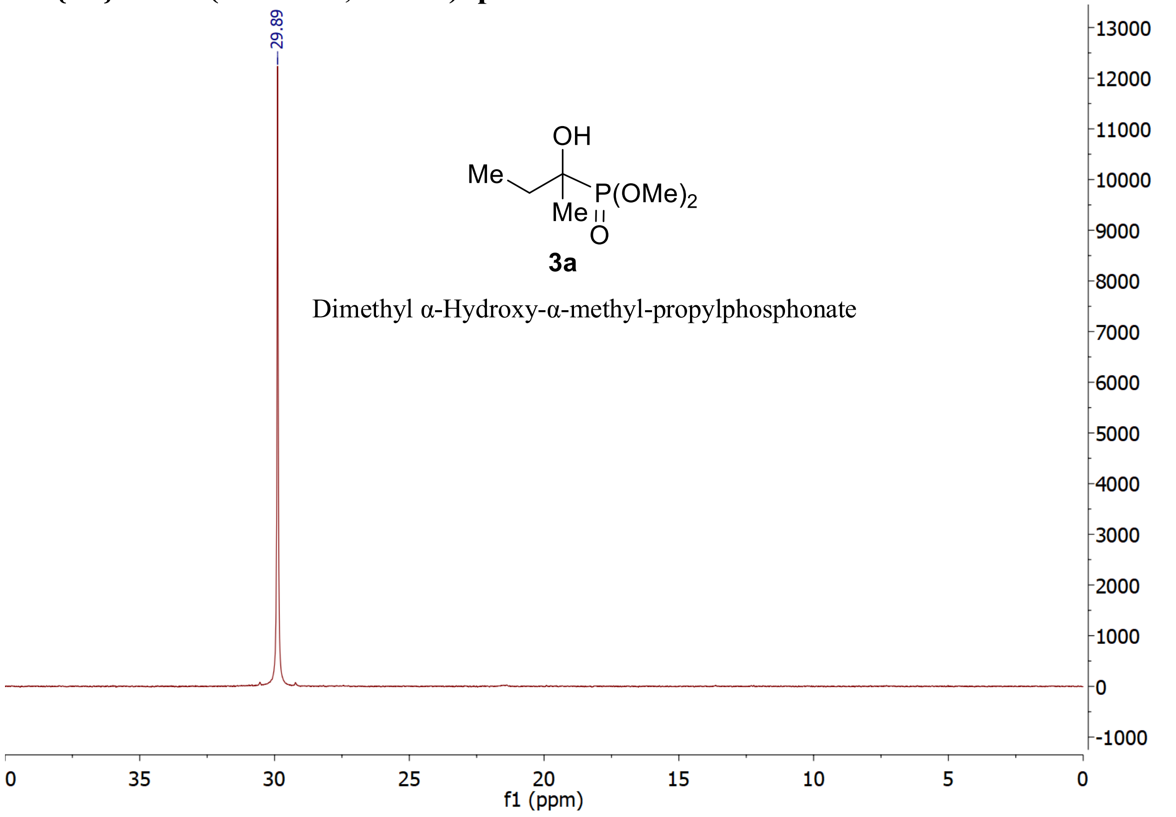

^{31}P {^{1}H} NMR (202 MHz, CDCl$_3$) spectra for 3b

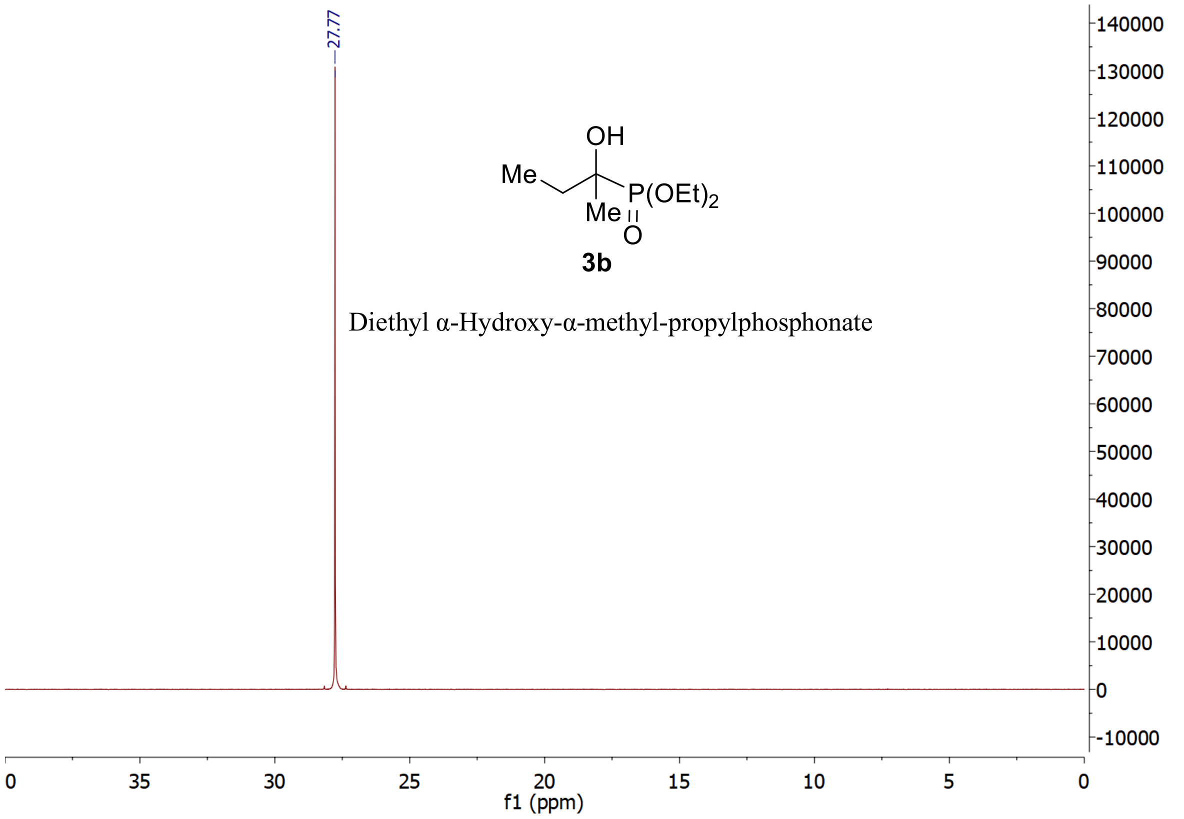

$^{31}P\ \{^1H\}$ NMR (202 MHz, $CDCl_3$) spectra for 3c

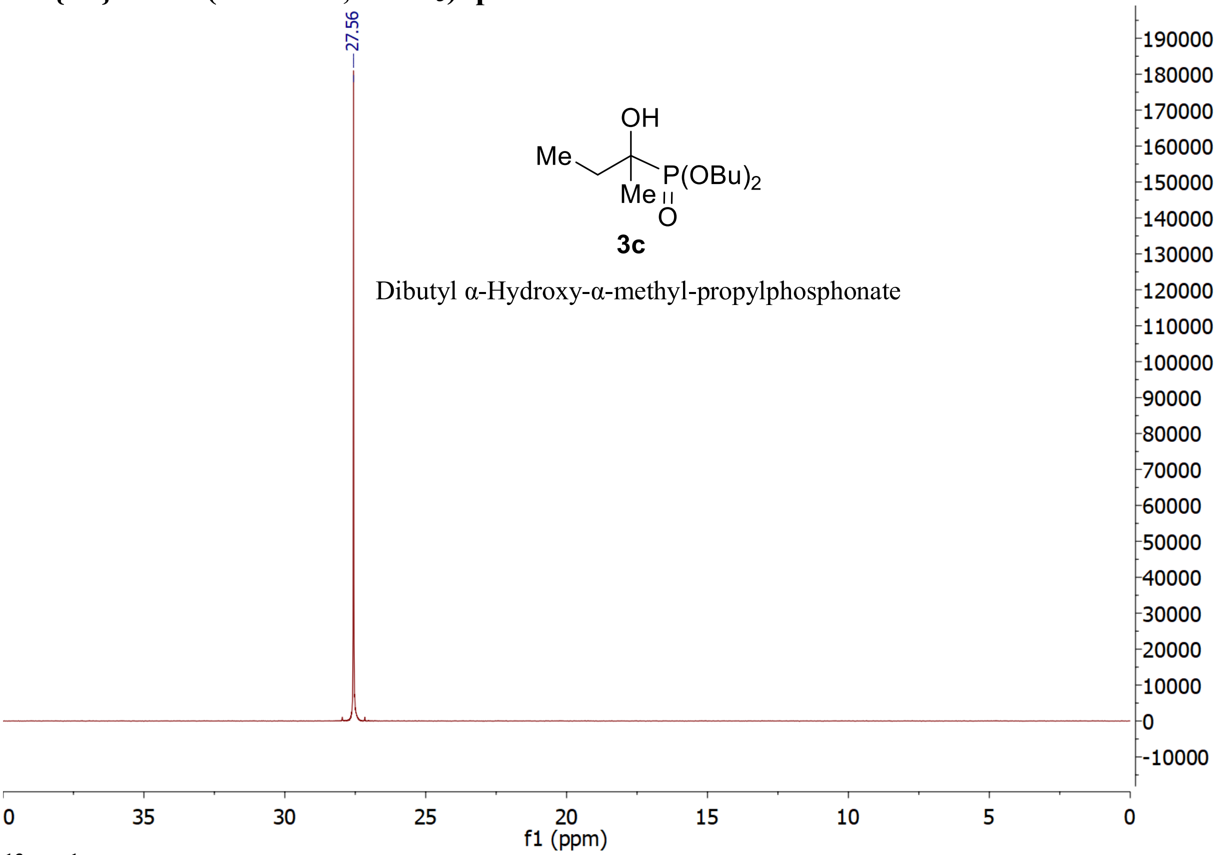

$^{13}C\ \{^1H\}$ NMR (126 MHz, $CDCl_3$) spectra for 3c

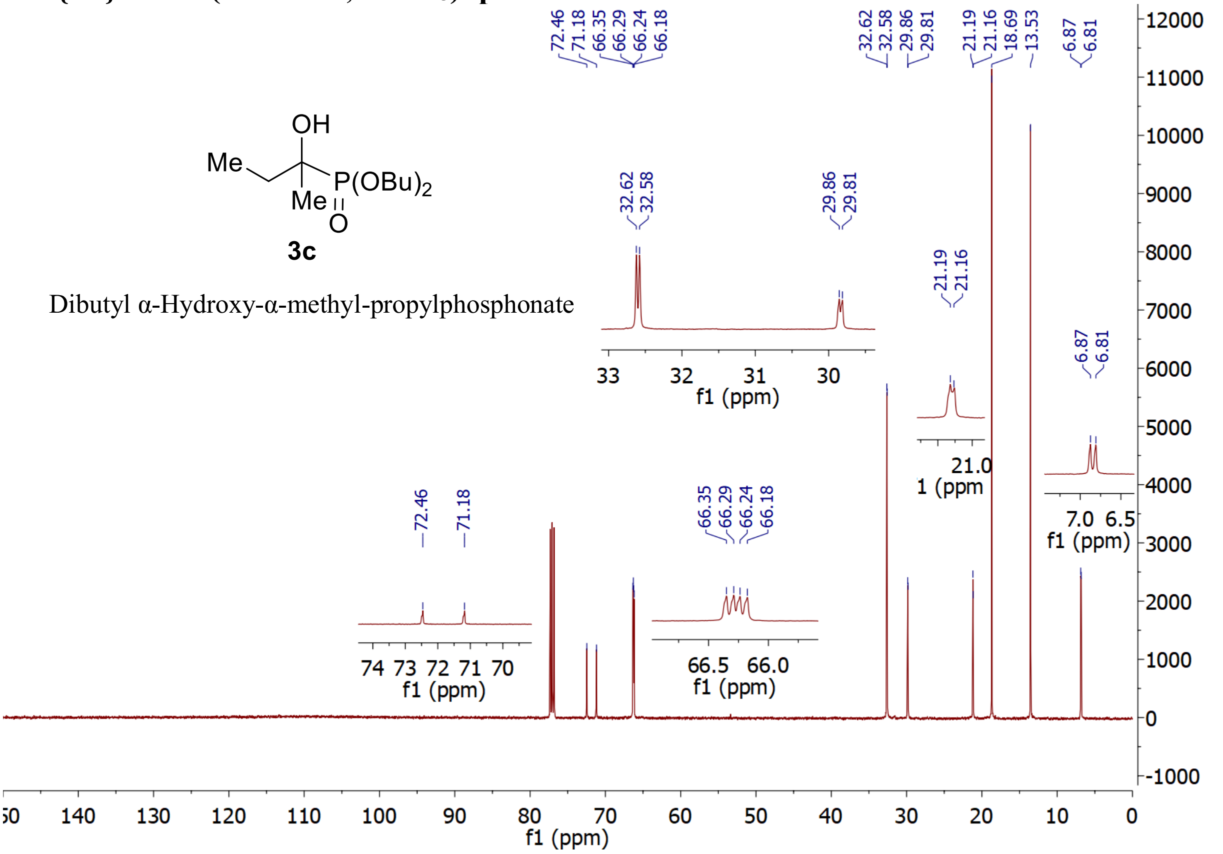

^{1}H NMR (500 MHz, $CDCl_3$) spectra for 3c

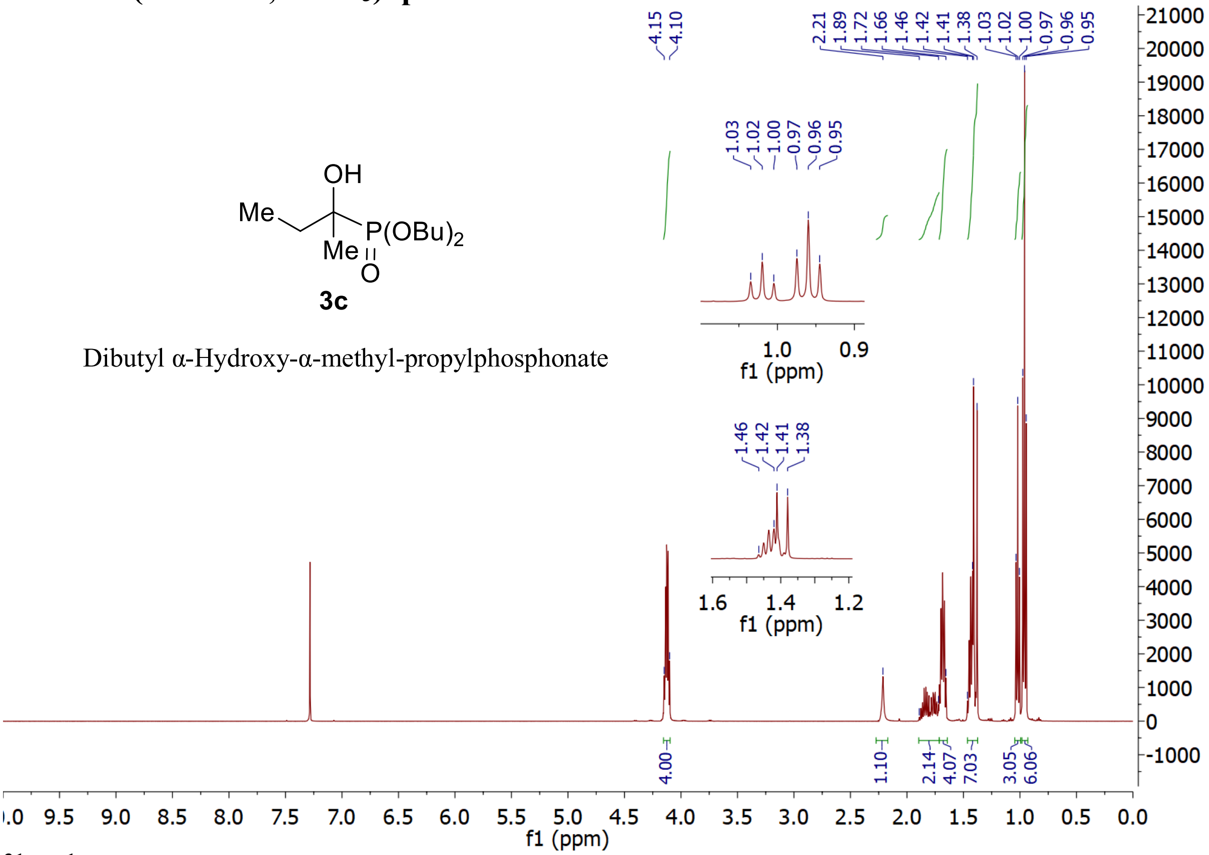

^{31}P {^{1}H} NMR (202 MHz, $CDCl_3$) spectra for 3d

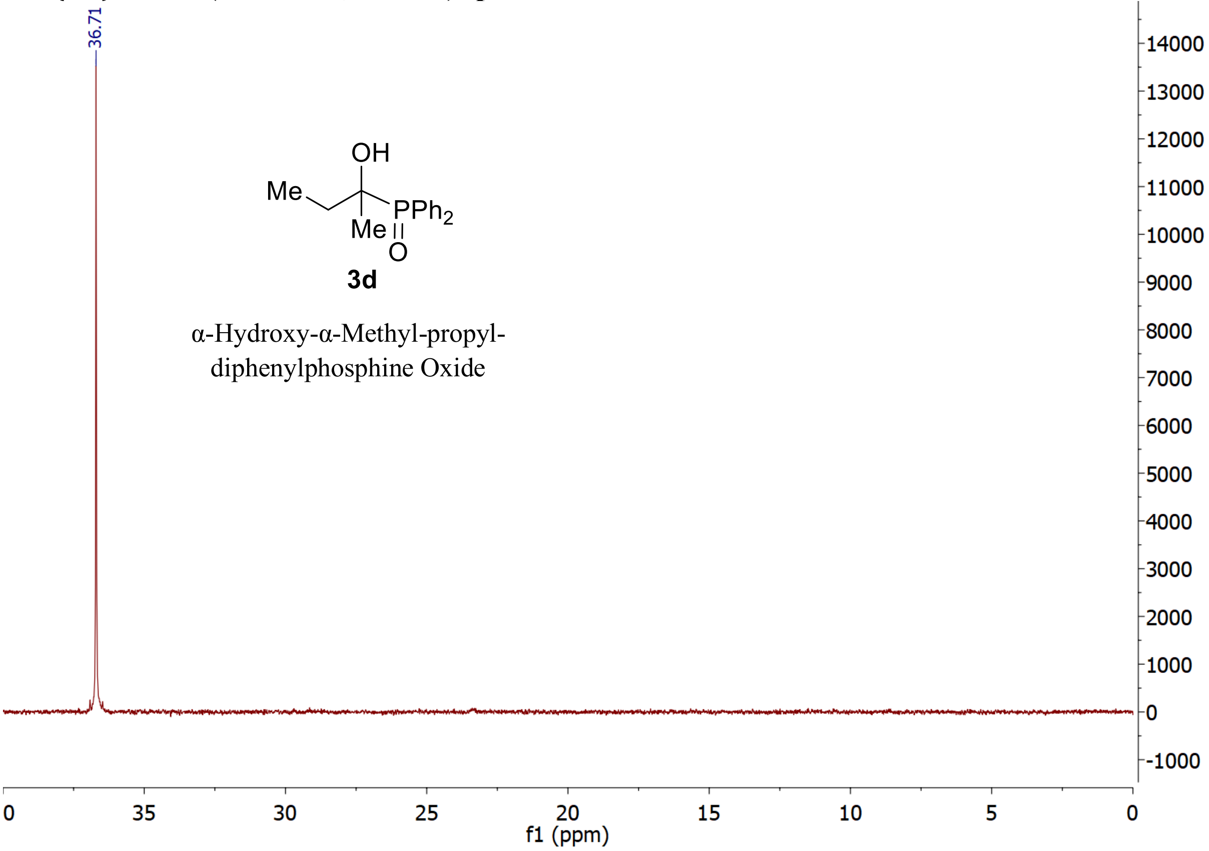

$^{31}P\ \{^{1}H\}$ NMR (202 MHz, $CDCl_3$) spectra for 3e

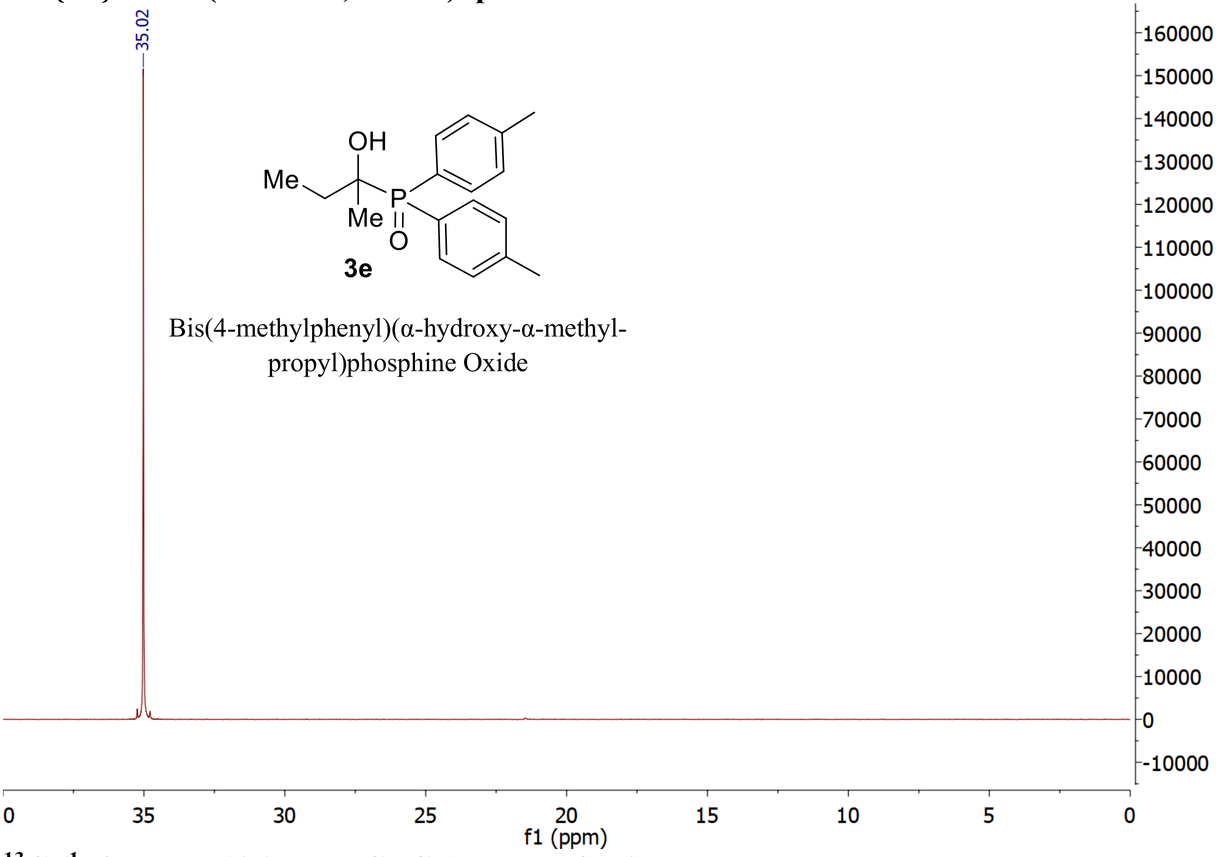

$^{13}C\ \{^{1}H\}$ NMR (126 MHz, $CDCl_3$) spectra for 3e

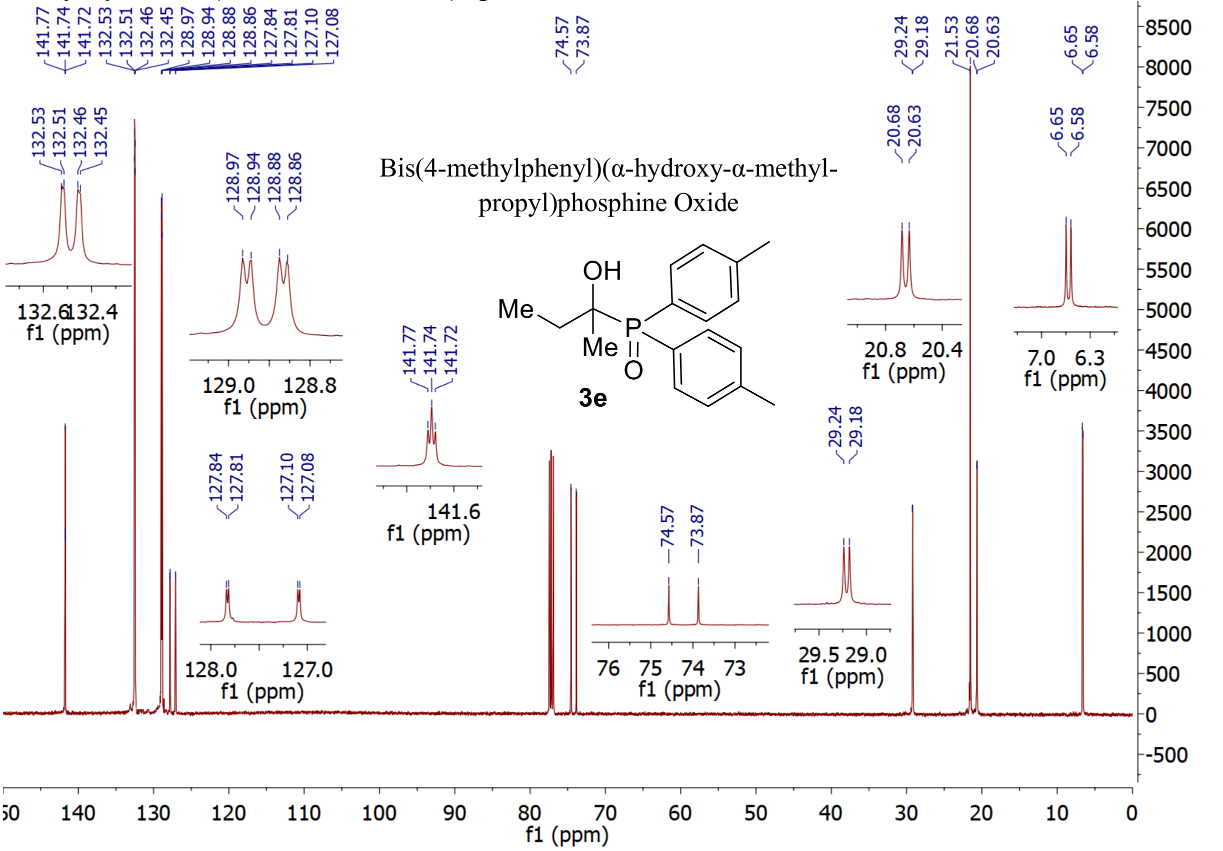

^{1}H NMR (500 MHz, $CDCl_3$) spectra for 3e

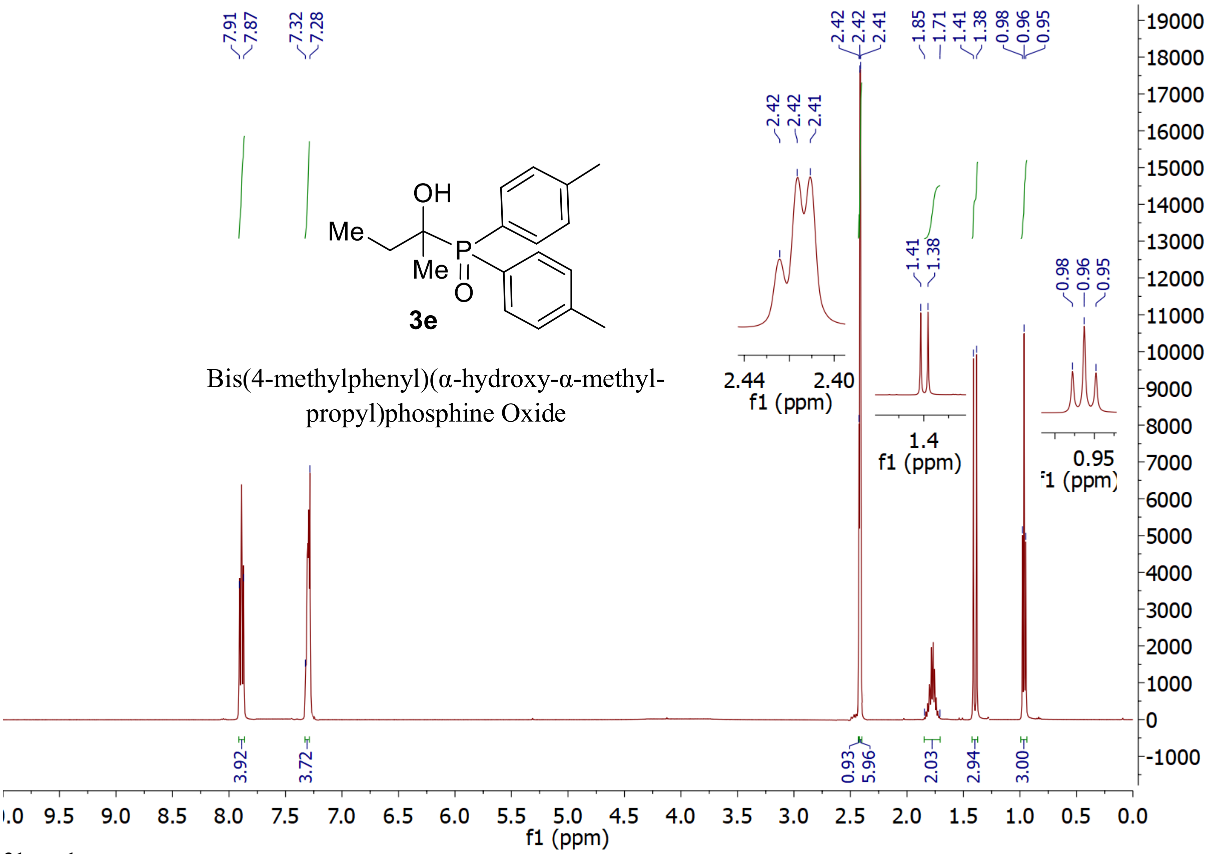

^{31}P {^{1}H} NMR (122 MHz, $CDCl_3$) spectra for 4bA

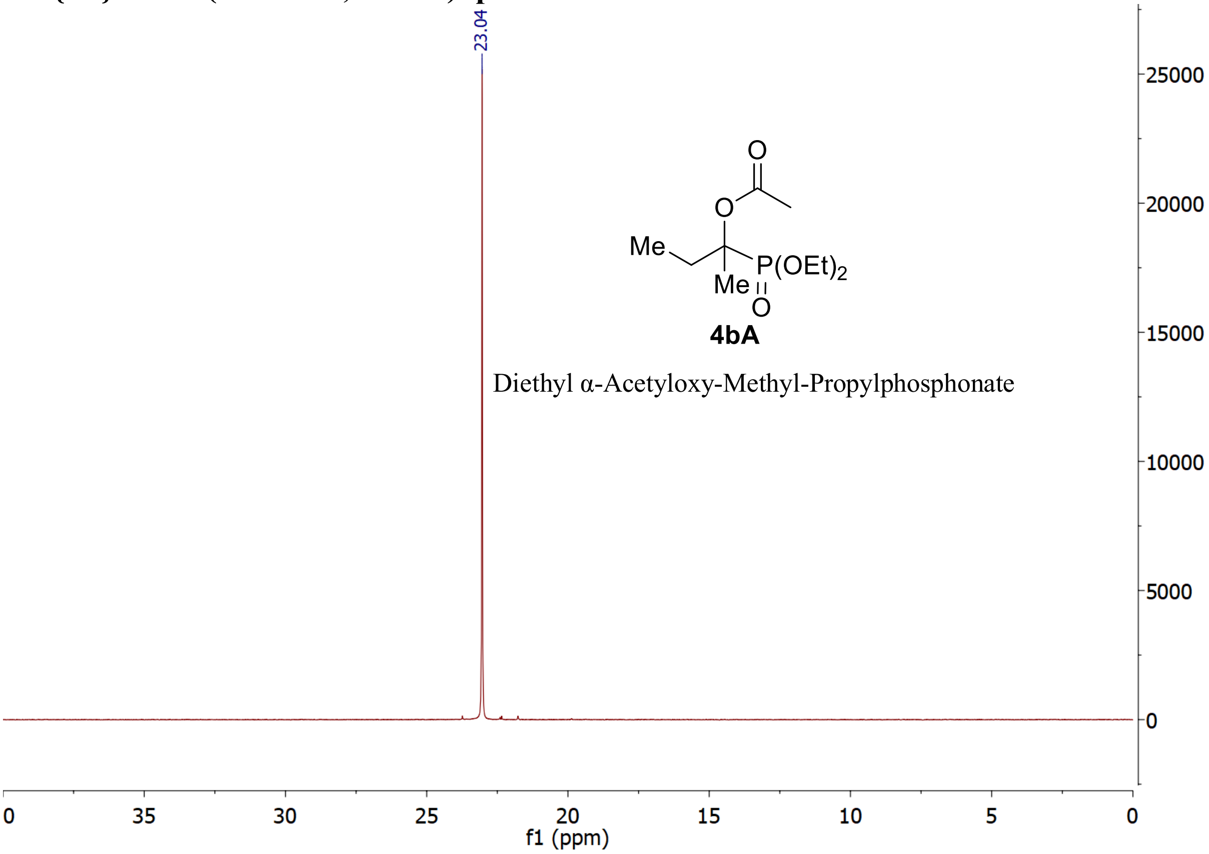

$^{13}C\ \{^{1}H\}$ NMR (75 MHz, $CDCl_3$) spectra for 4bA

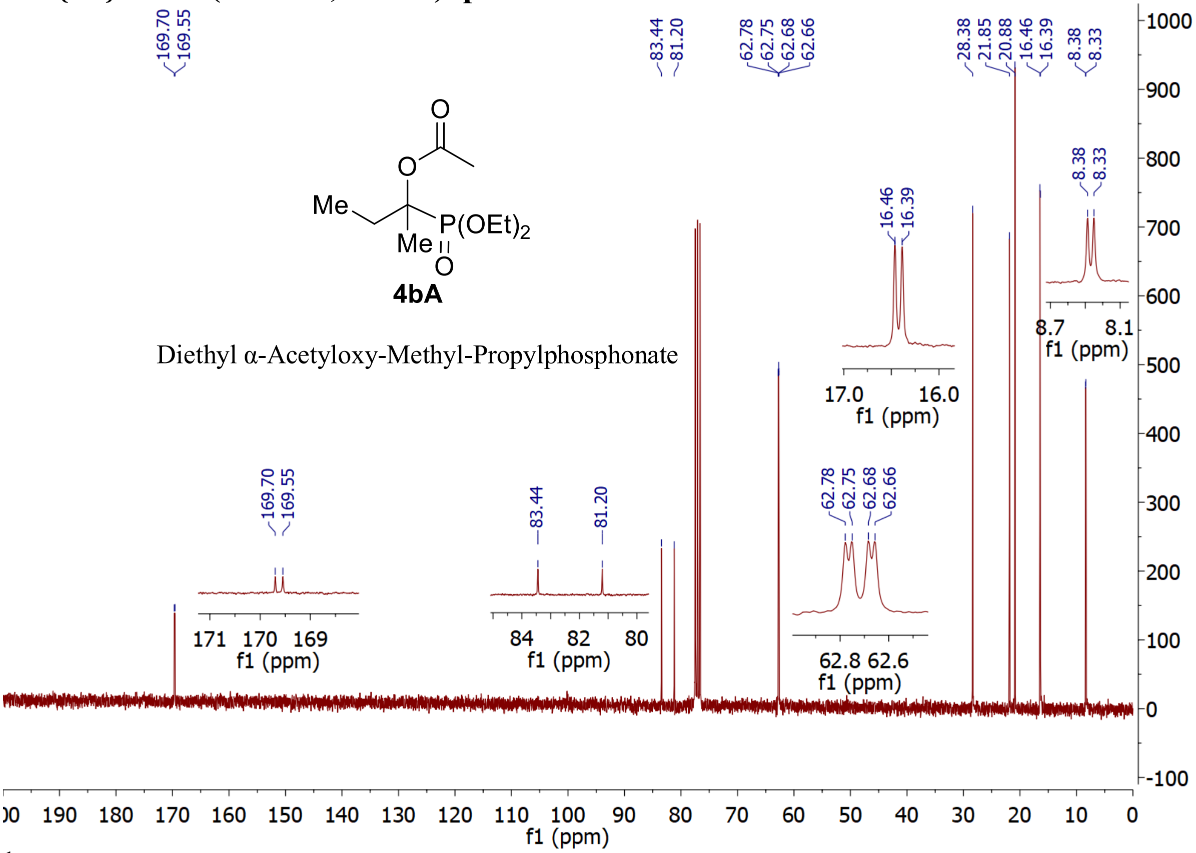

^{1}H NMR (300 MHz, $CDCl_3$) spectra for 4bA

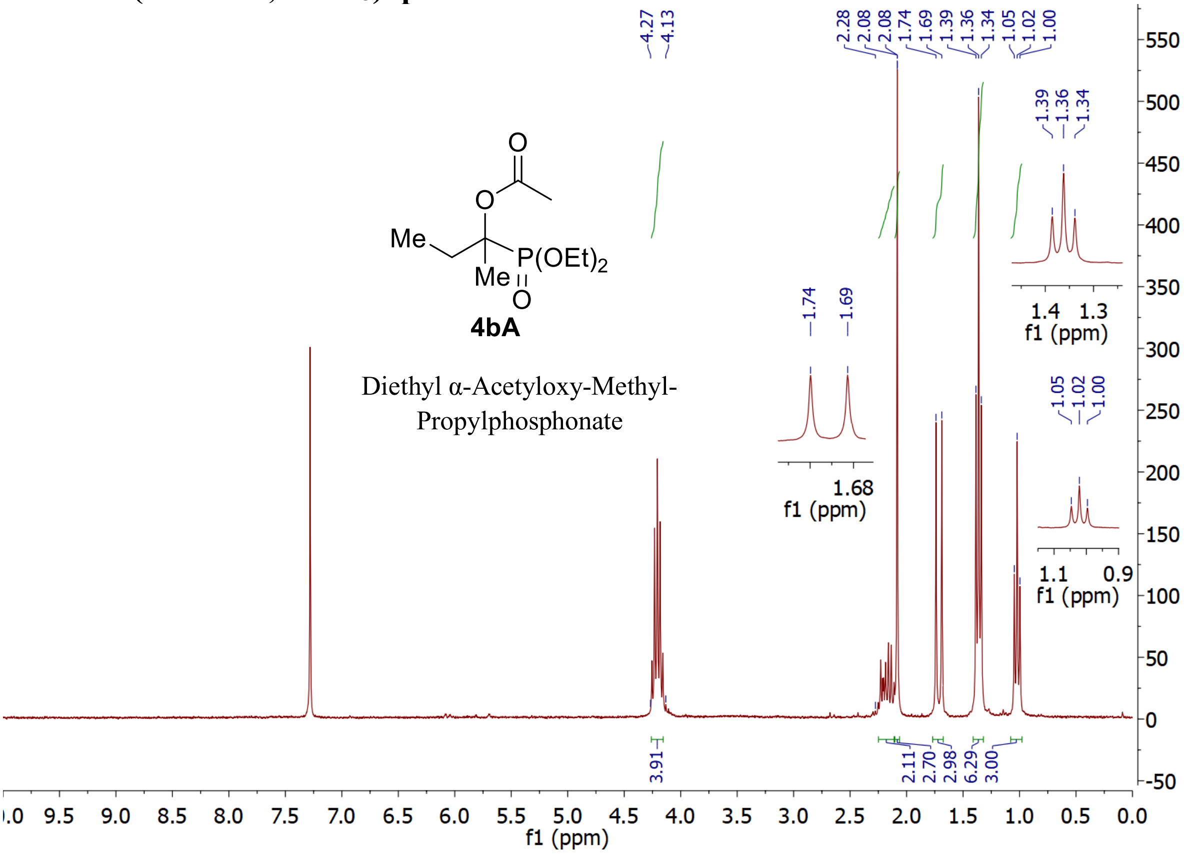

^{31}P {^{1}H} NMR (122 MHz, $CDCl_3$) spectra for 4bB

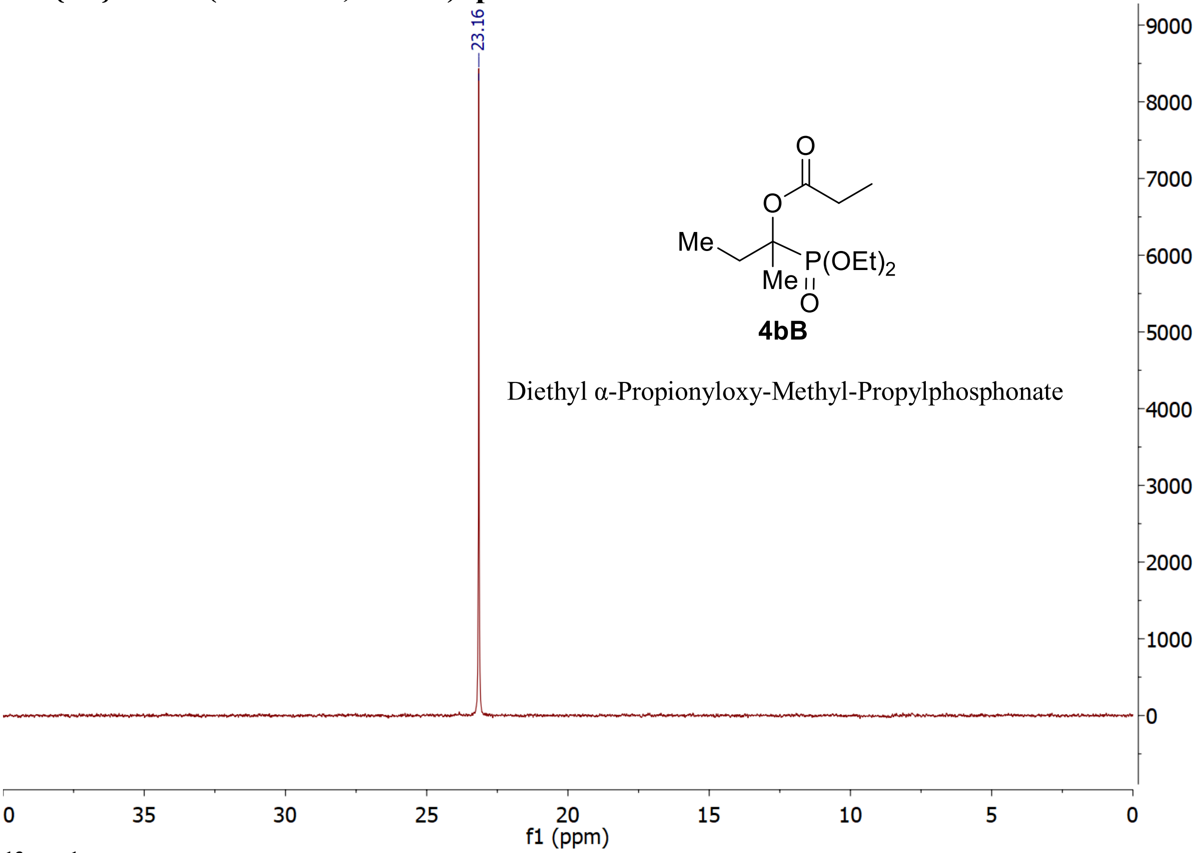

^{13}C {^{1}H} NMR (75 MHz, $CDCl_3$) spectra for 4bB

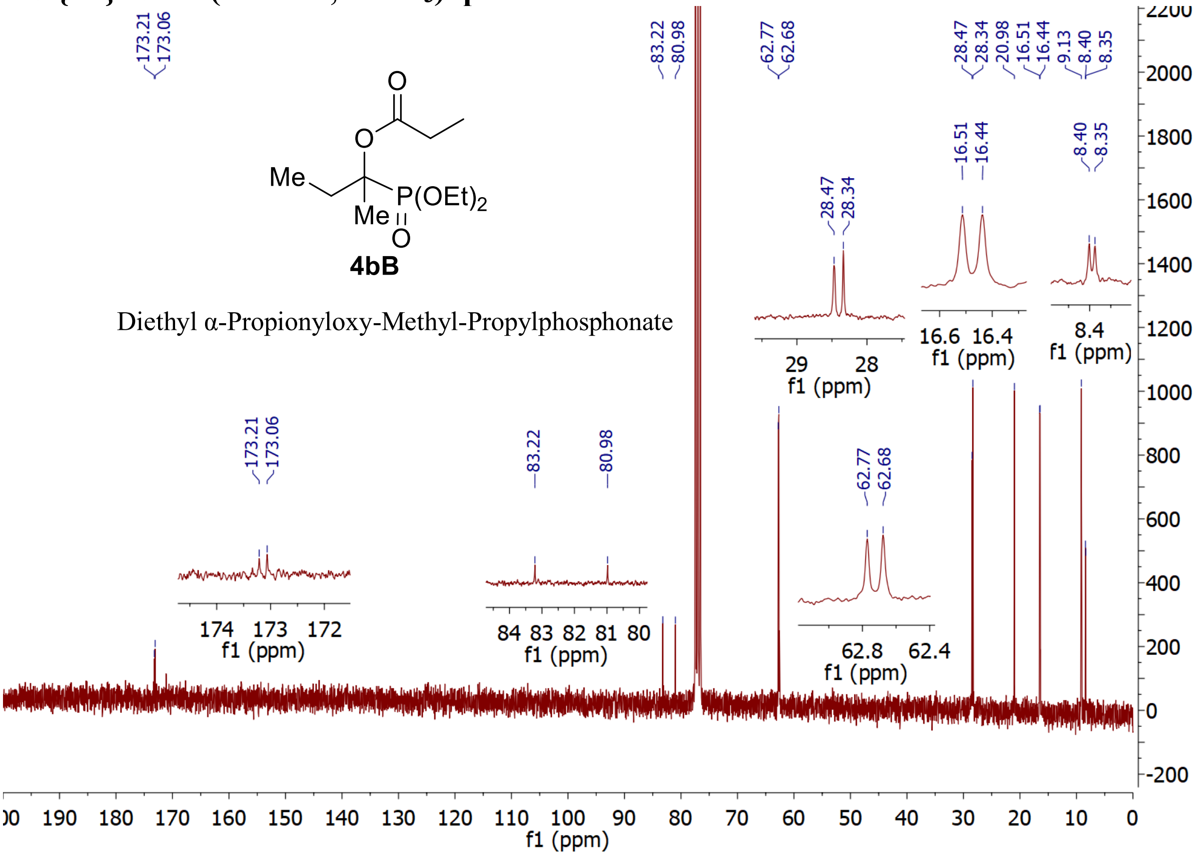

^{1}H NMR (300 MHz, $CDCl_3$) spectra for 4bB

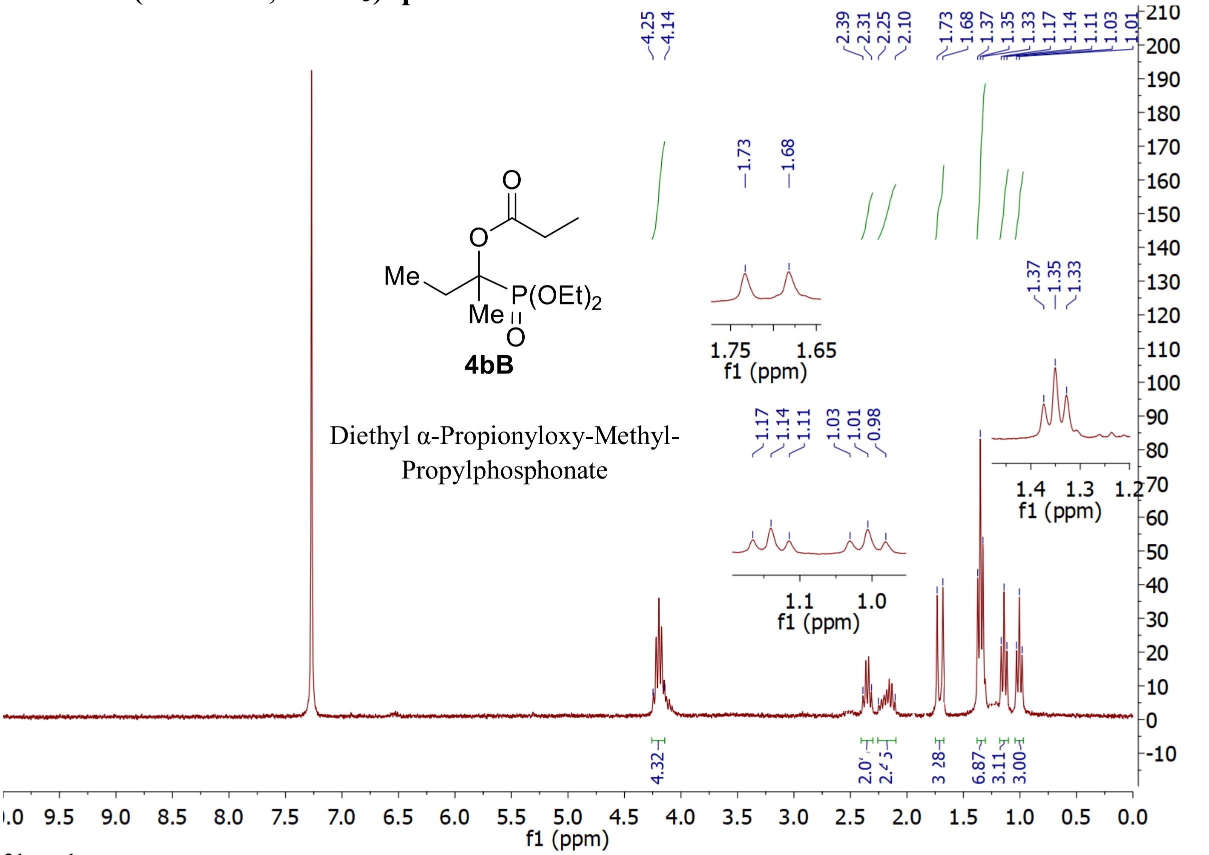

^{31}P {^{1}H} NMR (202 MHz, $CDCl_3$) spectra for 4bC

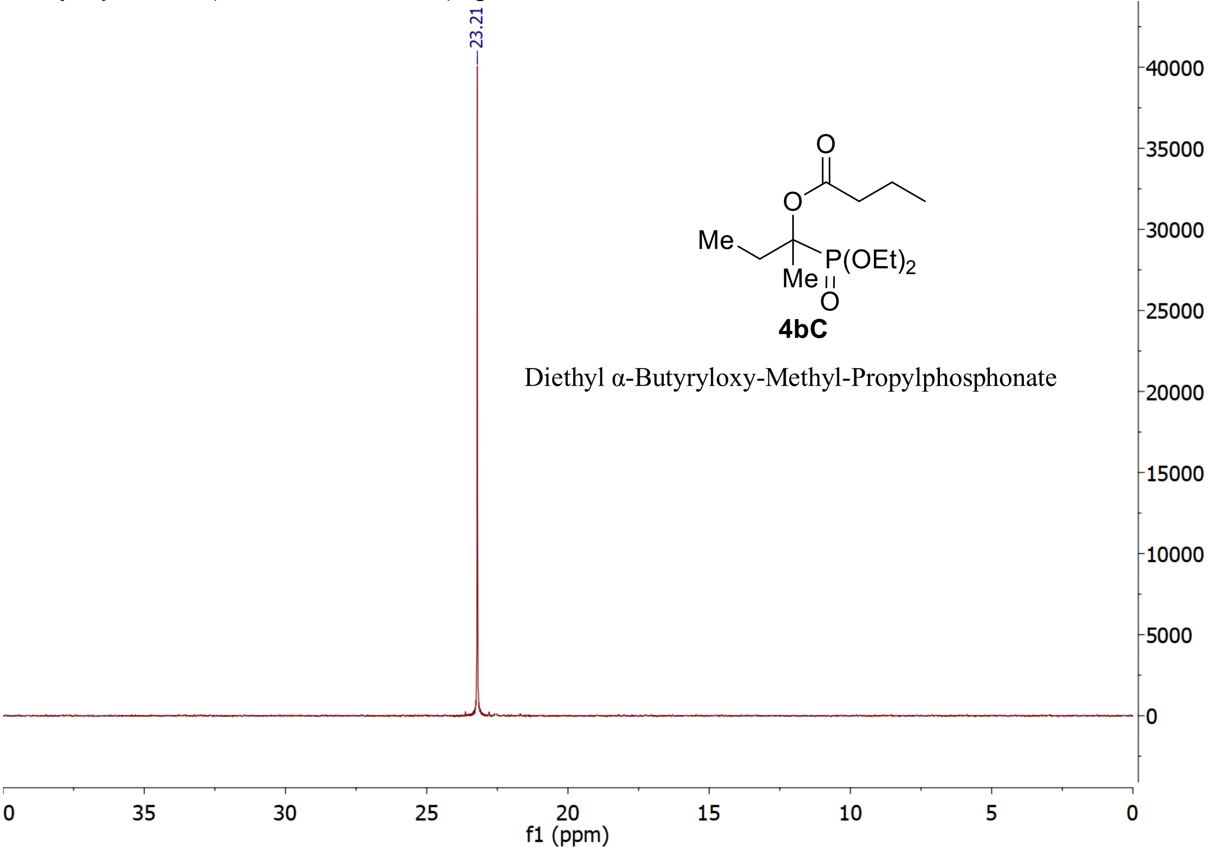

^{13}C {^{1}H} NMR (126 MHz, $CDCl_3$) spectra for 4bC

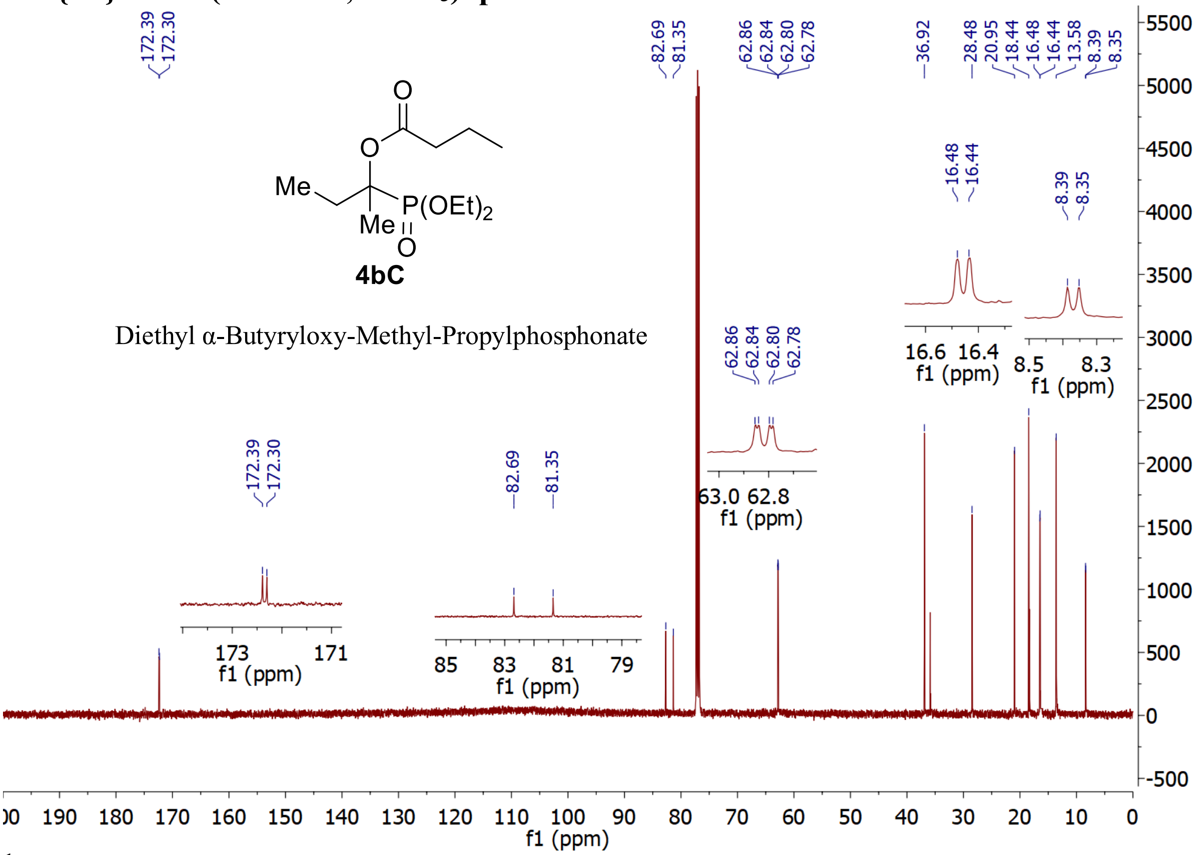

^{1}H NMR (500 MHz, $CDCl_3$) spectra for 4bC

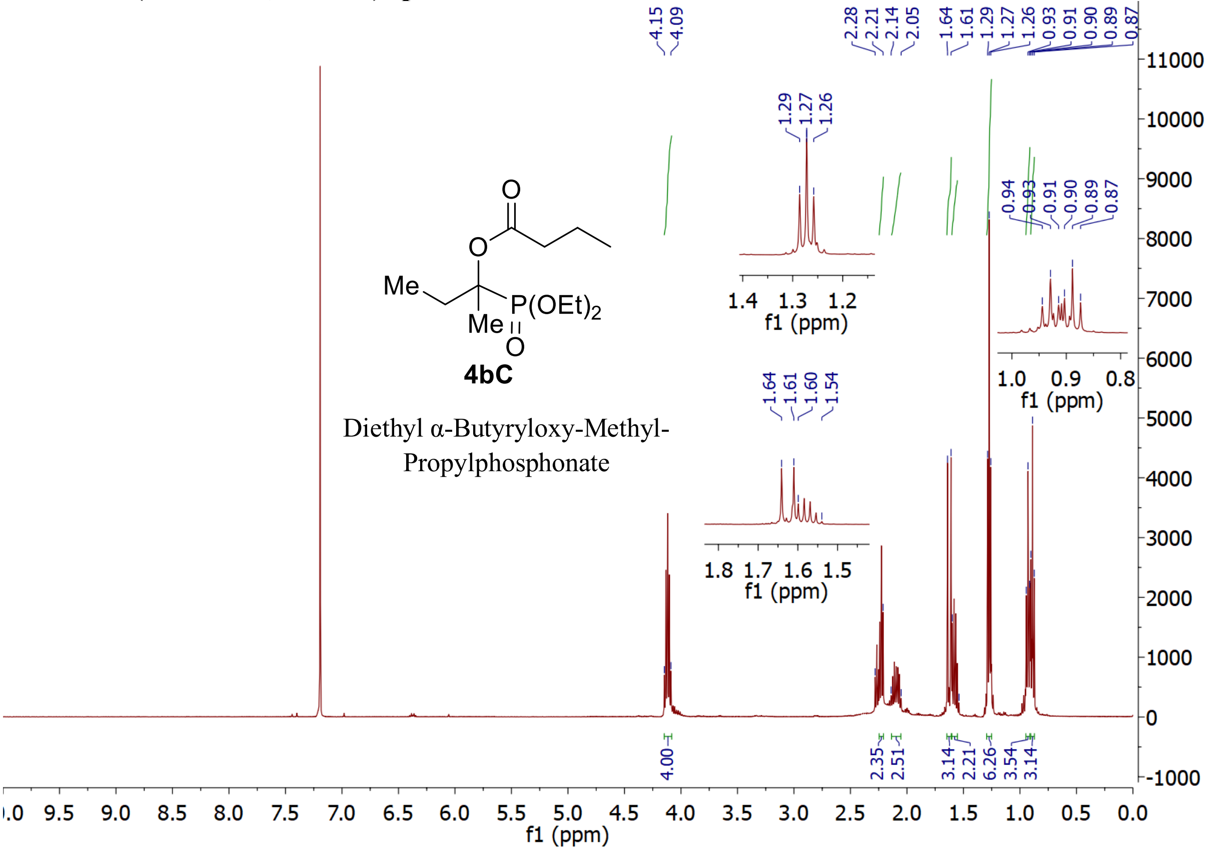

^{31}P {^{1}H} NMR (122 MHz, $CDCl_3$) spectra for 4cA

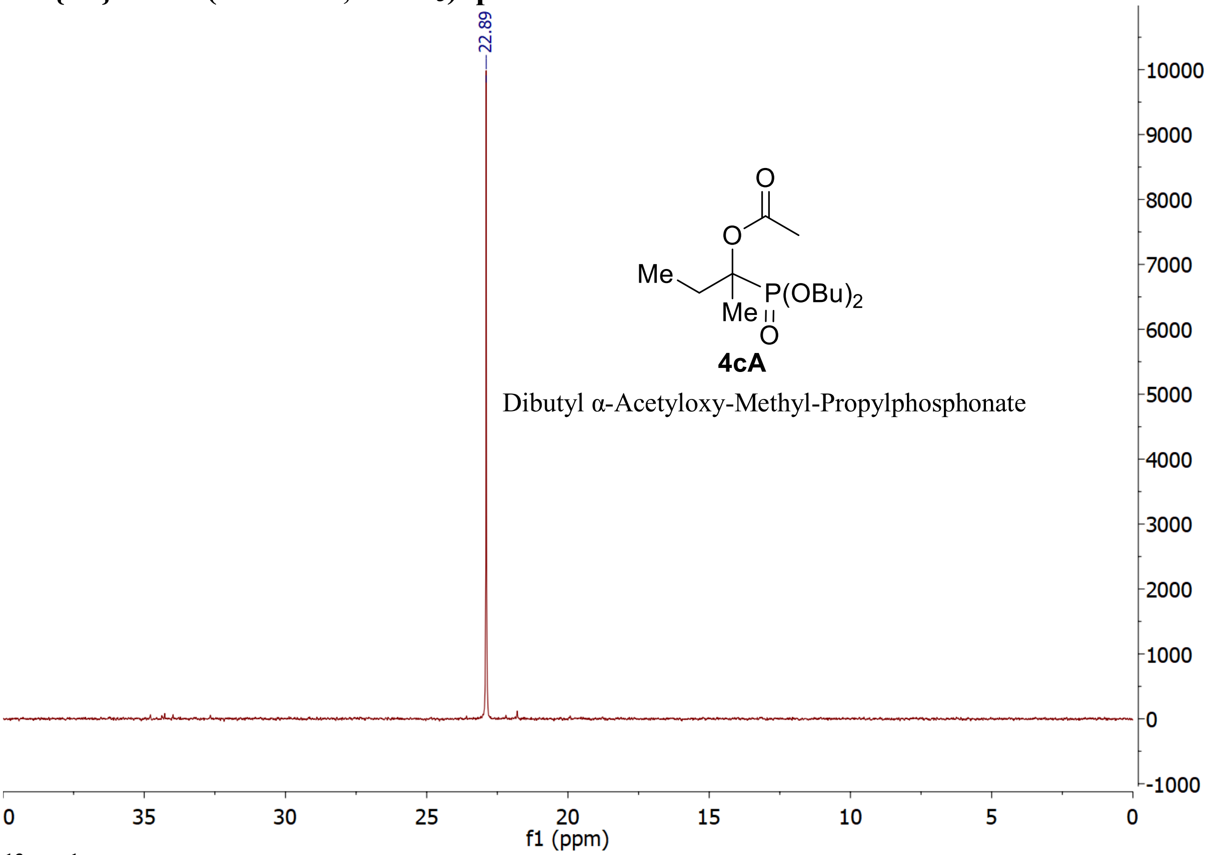

^{13}C {^{1}H} NMR (75 MHz, $CDCl_3$) spectra for 4cA

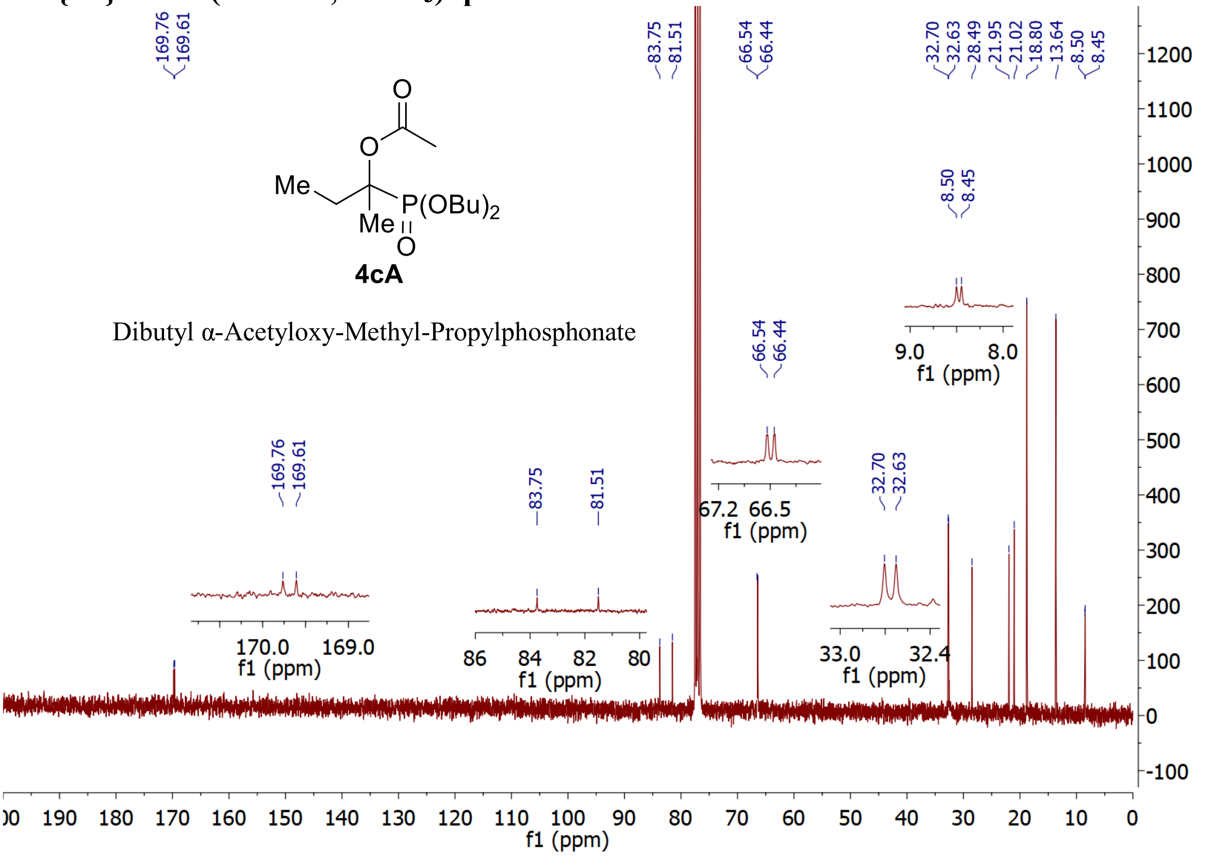

^{1}H NMR (300 MHz, $CDCl_3$) spectra for 4cA

Dibutyl α-Acetyloxy-Methyl-Propylphosphonate

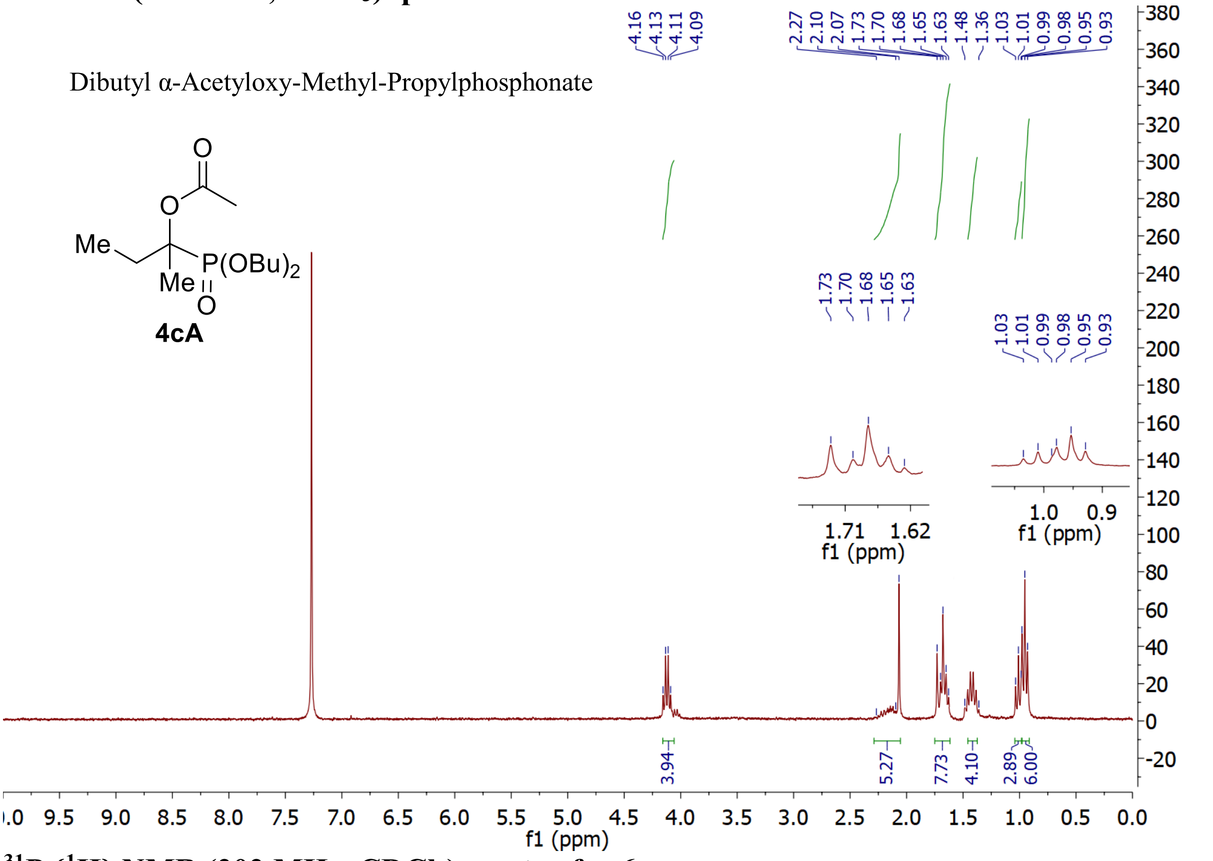

^{31}P {^{1}H} NMR (202 MHz, $CDCl_3$) spectra for 6a

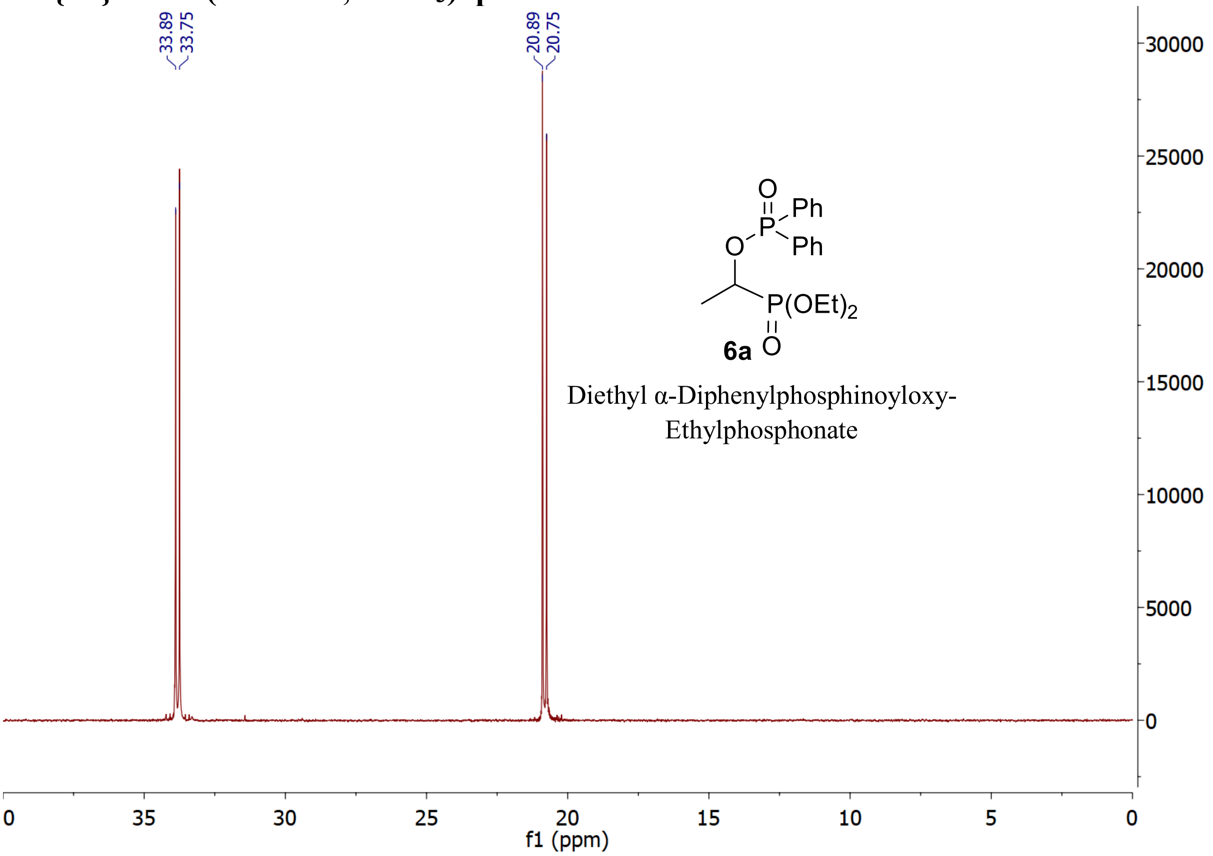

Diethyl α-Diphenylphosphinoyloxy-Ethylphosphonate

^{13}C {^{1}H} NMR (126 MHz, $CDCl_3$) spectra for 6a

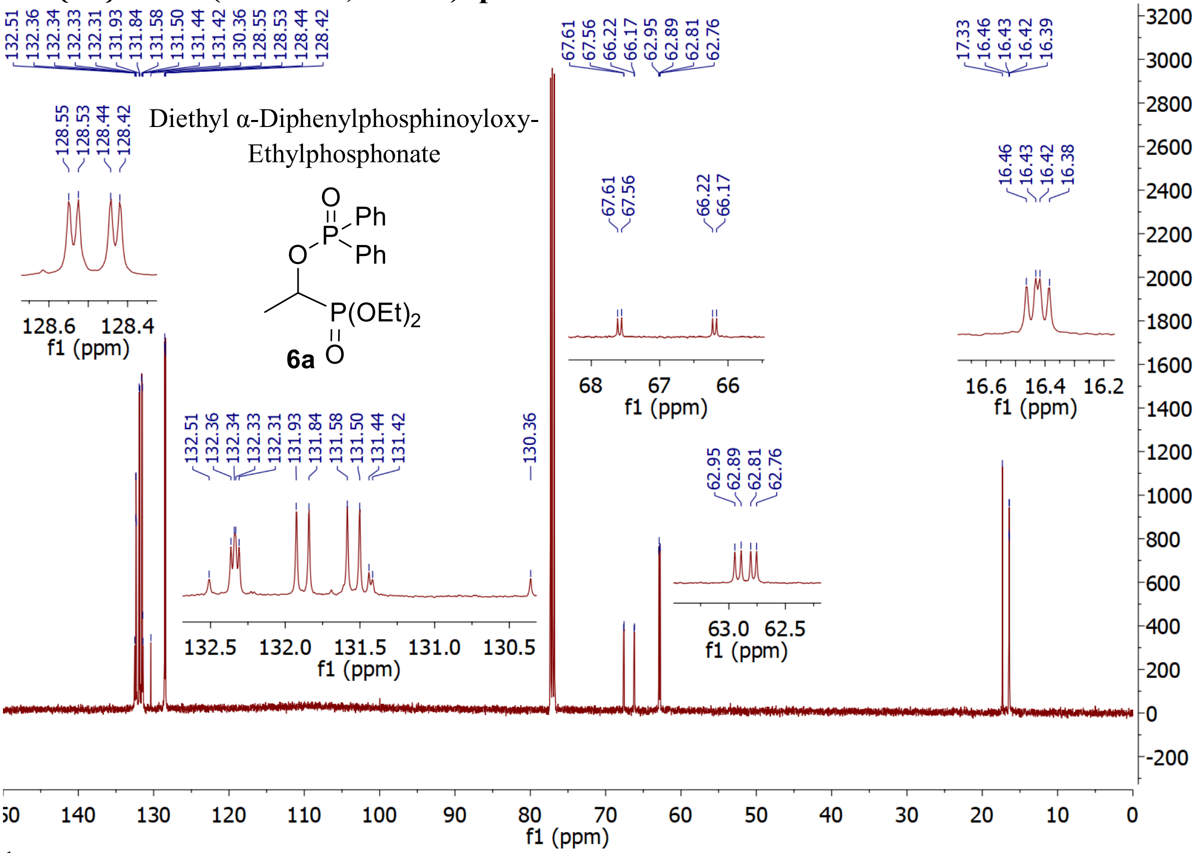

^{1}H NMR (500 MHz, $CDCl_3$) spectra for 6a

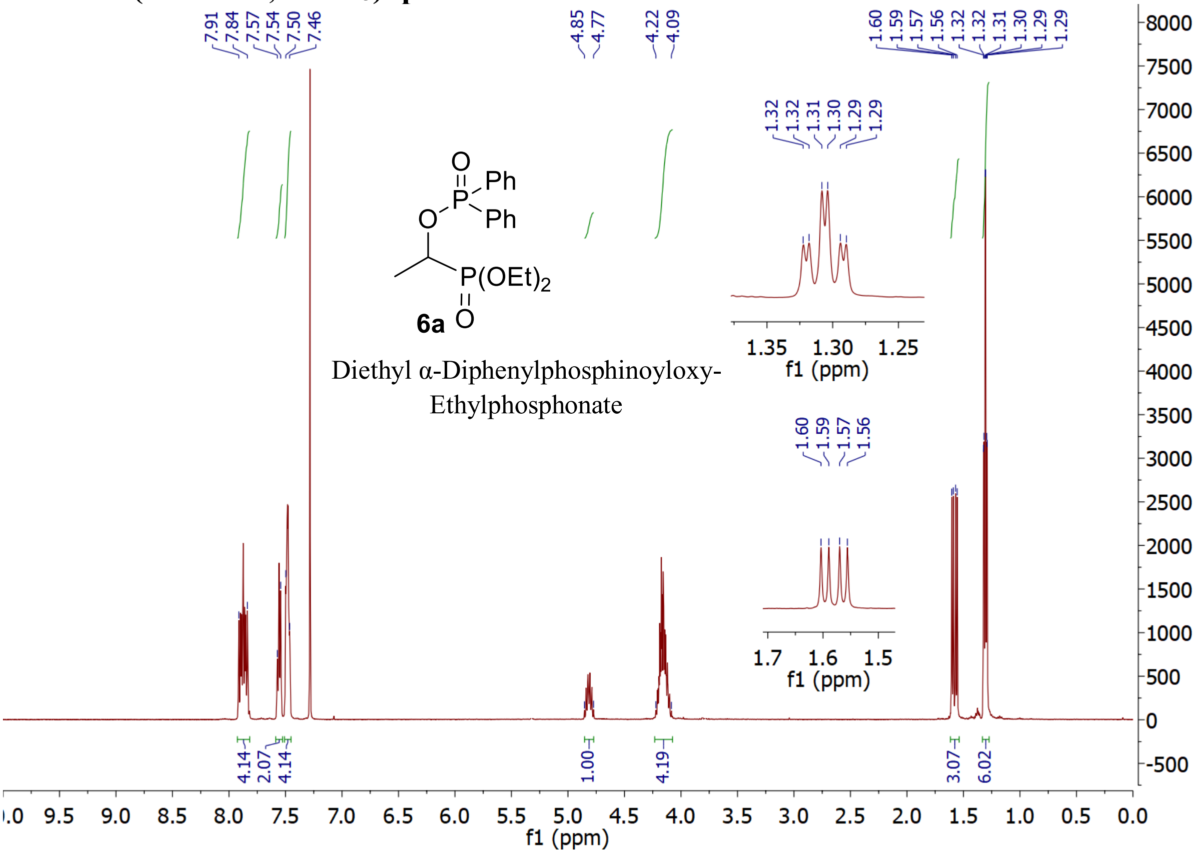

^{31}P {^{1}H} NMR (122 MHz, CDCl$_3$) spectra for 6b

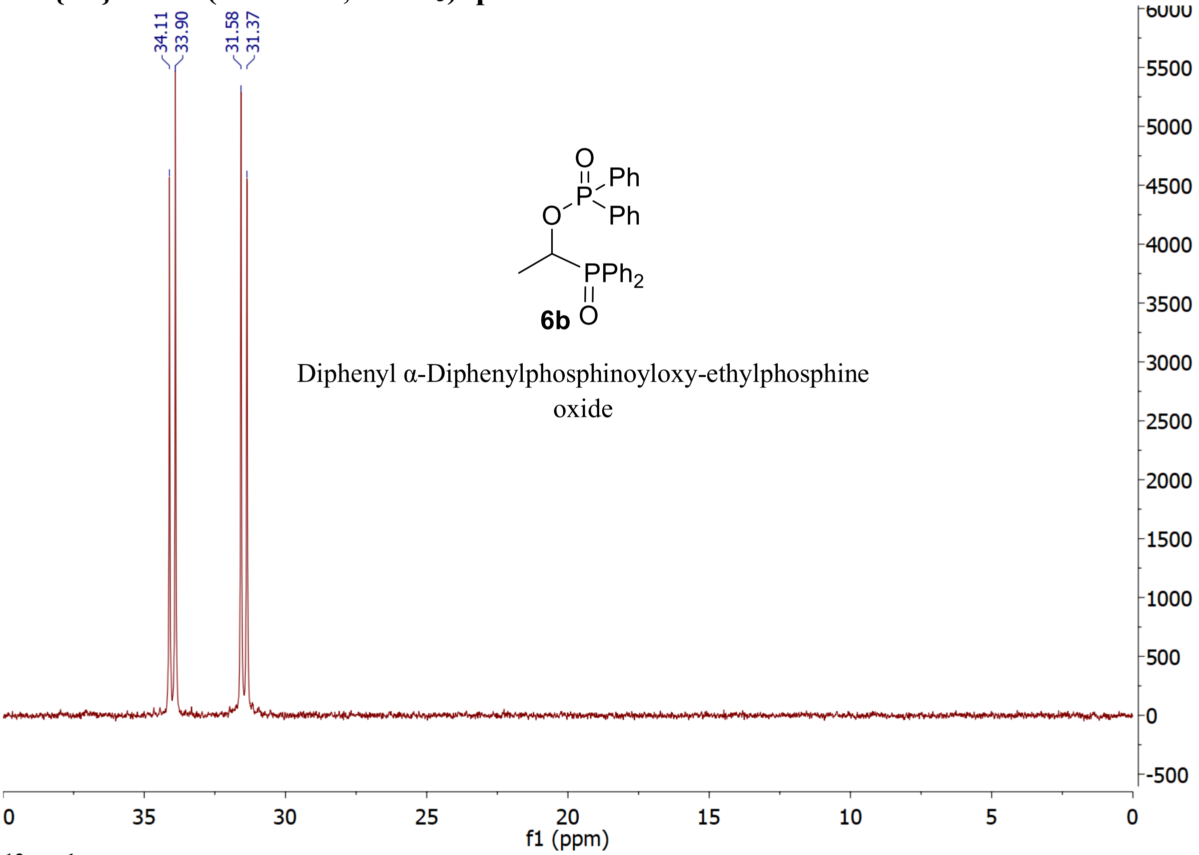

^{13}P {^{1}H} NMR (126 MHz, CDCl$_3$) spectra for 6b

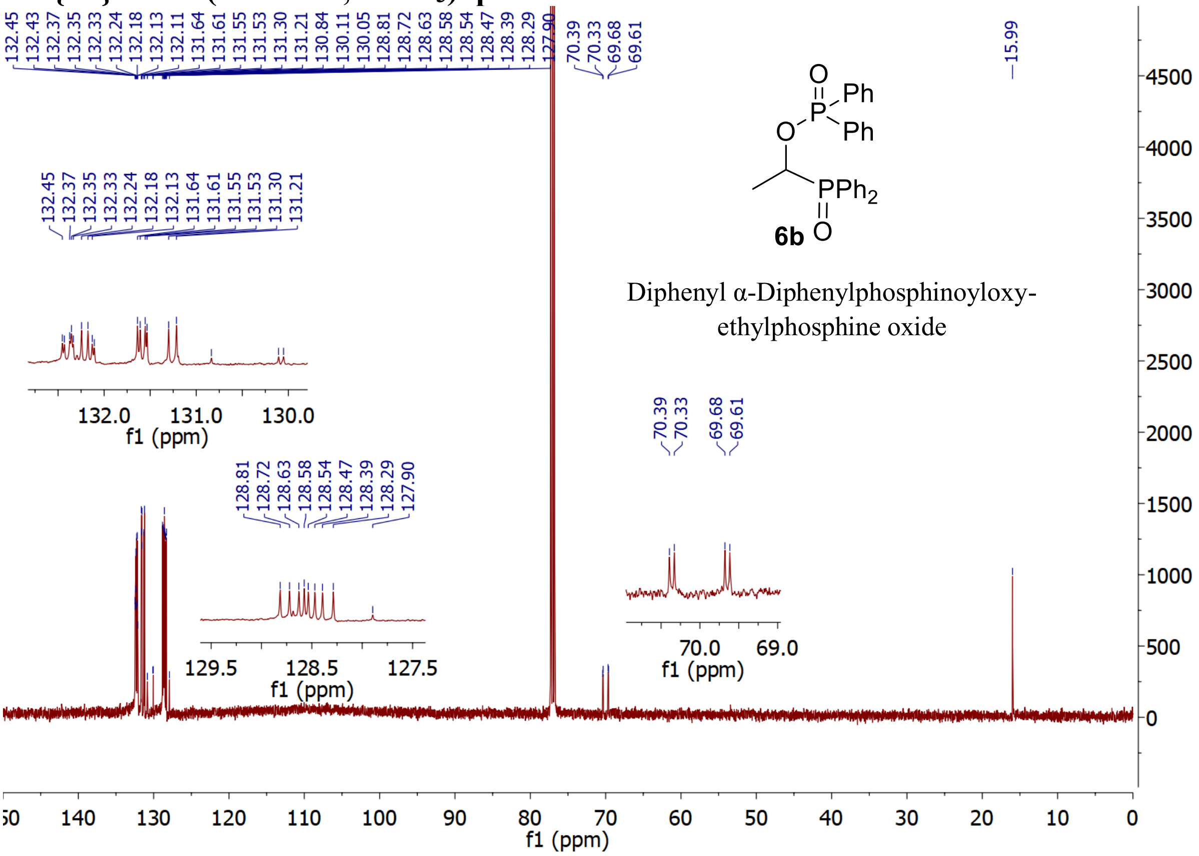

^{1}H NMR (500 MHz, $CDCl_3$) spectra for 6b

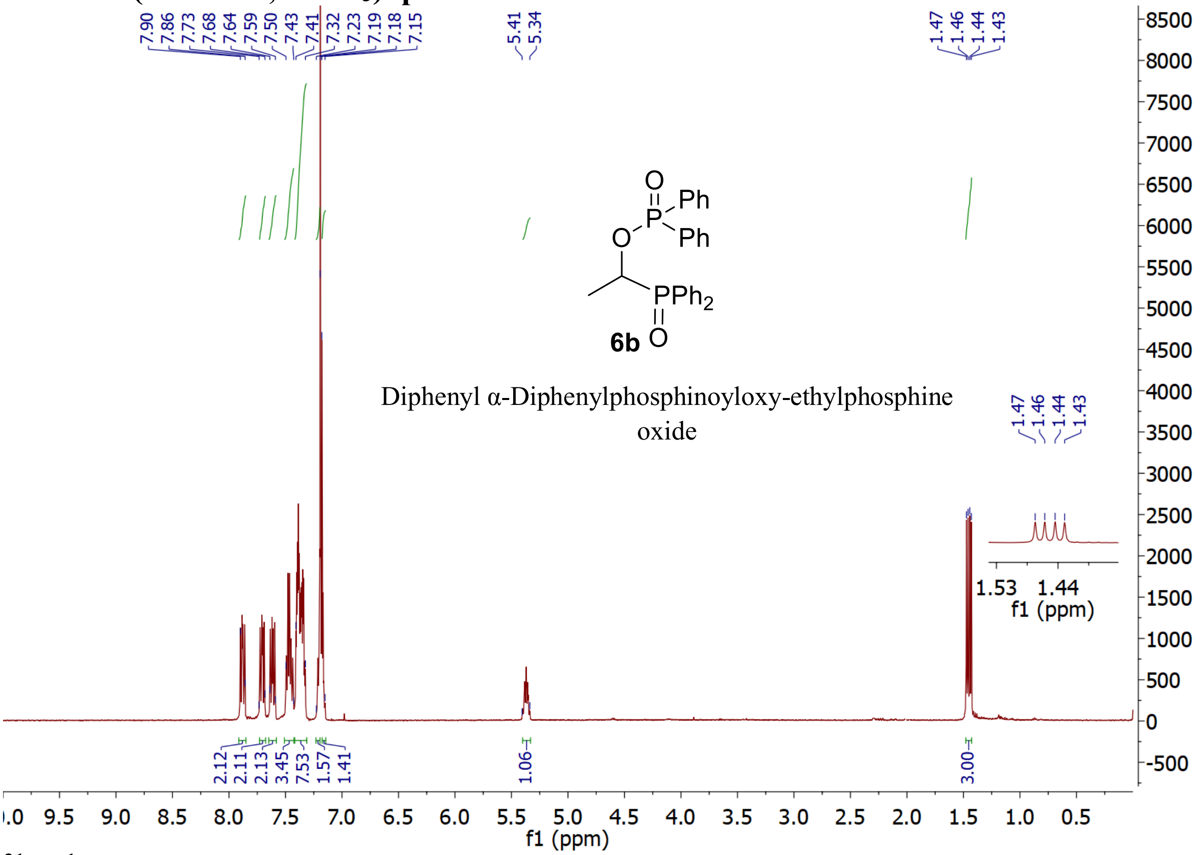

^{31}P {^{1}H} NMR (202 MHz, $CDCl_3$) spectra for 6c

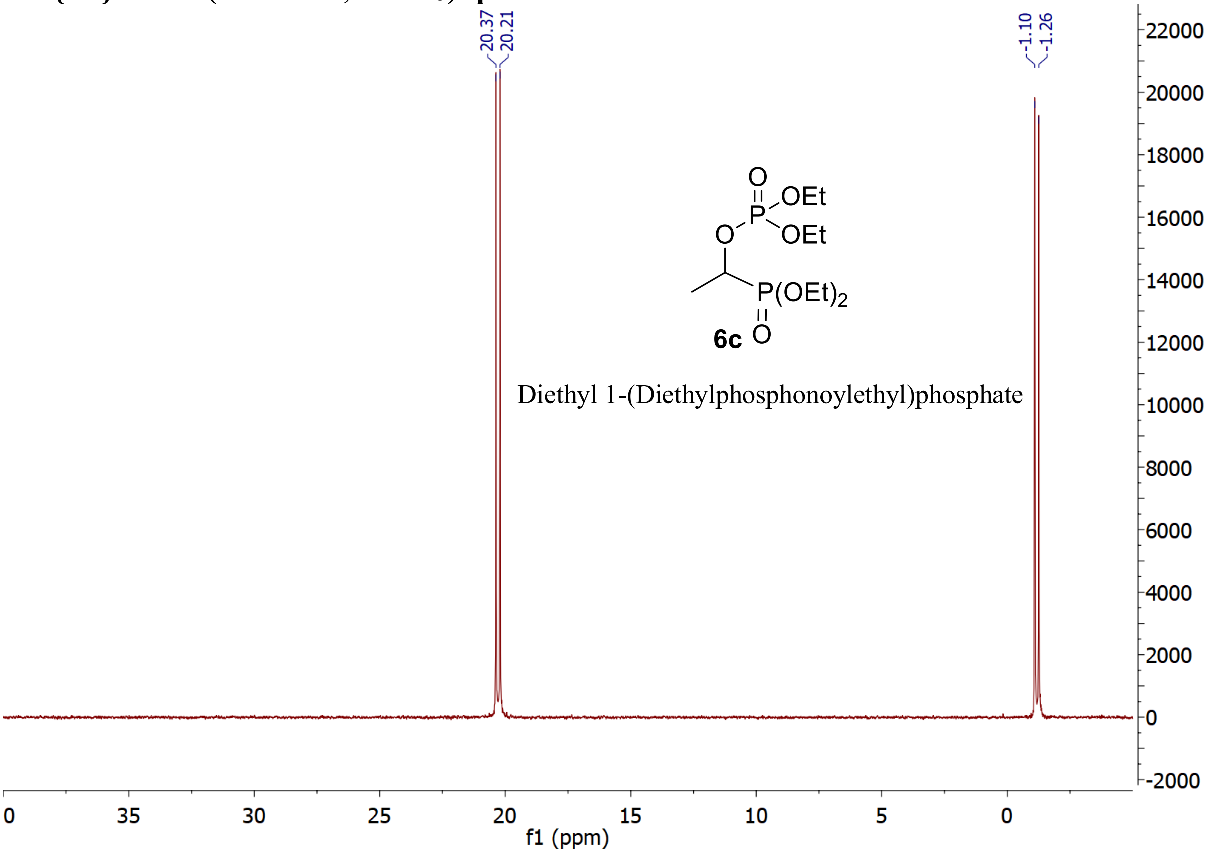

$^{31}P\ \{^1H\}$ NMR (202 MHz, $CDCl_3$) spectra for 8a

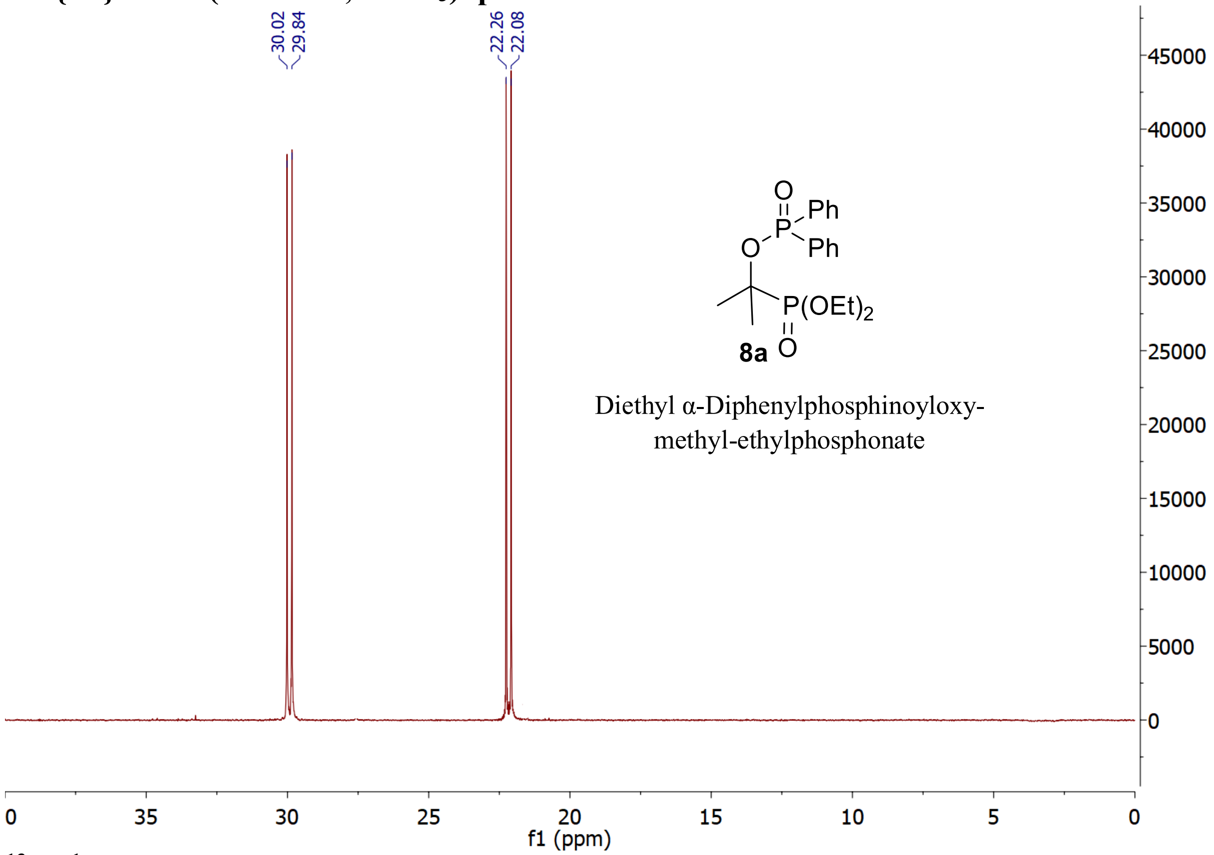

$^{13}C\ \{^1H\}$ NMR (75 MHz, $CDCl_3$) spectra for 8a

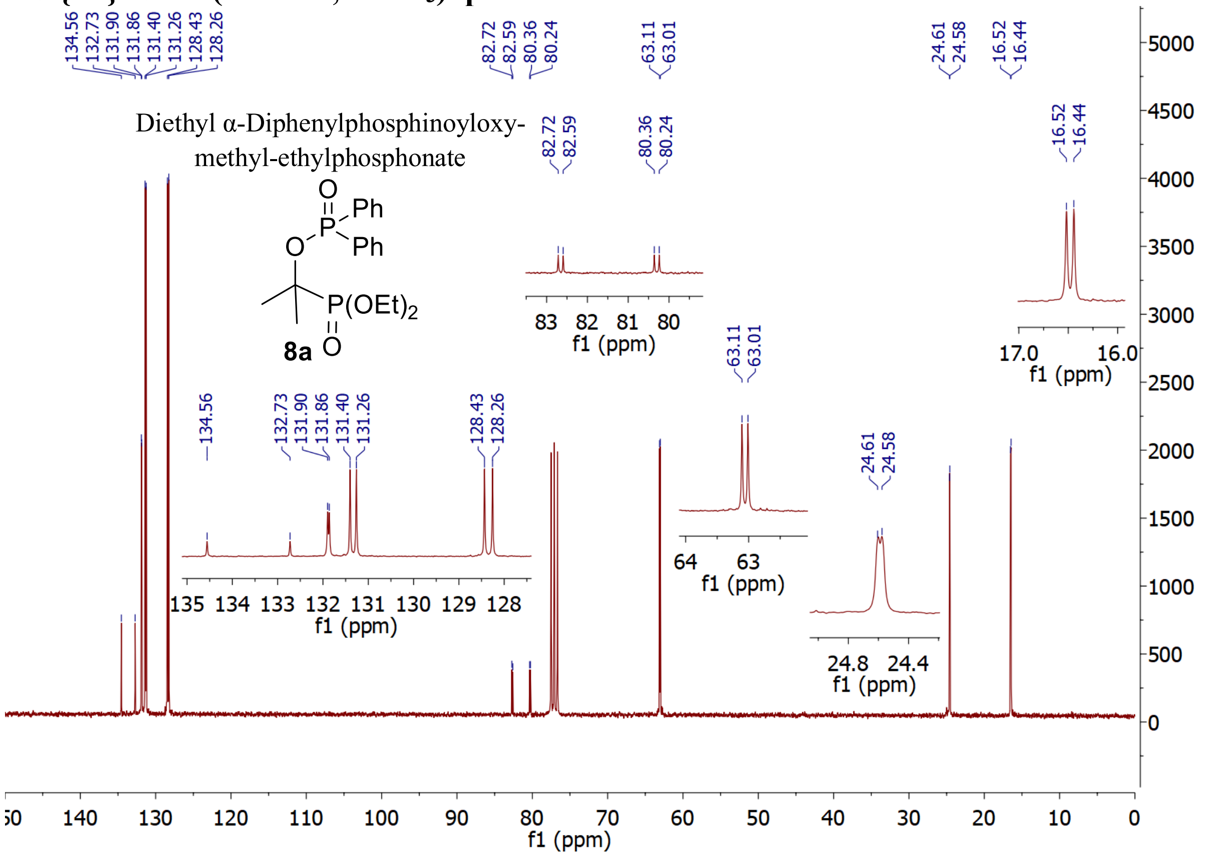

^{1}H NMR (500 MHz, $CDCl_3$) spectra for 8a

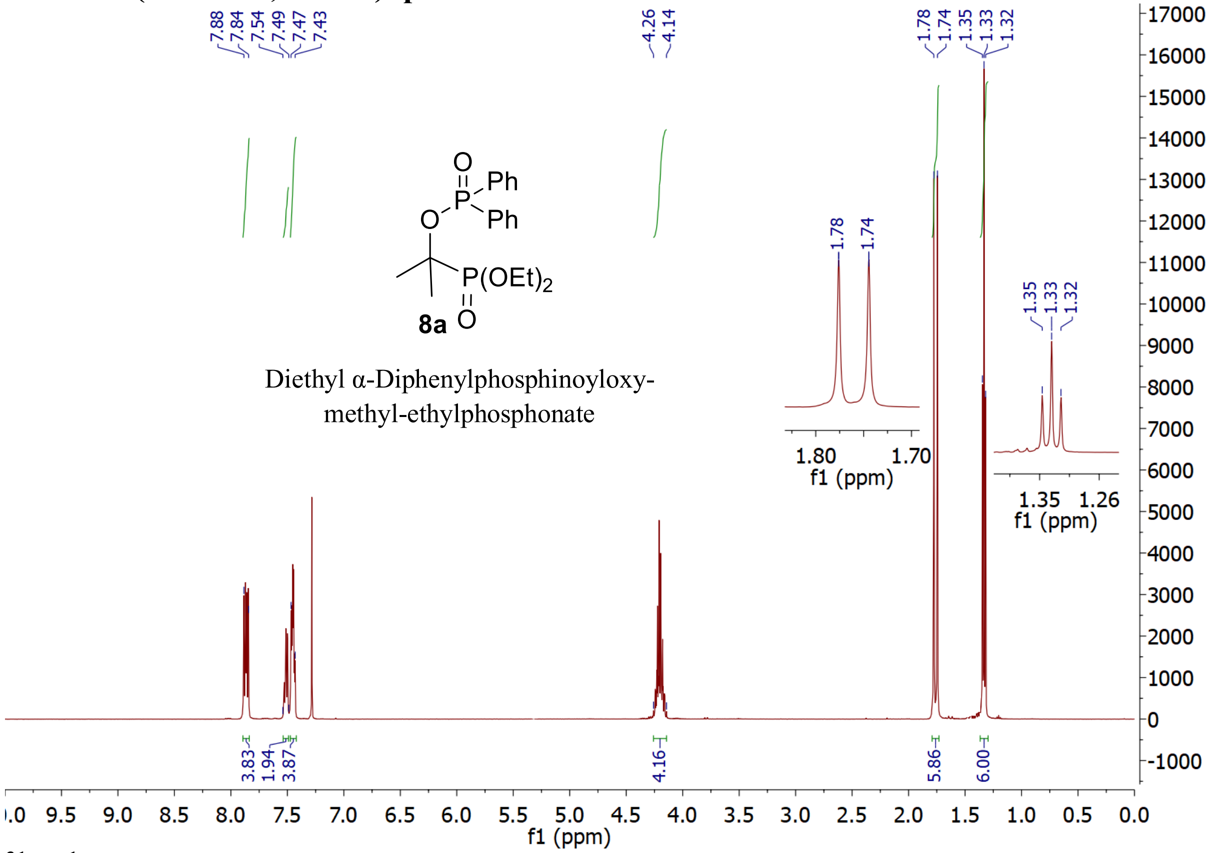

^{31}P {^{1}H} NMR (122 MHz, $CDCl_3$) spectra for 8b

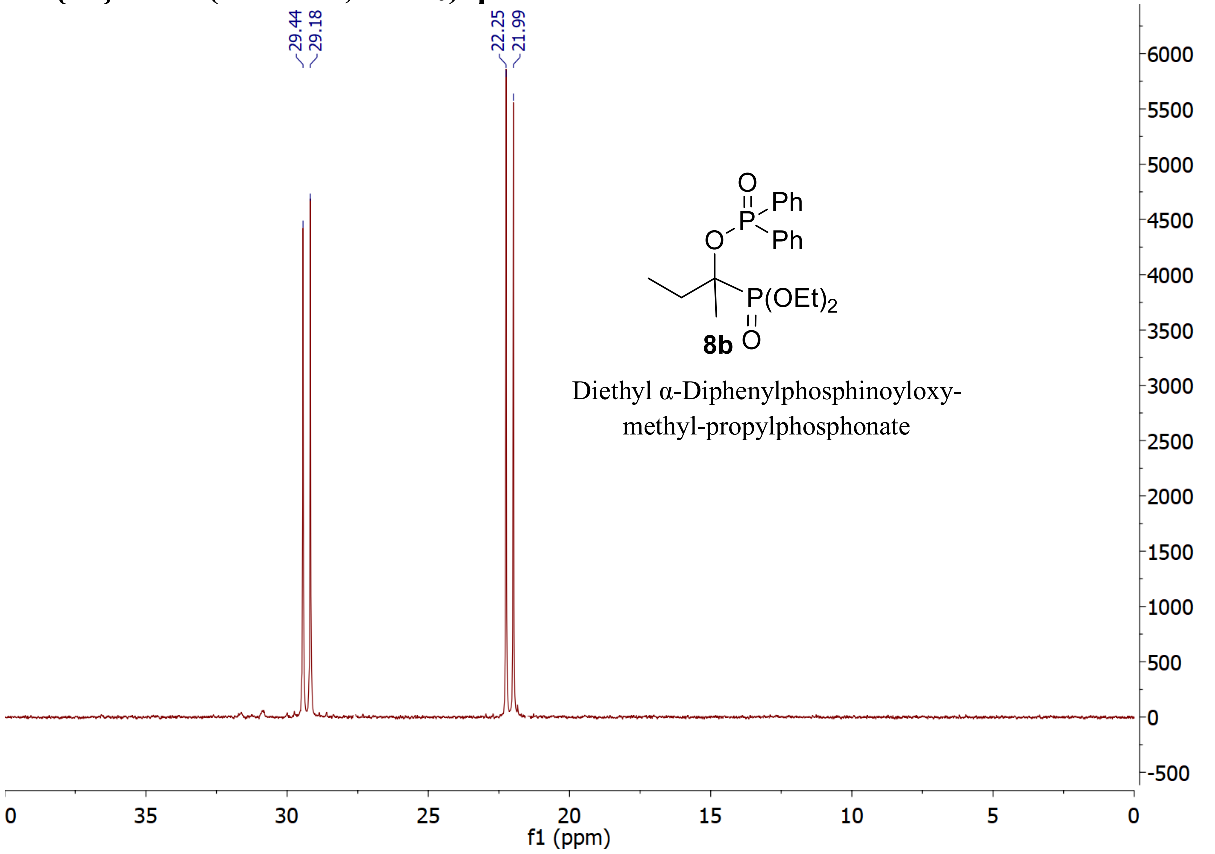

^{13}C {^{1}H} NMR (75 MHz, $CDCl_3$) spectra for 8b

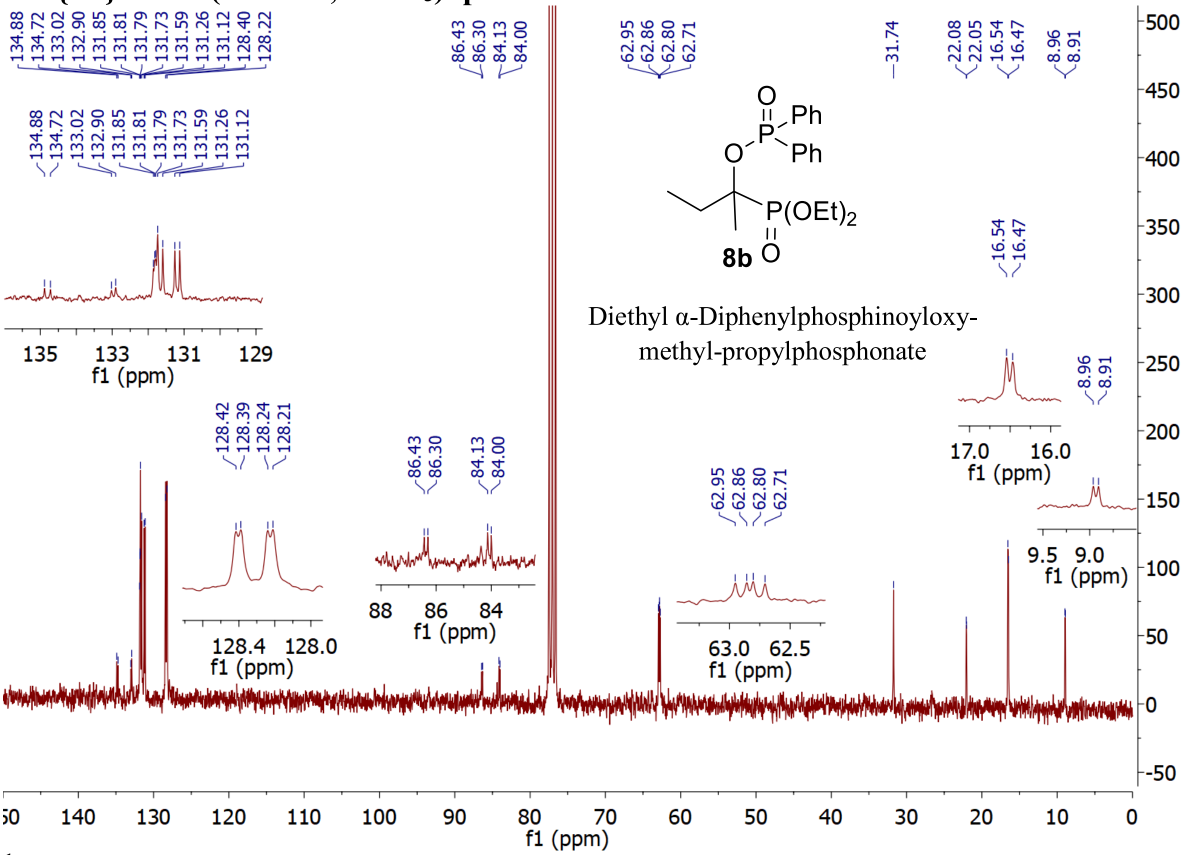

^{1}H NMR (300 MHz, $CDCl_3$) spectra for 8b

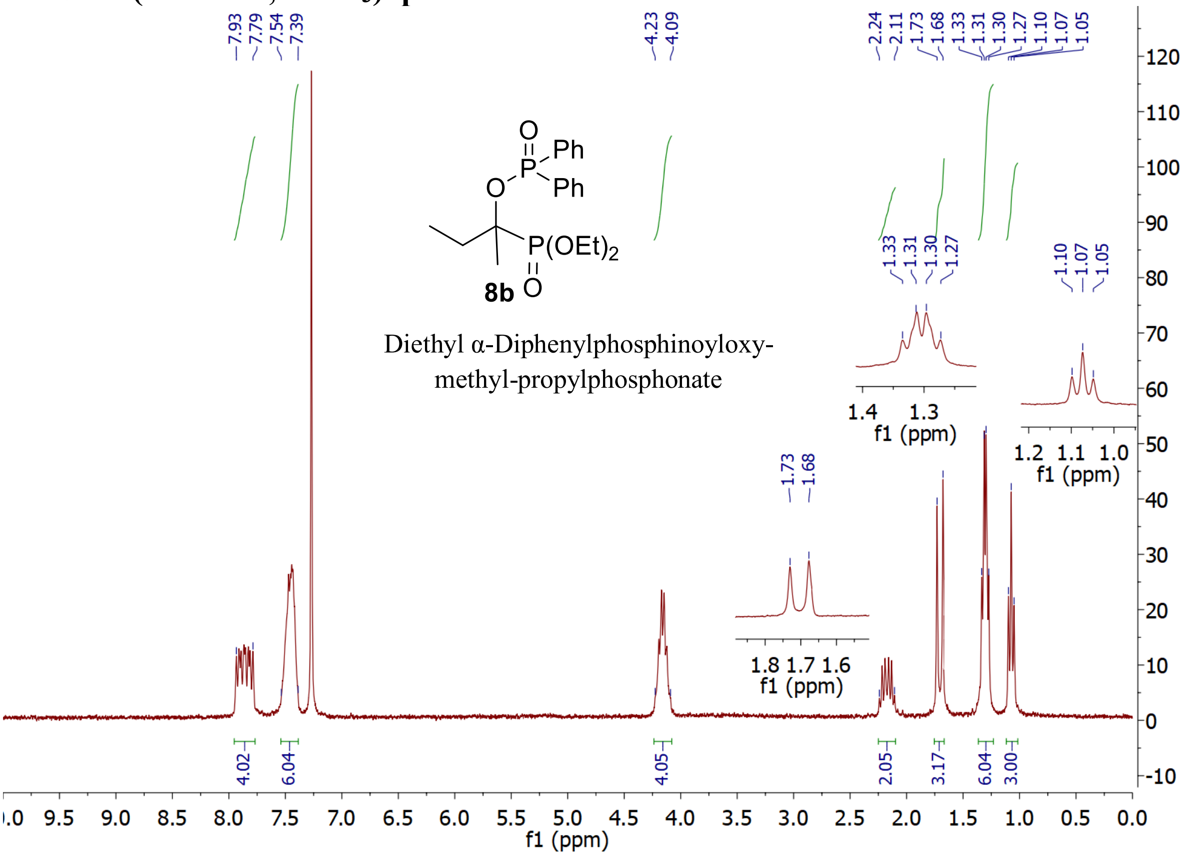

^{31}P {^{1}H} NMR (202 MHz, $CDCl_3$) spectra for 9a

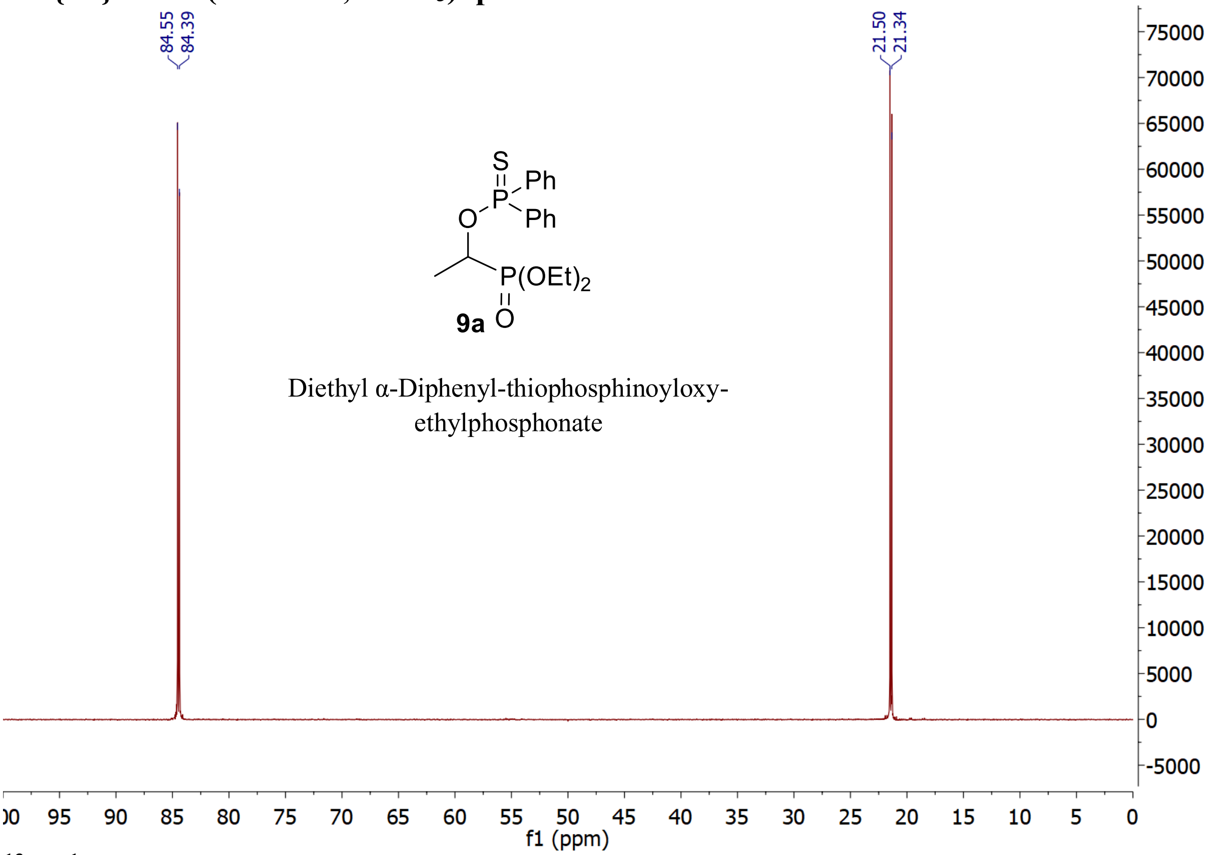

^{13}C {^{1}H} NMR (122 MHz, $CDCl_3$) spectra for 9a

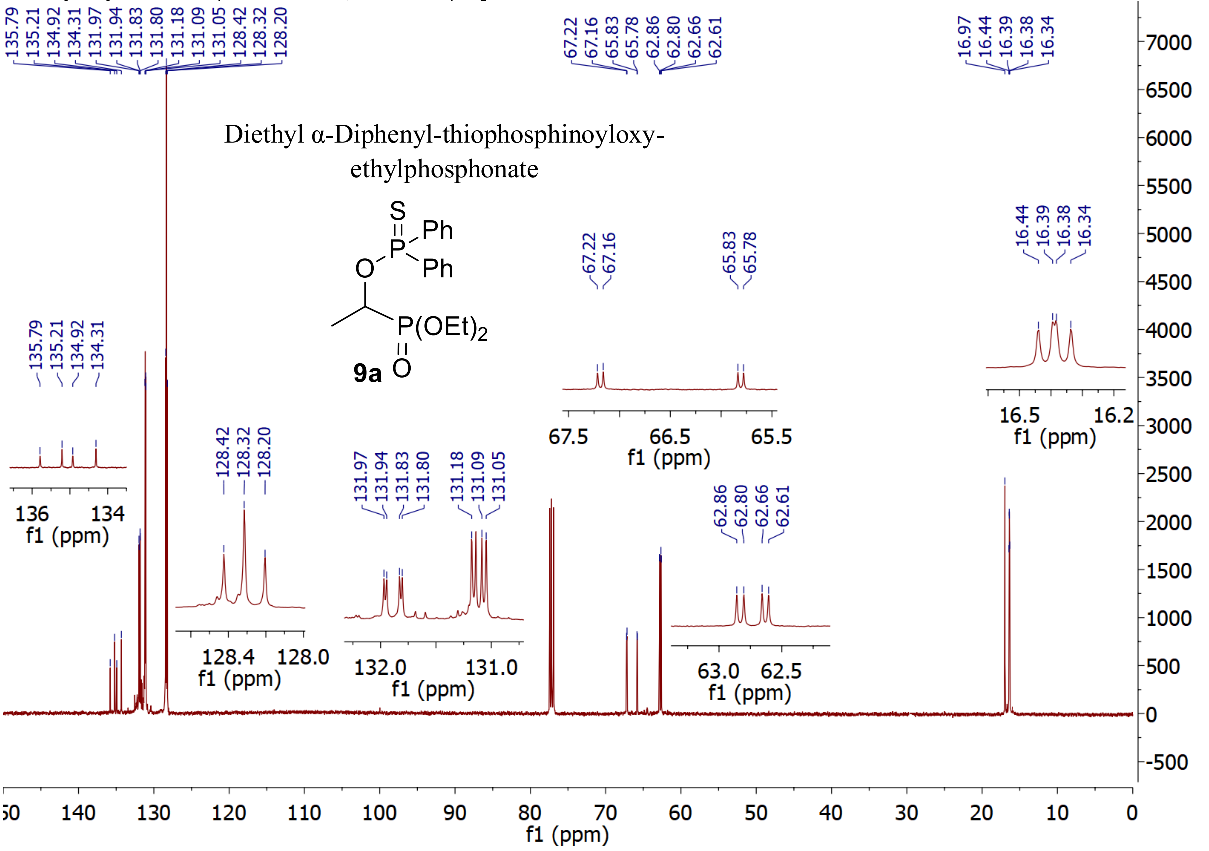

^{1}H NMR (500 MHz, CDCl$_3$) spectra for 9a

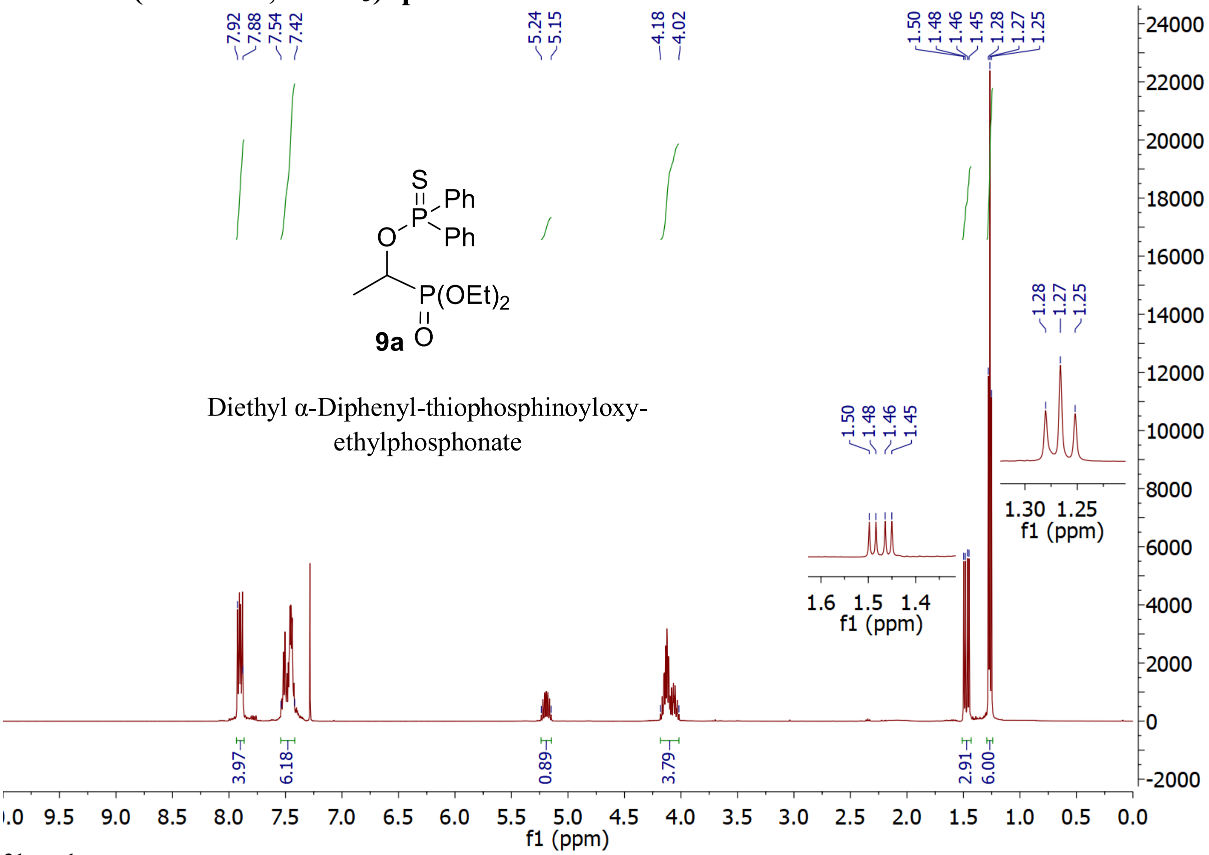

^{31}P {^{1}H} NMR (202 MHz, CDCl$_3$) spectra for 9b

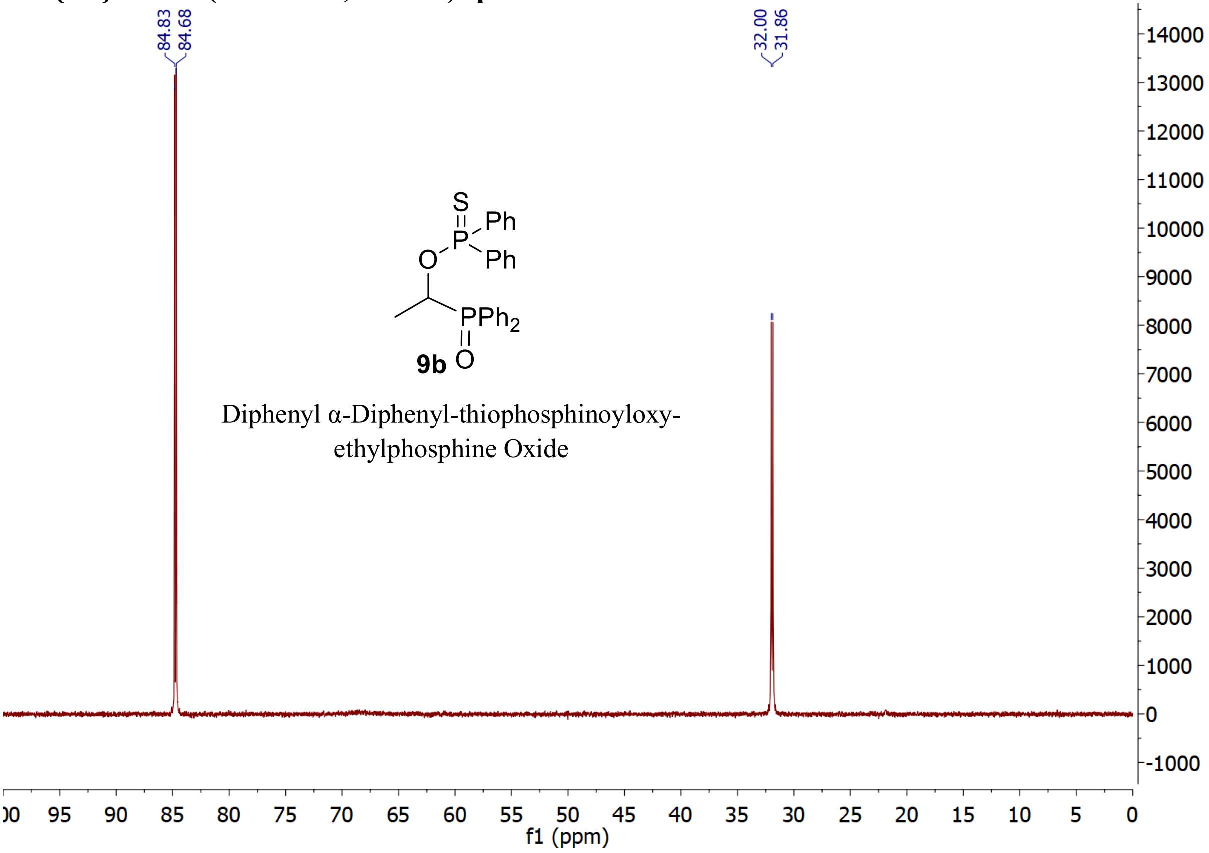

^{13}C {^{1}H} NMR (126 MHz, $CDCl_3$) spectra for 9b

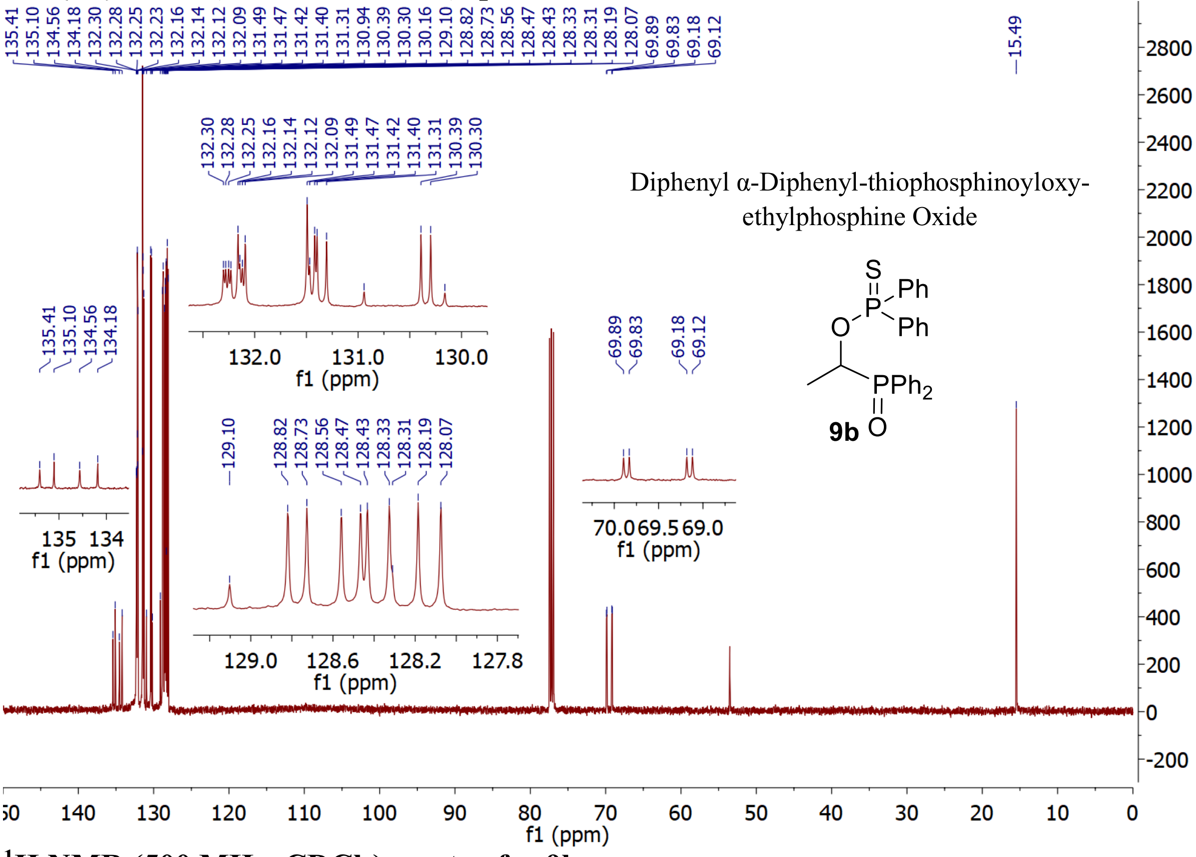

^{1}H NMR (500 MHz, $CDCl_3$) spectra for 9b

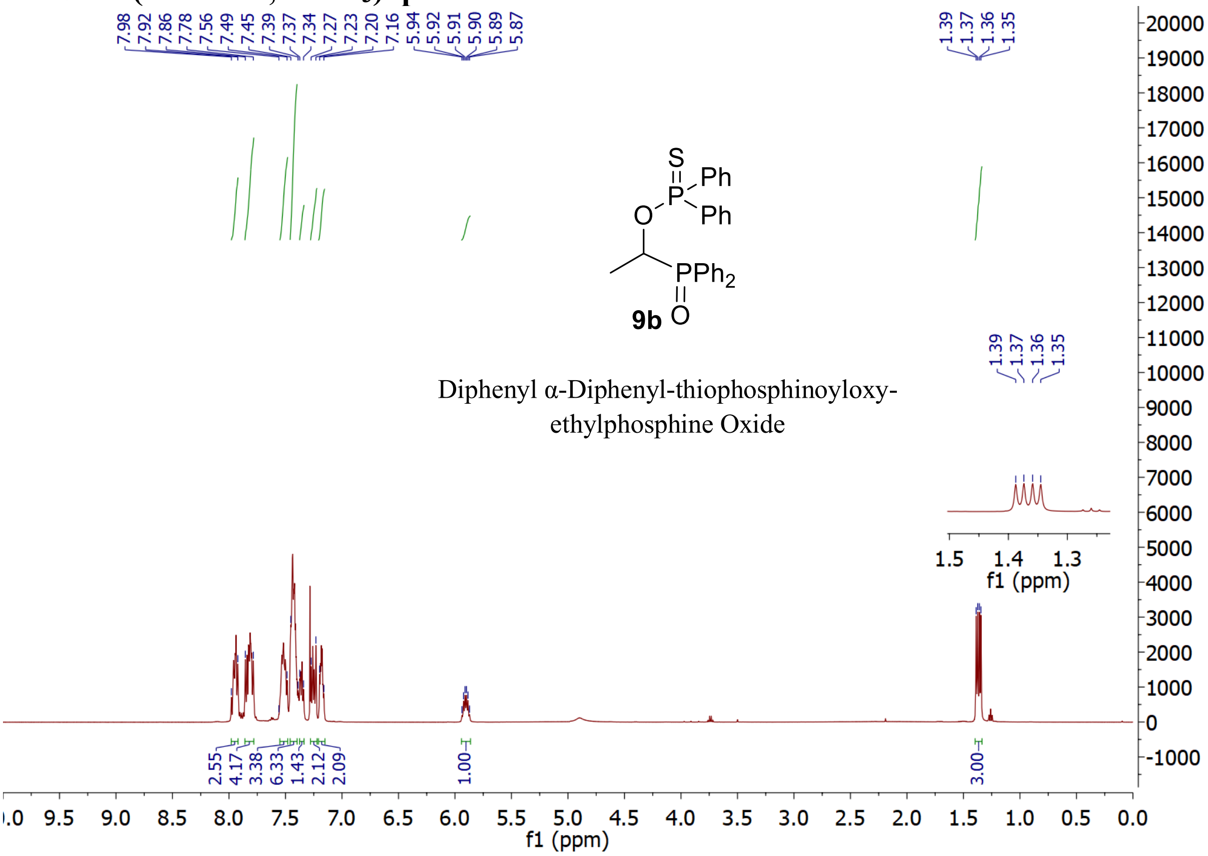

^{31}P {^{1}H} NMR (122 MHz, $CDCl_3$) spectra for 12a

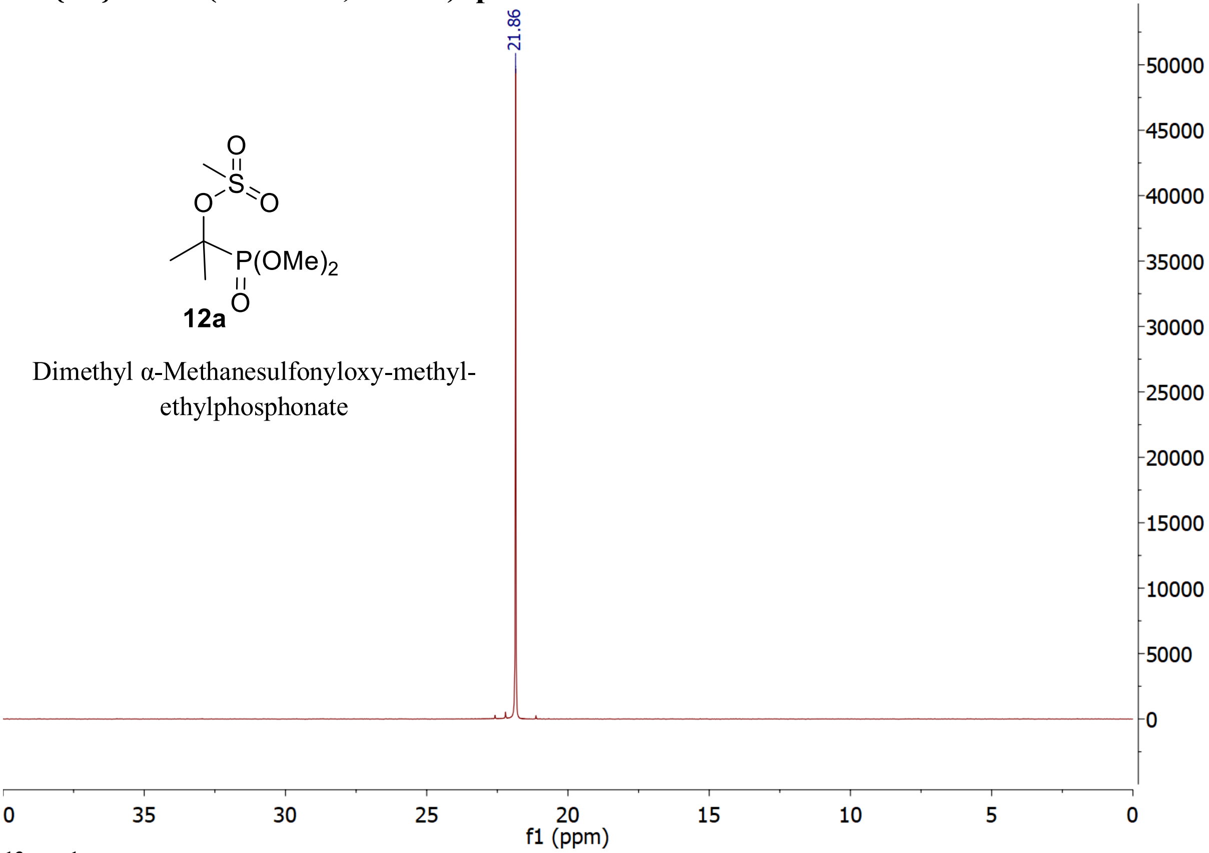

^{13}C {^{1}H} NMR (75 MHz, $CDCl_3$) spectra for 12a

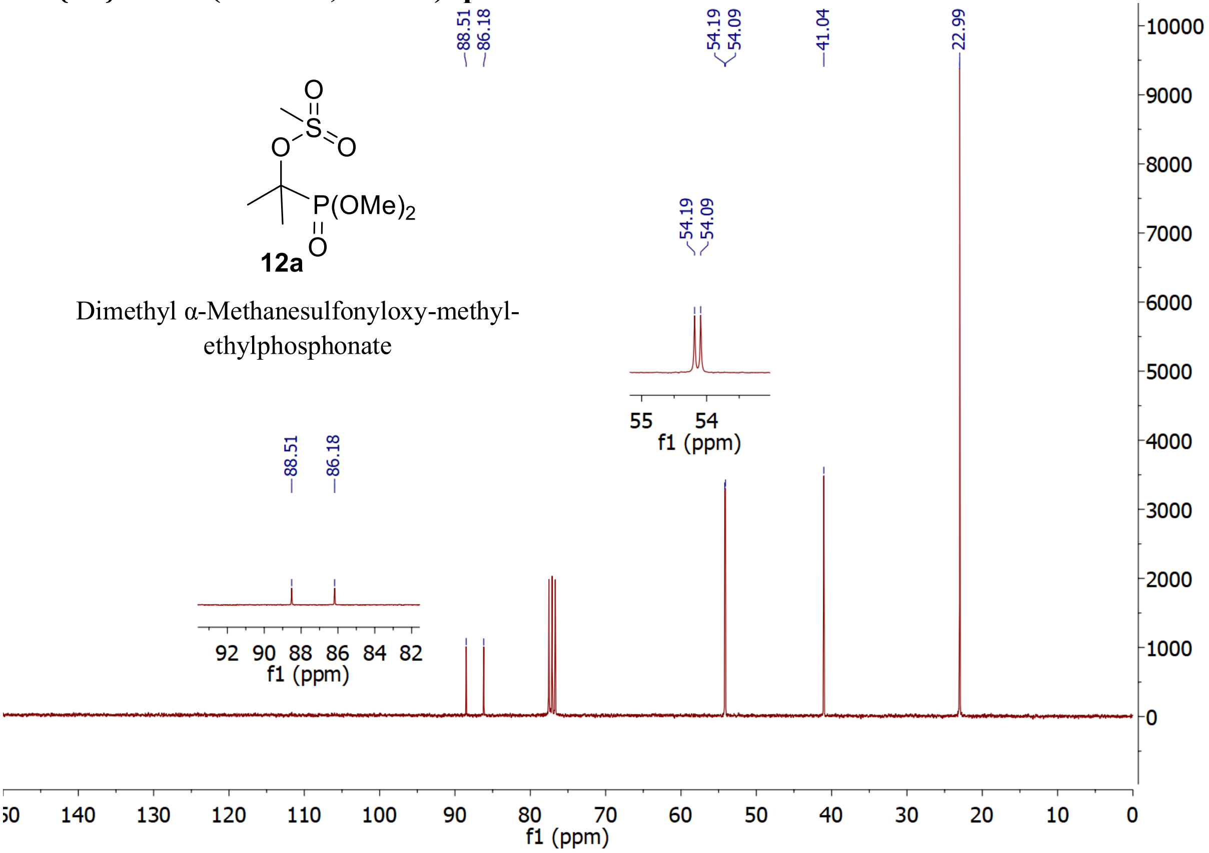

^{1}H NMR (300 MHz, $CDCl_3$) spectra for 12a

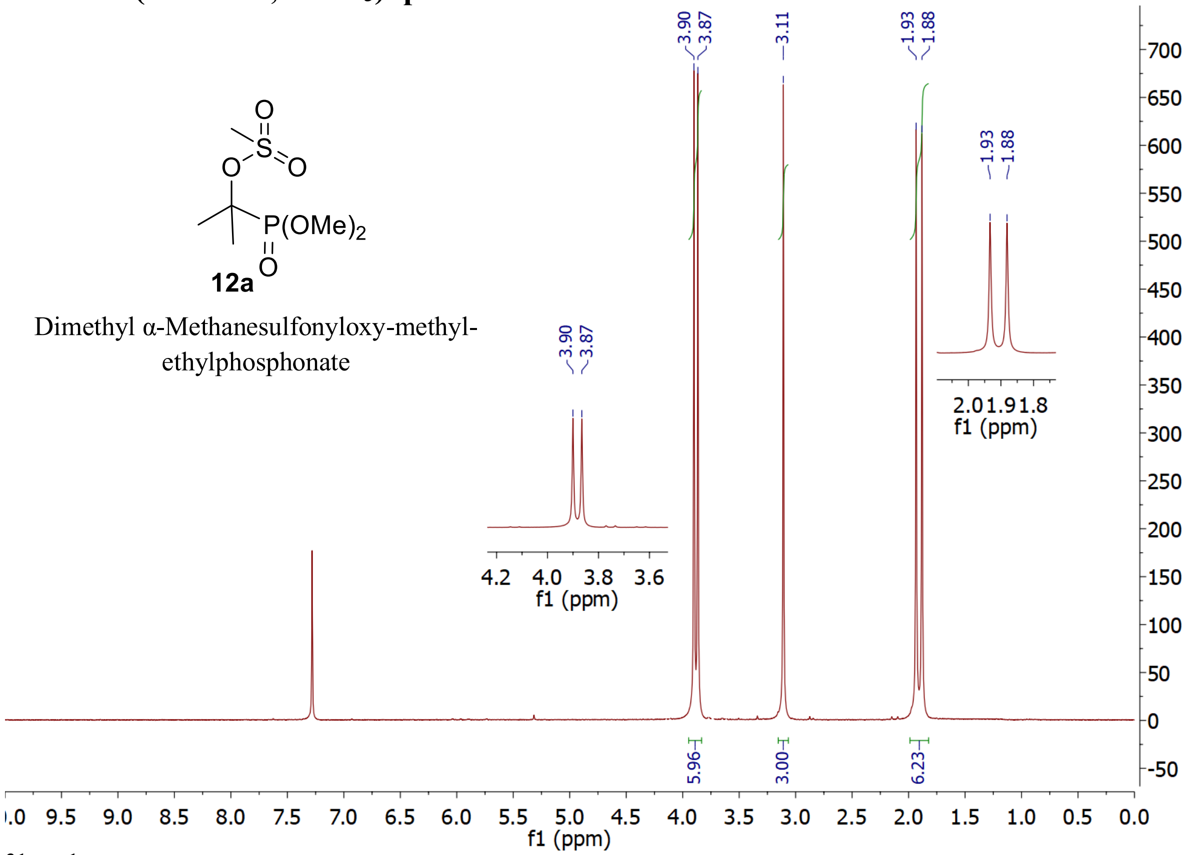

^{31}P {^{1}H} NMR (202 MHz, $CDCl_3$) spectra for 12b

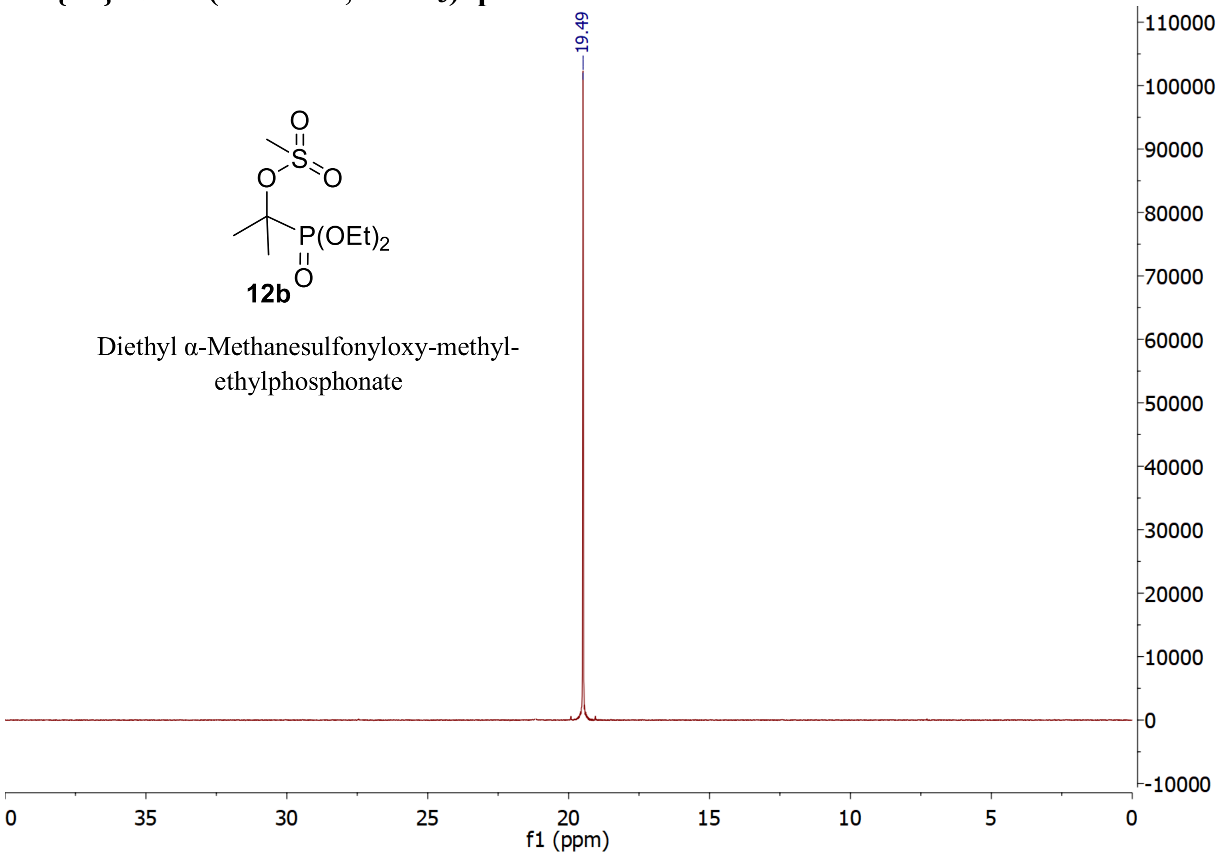

$^{13}C\ \{^{1}H\}$ **NMR (75 MHz, $CDCl_3$) spectra for 12b**

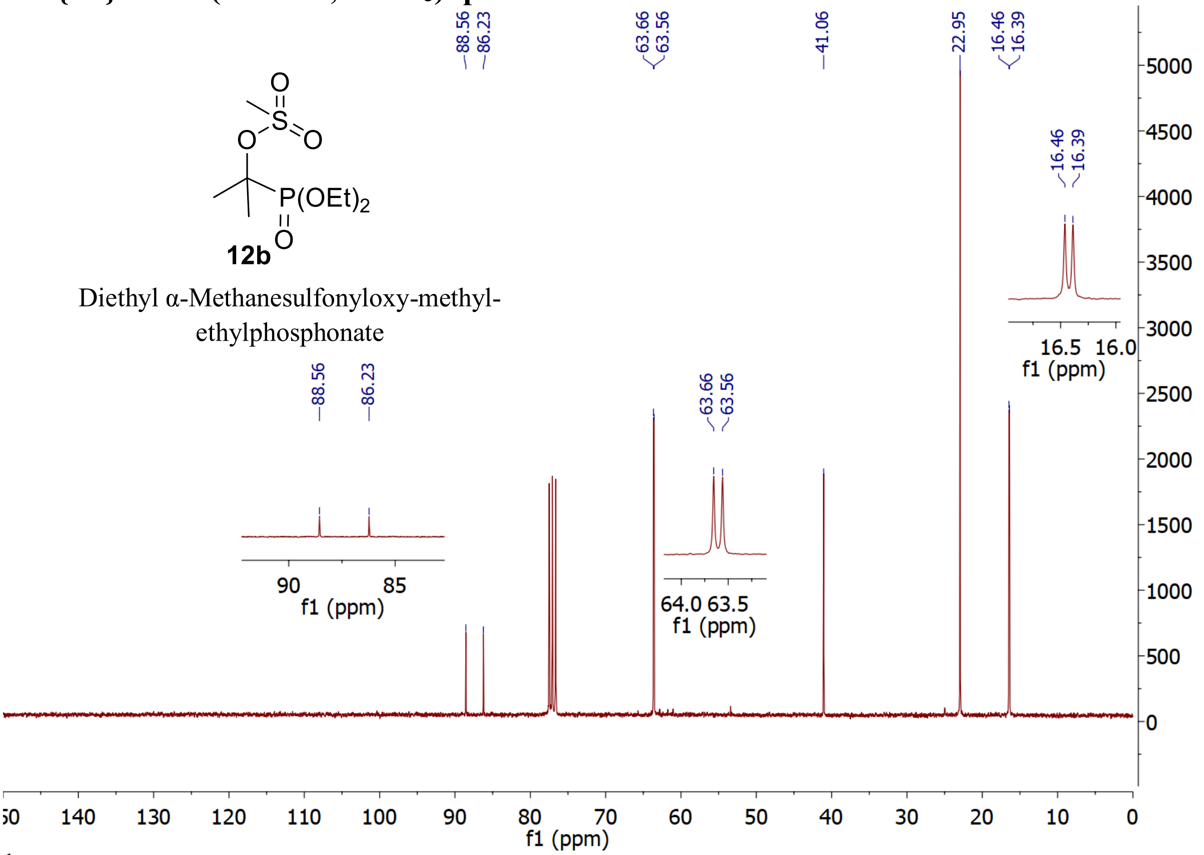

^{1}H **NMR (500 MHz, $CDCl_3$) spectra for 12b**

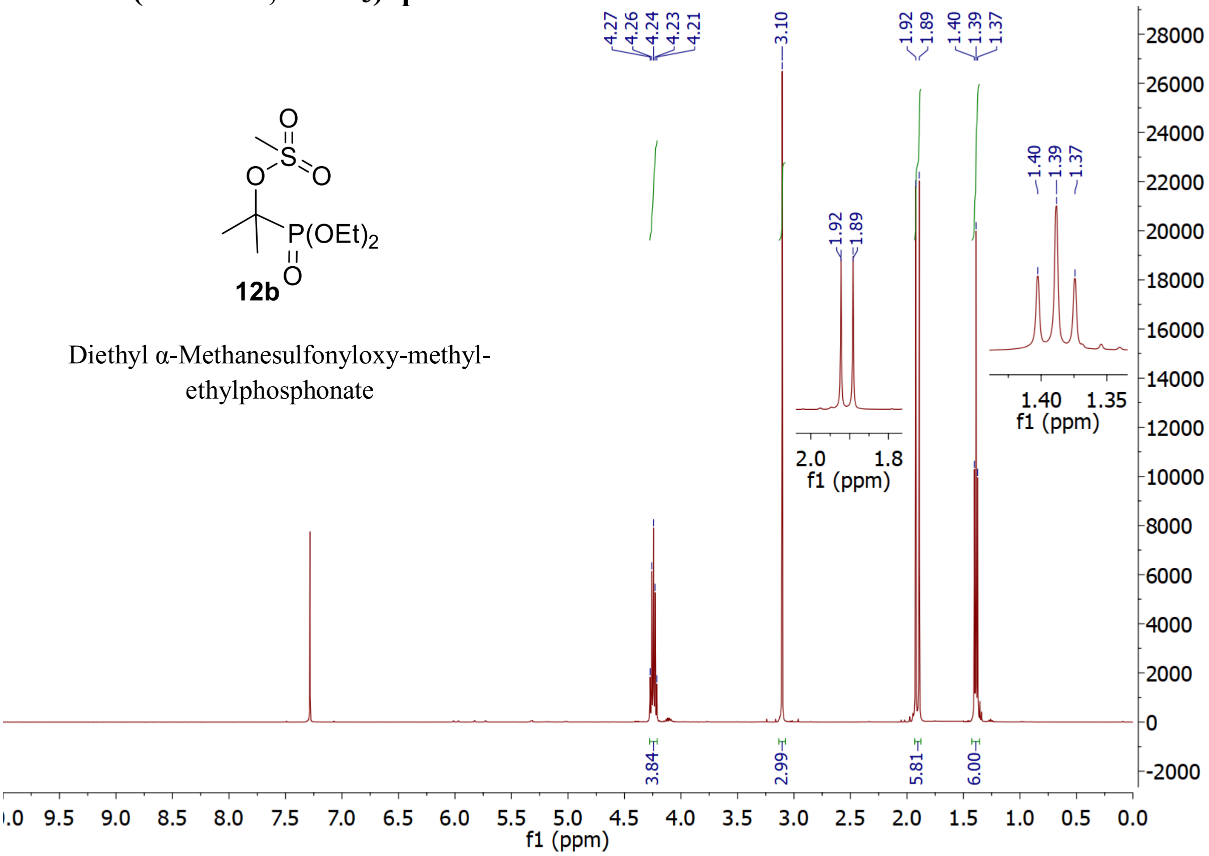

Supplement: Supplementary file 1 [file pharmaceuticals-19-00396-s001.zip › pharmaceuticals-4147883-supplementary.pdf]
